# Light‐Cleavable Auxiliary for Diselenide–Selenoester Ligations of Peptides and Proteins

**DOI:** 10.1002/chem.202301253

**Published:** 2023-07-18

**Authors:** Maximilian Schrems, Alexander V. Kravchuk, Gerhard Niederacher, Florian Exler, Claudia Bello, Christian F. W. Becker

**Affiliations:** ^1^ Institute of Biological Chemistry Faculty of Chemistry University of Vienna Währinger Str. 38 1090 Vienna Austria; ^2^ Interdepartmental Research Unit of Peptide and Protein Chemistry and Biology Department of Chemistry “Ugo Schiff” University of Florence via della Lastruccia 13 50019 Sesto Fiorentino Italy; ^3^ Vienna Doctoral School in Chemistry (DoSChem) University of Vienna Währinger Str. 42 1090 Vienna Austria

**Keywords:** auxiliary mediated ligation, chemical protein synthesis, diselenide-selenoester ligation, G-CSF, selenocysteine protecting groups

## Abstract

Diselenide–selenoester ligations are increasingly used for the synthesis of proteins. Excellent ligation rates, even at low concentrations, in combination with mild and selective deselenization conditions can overcome some of the most severe challenges in chemical protein synthesis. Herein, the versatile multicomponent synthesis and application of a new ligation auxiliary that combines a photocleavable scaffold with the advantages of selenium‐based ligation strategies are presented. Its use was investigated with respect to different ligation junctions and describe a novel para‐methoxybenzyl deprotection reaction for the selenol moiety. The glycine‐based auxiliary enabled successful synthesis of the challenging target protein G‐CSF.

## Graphical abstract

**Straightforward access** to seleno‐based light‐cleavable auxiliaries, including diverse amino acids, facilitates access to challenging protein targets via diselenide–selenoester ligations (DSL) and expressed protein selenoester ligations (EPSL).

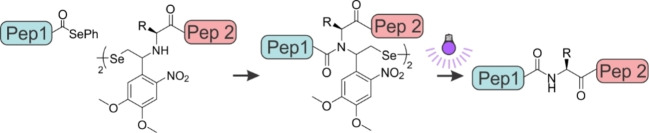

## Introduction

**Scheme 1:**
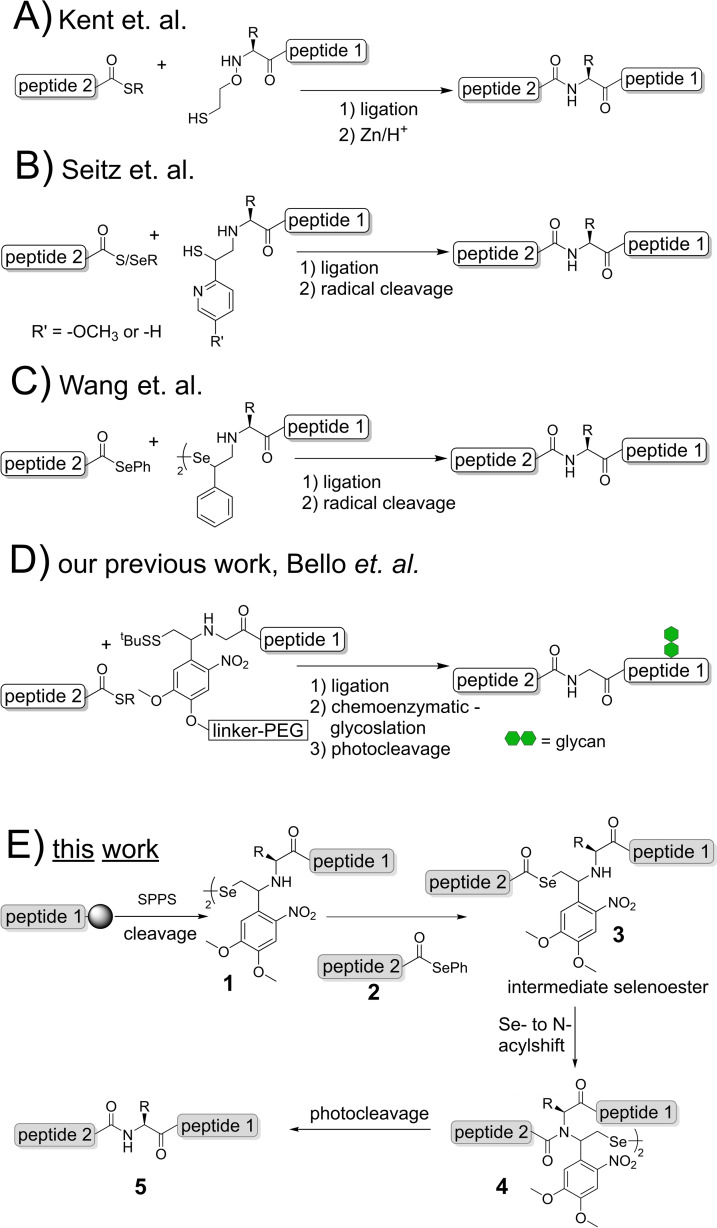
Overview of: A–D) auxiliary mediated native chemical ligation approaches; E) the seleno‐auxiliary mediated native chemical ligation investigated in this work.

Strategies that expand the scope of native chemical ligation (NCL)^[1]^ beyond cysteine‐containing junctions were investigated since the introduction of NCL into peptide chemistry in 1994 (Scheme 1A). These efforts have led to different approaches with the most promising being ligation auxiliaries^[2]^ and thiolated amino acid analogues.^[3]^ The elegance of the auxiliary method lies in its, theoretically, universal applicability with respect to the ligation junction. However, as realized over the past two decades, sterically demanding amino acids at the ligation site, especially on the C‐terminal segment, can lead to severely reduced ligation efficiencies. Lately, efforts to circumvent this problem show encouraging results (see Scheme 1B, C). Seitz and coworkers developed a pyridine‐containing auxiliary that is capable of supporting ligations at sterically hindered junctions.^[4]^ Here, intramolecular base catalysis accelerates the S‐ to N‐acyl shift, and thereby enables ligation even at β‐branched amino acids or proline (Scheme 1B). Wang and coworkers recently introduced a selenium‐containing auxiliary that, in combination with a selenoester peptide, allows rapid reaction at sterically complex junctions (Scheme 1C).^[5]^

Both auxiliaries are cleaved via a radical‐induced mechanism, which raises challenges with internal cysteine residues.

Thiolated amino acids can easily be introduced during solid phase peptide synthesis (similar to cysteine) and allow ligation at a quite large variety of different ligation junctions.

However, availability of suitably protected building blocks limits their use in peptide and protein synthesis as these thiolated amino acids are only accessible via multistep synthesis routes.^[3a]^ Subsequent desulfurization converts thiolated amino acids back to the original, (non−)proteinogenic amino acid. Slow ligation kinetics at sterically hindered positions have also hindered wider application for some of them.^[3a]^ Recently, extension of NCL towards the selenium based diselenide‐selenoester ligation (DSL) enabled the development of selenated amino acid analogues^[6]^ that have shown exceptionally good ligation efficiencies, even at the notoriously difficult Pro‐Pro junction.^[7]^

Selenium‐based ligation strategies^[8]^ exploit the rapid reaction of selenocysteine with a suitable acyl donor, preferably a selenoester, at much lower concentration^[9]^ and at lower pH than typically used in thiol‐based NCL reactions. This method, in combination with exceptionally mild and selective deselenization conditions,^[10]^ has considerably extended the application of ligation reactions to previously unattainable targets.^[11]^ Its extension towards protein semi‐synthesis by expressed protein selenoester ligation (EPSL) has further broadened the scope of use of seleno‐auxiliary building blocks in peptide and protein ligations.^[12]^

In this work we explore the combination of auxiliary‐mediated ligation reactions with selenium chemistry. Based on a ligation auxiliary previously developed by us[^2e^, ^2f^] (see Scheme 1D) our requirements were: a) incorporation of a β‐selenol moiety in the auxiliary scaffold, b) cleavage via fast and mild photoirradiation compatible with all functional groups in peptides and proteins, c) straightforward attachment as stable building block, using standard Fmoc‐based solid phase peptide synthesis (SPPS), d) the option to functionalize the auxiliary, for example to attach solubilizing tags such as polyethylene glycol or other probes for chemoenzymatic glycosylation and/or to monitor the reaction (Scheme 1D, E).[^2e^, ^11^]

We established the synthesis of such a new seleno‐containing ligation auxiliary, explored its scope in ligation reactions on a series of test peptides and eventually utilized it to access a protein target for which attempts using sulfur‐based auxiliaries failed so far.^[11]^ Reaction scope was mainly limited due to the slow shift of the acyl group from the selenium to the amino group to give the native amide bond, especially on sterically demanding ligation sites. At the same time, we were able to establish the successful synthesis of the G‐CSF aglycon protein by semi‐synthesis using a seleno‐auxiliary supported EPSL strategy.

## Results and Discussion

**Scheme 2:**
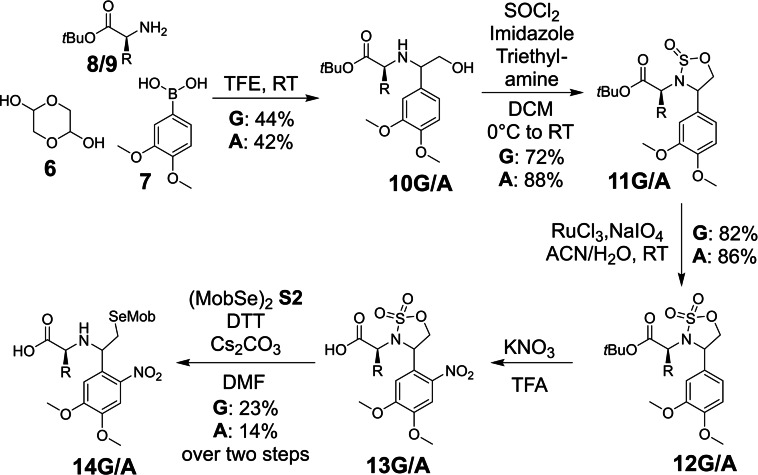
Synthesis scheme of Gly(SeAUX) **14 G** (R=H), Ala(SeAUX) **14 A** (R=CH_3_).

We envisioned a flexible synthesis that would allow incorporation of different amino acid scaffolds. Therefore we started out with the Petasis three‐component reaction to directly access the N‐arylated α‐hydroxy amino acid (Scheme 2).^[13]^ Here glycolaldehyde, added to the reaction in form of its dimer **6**, reacts with a *tert*‐butyl protected amino acid (glycine (G) **8** or alanine (A) **9**) and 3,4‐dimethoxyphenylboronic acid **7** to give the respective amino acid **10 G/A**. In our hands the best results were obtained with TFE (2,2,2‐trifluorethanol) as solvent and when using the free amino acid instead of its HCl salt. The amino acid used during this initial step determines the final N‐terminal amino acid at the ligation junction, and thereby all amino acids, except proline due to it being a secondary amine, can be theoretically incorporated. However, limitations during synthesis, induced by compatibility of side chain protecting groups with the employed reaction conditions as well as potential racemization have to be considered when establishing such a synthesis route. We also took into account potential ligation efficiencies and therefore selected glycine **8** and alanine **9** for these initial studies.

To our surprise, protection of the secondary amine obtained in step 1 (Scheme 2) with Boc anhydride and subsequent conversion of the alcohol into an alkyl chloride for further derivatization yielded not the expected product, but an oxazolidinone. A similar reaction was observed by Ghosh et al.^[14]^ Opening of this heterocycle was shown to be possible,^[15]^ but in our case reaction with various seleno‐nucleophiles (4‐methoxybenzyl selenolate or dilithium diselenide) did not result in the formation of the desired product. Thus, we adopted the strategy used by Ghosh and converted the α‐hydroxy amino acid **10 G/A** into a sulfamidite **11 G/A**.^[16]^ Further oxidation via Ruthenium catalysis[^14^, ^17^] gave sulfamidate **12 G/A**, which is stable during the subsequent nitration reaction to form compound **13 G/A**. Ring‐opening of the sulfamidate, using 4‐methoxybenzyl selenolate (**S2**), gave the desired glycine seleno‐auxiliary (Gly(SeAUX)) **14 G** and alanine seleno‐auxiliary (Ala(SeAUX)) **14 A** (Scheme 2).

In the synthesis of Gly(SeAUX) **14 G** no chiral starting materials were used during the Petasis reaction, where a stereocenter is formed, giving a racemic mixture of products. Studies conducted by Dawson and coworkers^[18]^ showed that the configuration of the chiral center of a comparable auxiliary has no influence on ligation yields. Therefore, no efforts were taken to resolve the two diastereomers formed after coupling to peptides.

During the Petasis reaction to form intermediate **10 A**, enantiomerically pure L‐Alanine‐O*tBu* gave a mixture of two diastereomers in nearly 1 : 1 ratio (52 : 48, see Supporting Information). No attempt was made to separate these during further synthesis and final product **14 A** was used as a diastereomeric mixture for this preliminary study.

**Scheme 3:**
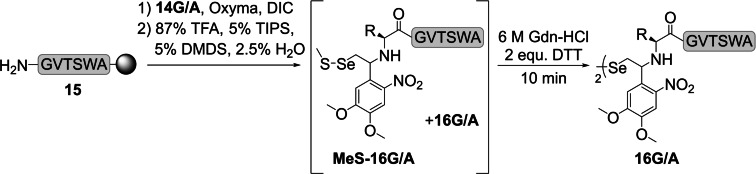
Coupling of seleno‐auxiliaries **14 G**/**A** to peptide **15** via standard Fmoc‐SPPS chemistry and cleavage from the resin.

Next, we tested coupling of these auxiliaries to peptide **15** (GVTSWA), which was synthesized on a TentaGel® R PHB resin, employing Fmoc‐based SPPS. Coupling of the Gly(SeAUX) building block **14 G** and Ala(SeAUX) building block **14 A** (1.25 and 1.9 equiv respectively) on resin was achieved under standard SPPS conditions, using Oxyma/DIC as coupling reagents (Scheme 3, and Supporting Information 6.4–6.5).

A number of relatively mild methods have been developed to remove the 4‐methoxybenzyl (Mob or PMB) selenol protecting group during or subsequent to global deprotection.[^8a^, ^19^] In our experience, the effectiveness of some of these conditions are peptide (sequence) dependent, while others are more universally applicable. One of the latter is the addition of DTNP (2,2′‐dithiobis(5‐nitropyridine)) to the cleavage cocktail.^[19b]^

While this robust method works reliably for numerous peptides, the formed 2‐thio(5‐nitropyridyl) adduct can be difficult to remove in some cases^[19c]^ and excess DTNP can be problematic during purification, as it does not readily dissolve in aqueous buffers or diethyl ether.

We discovered that addition of 5 % dimethyl disulfide (DMDS) to the standard cleavage cocktail removes the Mob group very efficiently, producing a mixture of diselenide **16 G/A** and the methyl sulfide adduct **MeS‐16 G/A**, which is easily converted into the diselenide **16 G/A** via brief incubation in DTT (dithiothreitol) or TCEP (tris(2‐carboxyethyl)phosphine) containing buffer (Scheme 3). This method simplifies the liberation of Mob‐protected selenols significantly, as no additional considerations regarding reaction conditions, such as equivalents used, reaction time or heating, and purification have to be taken into account. For all cases we tested, these conditions reliably produced Mob‐deprotected peptides and allowed to purify them either as monomers or dimers. Cleavage from the resin under these conditions gave **16 G** and **16 A** in 24 % and 23 % purified yield, respectively.

To exclude racemization of the amino acid stereocenter of Ala(SeAUX), either during synthesis, coupling or global deprotection, co‐injections of manually synthesized **25** (AGVTSWA) and **Da‐25** (aGVTSWA, with a representing D‐ alanine (a) at the N‐terminus) with photocleaved peptide **16 A** (AGVTSWA) were performed. We could show that, as expected, the photocleaved peptide **16 A** coelutes with the synthesized peptide **25**, therefore no racemization occurred (Figure S55).

**Figure 1:**
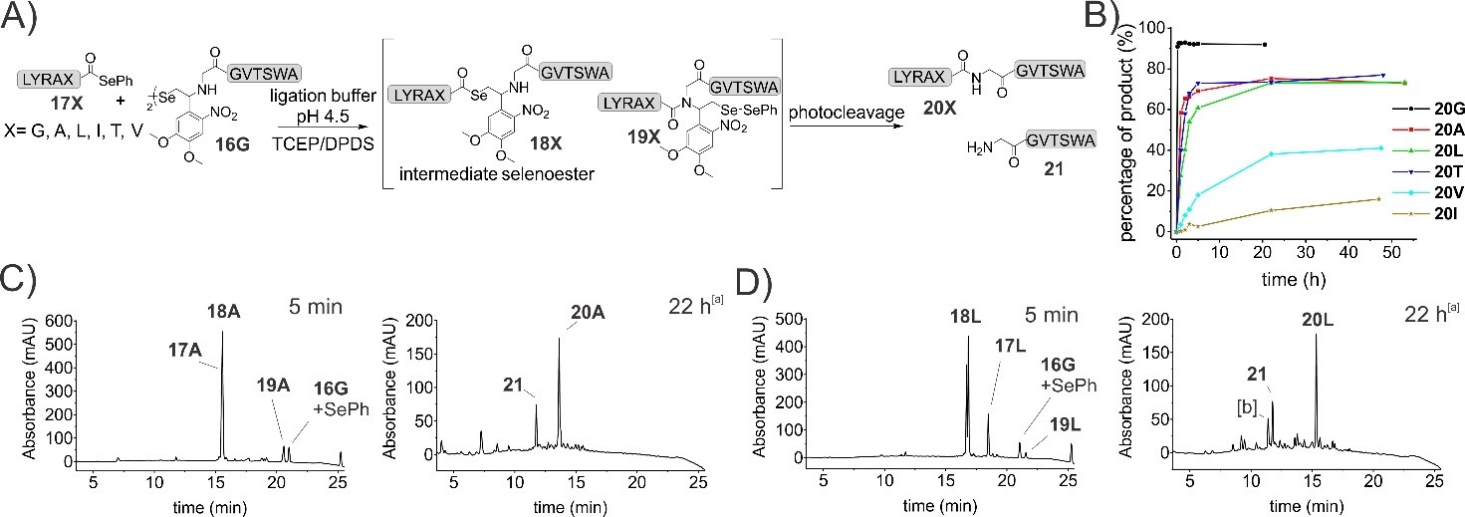
A) Glycine‐Auxiliary mediated ligation of peptides **17 X** and **16 G**; B) Ligation over time at different C‐terminal junctions after photocleavage; C) and D) Ligation of peptide **16 G** after 5 min (no photocleavage or quench) and after 22 h; with alanine selenoester **17 A** (C) and with leucine selenoester **17 L** (D); [a] sample irradiated with UV light for 3 min, then 2.5 % hydrazine added, [b] hydrolyzed selenoester.

In order to fully harness the advantages of DSL, combination of the seleno‐auxiliaries **16 G/A** with a selenoester acyl donor during ligation is necessary. Thus, peptides **17 X** (Figure 1) were synthesized from their corresponding hydrazides by activation with acetylacetone^[20]^ and converted into the selenoester by incubation with DPDS (diphenyl diselenide) and TCEP.[^11^, ^21^]

### Ligation efficiency of Gly(SeAUX)

**Figure 2:**
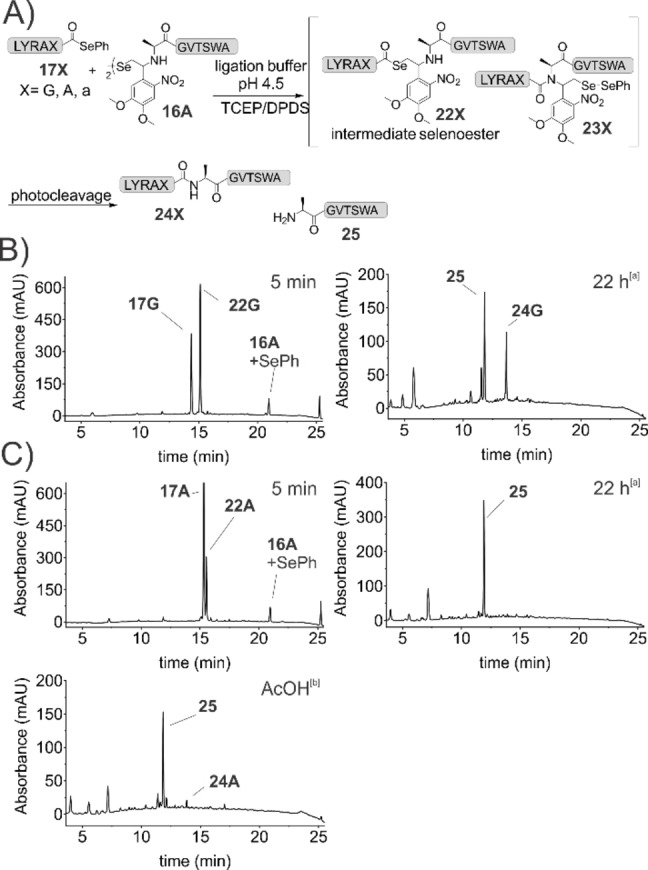
A) Alanine‐Auxiliary mediated ligation of peptides **17 X** and **16 A**; Ligations after 5 min (no photocleavage or quench) and after 22 h; B) with glycine selenoester **17 G**; C) with alanine selenoester **17 A**; [a] sample irradiated with UV light for 3 min, then 2.5 % hydrazine added, [b] after 1 h of incubation, sample treated with 5 % AcOH in DMF for 20 h, lyophilization, UV irradiation, then 2.5 % hydrazine added.

Considering the advantage of the diselenide/selenoester ligation to reduce selenoester hydrolysis by carrying out ligation at acidic pH, we performed the initial ligation reactions with LYRAG selenoester **17 G** and Gly(SeAUX)‐peptide **16 G** at pH 5.2, using 200 mM bis‐Tris buffer containing 6 M Gdn‐HCl, 30 mM TCEP and 20 mM DPDS.^[9]^ Under these conditions, we quickly realized that premature removal of the seleno‐auxiliary occurs, yielding the GGVTSWA peptide **16 G** without the auxiliary. A plausible explanation for this observation is based on a mechanism proposed by Melnyk et al. for their N‐selenoethyl cysteine redox switch system.^[22]^ There, an intermolecular nucleophilic attack of the selenolate leads to cleavage of the carbon‐nitrogen bond. In response to this observation, we reduced the amount of TCEP to 0.5 equiv in relation to the peptide and confirmed that this minimizes undesired SeAUX removal. After 5 min of incubation the intermediate selenoester **18 G** was observed (see Figure 1A). To ensure complete reaction, a stock solution of 15 mM TCEP and an excess of 50 mM DPDS in degassed ligation buffer was incubated for 10–15 min. A calculated 0.5 equivalents (based on TCEP concentration) of this stock solution were then added to all following ligation reactions, except when otherwise noted. Thereby we achieved a sufficient amount of phenylselenol/phenylselenolate in solution. This approach is also based on NMR studies performed by Chisholm et al.^[9]^ that link the amount of phenylselenol/phenylselenolate present in solution to the initial TCEP concentration. We were able to severely reduce premature auxiliary cleavage by applying lower TCEP/phenylselenol concentrations, however it cannot be completely avoided. Complete omission of TCEP resulted in slower formation of the transient selenoester and thereby slower formation of product and lower yields, even after extending reaction times, compared to the TCEP‐containing ligations (see Table S1). While closely monitoring these reactions, we also observed a surprisingly high stability of the intermediate selenoester **18 G** (Figure 1A, C, D and Scheme S5), which we could easily detect in HPLC and MS analysis. Similar observations have been made for other auxiliary‐mediated ligations as the often slow rearrangement of the thioester to the amide bond is a well‐known complication.^[2a]^ Here we confirmed that this is also the case for the selenium based reactions.^[5]^ Several strategies have been reported to resolve this sensitive intermediate by acidification of the reaction solution,^[2a]^ addition of DMF/DMF with acetic acid,[^5^, ^23^] addition of a catalyst^[6e]^ or change of the ligation auxiliary moiety.^[4]^ In our hands, higher yield and faster ligation was observed by lowering the pH (see below). Addition of DMF with acetic acid, during ligation with Ala(SeAUX)‐peptide **16 A**, to the formed intermediate selenoester **22 X**, in cases with slow ligation or no product formation at all, gave marginally more product (Figure 2). Addition of imidazole to the ligation led to an increased hydrolysis rate of the selenoester, and was therefore not suitable for application under the employed conditions (data not shown).

Based on the fact that the pKa value of the selenol group in selenocysteine (Sec) is typically 3 pKa units lower than that for corresponding thiol groups in cysteine,[^3b^, ^24^] we next investigated the influence of pH on the ligation reaction, in the absence of TCEP (Figure S37). At pH 7 the starting peptide selenoester **17 X** and the quickly formed intermediate selenoester **18 G** (Figure 1) are hydrolyzed after extended reaction time, with the formation of only trace amounts of product. At pH 5 the formation of product is increased, but hydrolysis of the selenoester is still observed. While at pH 4 the selenoester is more stable, less product is formed, presumably due to the fact that insufficient amounts of phenylselenolate are generated by hydrolysis^[9]^ and the N‐terminus is protonated to a higher extend. Accordingly, we chose a pH of 4.5 (sodium acetate buffer) for our further experiments, as the best possible compromise between reaction speed and low levels of hydrolysis.

To obtain the final product, cleavage of the auxiliary was achieved via photoirradiation with 365 nm UV light for 3–5 min directly after ligation, with no need for DPDS extraction, addition of further reagents or purification. However, addition of a 2.5 % hydrazine solution in water quenches any reactive species resulting from the auxiliary after cleavage and the reaction product can be directly analyzed or purified via HPLC. Next, we tested the ligation efficiency of the Gly(SeAUX) with different selenoester peptides, covering simple amino acids at the C‐terminus, such as Gly and Ala, as well as the more challenging β‐branched amino acids Thr, Ile and Val (Figure 1A). In order to allow a straightforward evaluation of these experiments, aliquots of the ligation mix were diluted, irradiated and quenched as mentioned above, to directly provide yields of the ligation and the cleavage reaction.

Ligation with a C‐terminal glycine selenoester proceeded smoothly, whereas sterically more demanding amino acids led to a decrease in reaction speed and yield (Figure 1A and B). Interestingly, the rate of formation of intermediate selenoester **18 X** was very similar for all amino acids, but the Se‐ to N‐acyl shift was clearly the rate determining step. Due to the extended reaction times necessary to achieve the acyl transfer, premature hydrolysis reduces ligation yields.

Without the addition of TCEP and DPDS to the reaction mixture considerably lower yields, with exception for glycine selenoester **20 G**, were observed (Table S1). This shows the necessity of externally added phenylselenolate to swiftly initiate the formation of intermediate selenoesters between the two peptides.

### Ala(SeAUX) mediated ligations

In a next step, we investigated the reactions with Ala(SeAUX)‐peptide **16 A** to assess the impact of sidechains on this selenol/selenoester auxiliary ligation.

Similar to ligations with Gly(SeAUX), the intermediate selenoester **22 X** is formed fast, but rearrangement to the amide is even slower, most likely due to the additional steric hindrance (Figure 2B–C). For the Gly‐Ala junction a yield of 33 % after 22 h is observed (Figure 2B), compared to 92 % for the Gly‐Gly junction (Figure 1). At the Ala‐Ala junction only selenoester intermediate **22 A** was found but no product formation could be observed (Figure 2C). To facilitate the Se‐ to N‐acyl shift we investigated the effect of addition of 5 % acetic acid in DMF to the ligation mix.[^5^, ^23^] Contrary to the observation by Wang et al., we only observed formation of marginal amounts of product (Figure 2C). To test effects of side chain positioning of the C‐terminal amino acid, a ligation using selenoester LYRAa‐SePh **17 A**, containing D‐alanine (a) at the C‐terminus, was performed. As no influence on the ligation was observed, we reason that steric hindrance independent of configuration of the C‐terminal amino acid is responsible for the decrease in ligation yields.

Despite the above described limitations of the SeAUX, a potential benefit of the glycine‐based auxiliary lies within its superior reaction kinetics and yields when compared to its sulfur analog.

Therefore we applied the Gly(SeAUX) to a highly challenging protein target for which the sulfur analog (Scheme 1D)^[2e]^ has previously failed, Granulocyte Colony‐Stimulating Factor (G‐CSF).^[11]^ To take full advantage of the Gly(SeAUX) we generated the N‐terminal G‐CSF segment (aa 1–124) with a C‐terminal leucine selenoester to carry out an auxiliary‐enabled expressed protein selenoester ligation (EPSL).

**Figure 3:**
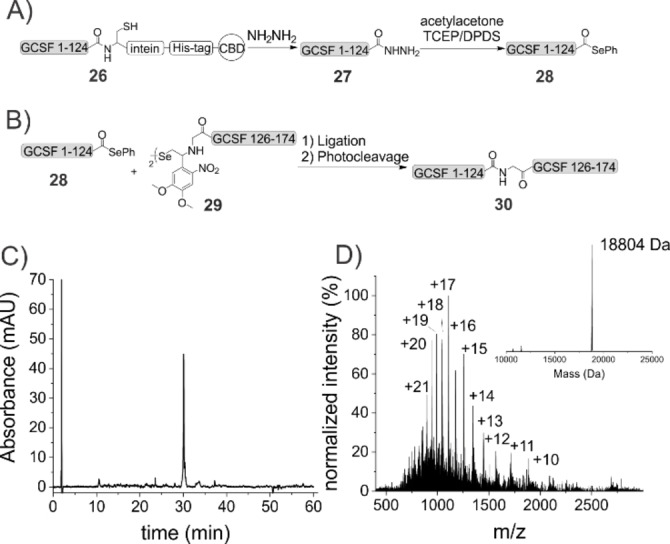
A) Synthesis of G‐CSF 1–124 selenoester **28**; B) Expressed selenoester‐ligation of Gly(SeAUX)‐G‐CSF 126–174 **29** and selenoester **28**; C) Chromatogram of purified product **30**; D) ESI‐MS of product **30**; calculated mass: 18804 Da.

Briefly, a G‐CSF1–124‐*Mxe*Intein‐His‐tag fusion construct **26** was expressed in *E. coli*. Hydrazinolysis and preparative HPLC purification gave the G‐CSF 1–124 hydrazide **27** in a yield of 2.7 mg per liter of culture (Figure 3A). Conversion of the hydrazide into selenoester **28** was done via generation of a pyrazole intermediate by incubation in buffer containing 6 M Gdn‐HCl, 200 mM HEPES, 200 mM TCEP, 50 mM DPDS and 310 equiv acetylacetone (Figure 3A).^[11]^

Although HPLC analysis gave a single peak, mass spectrometry data indicated a mixture of the desired product and a side product. Further analysis on a high resolution mass spectrometer shows mainly the expected selenoester. The mass of the side product, compared to selenoester **28**, was 157 Da lower, which corresponds to the mass of phenyl selenolate (Figure S36). We reason that this species forms during analysis due to fragmentation, possibly via cleavage of the carbon‐selenium bond and formation of an acylium ion. A similar observation was also made, to a smaller extend, during a previous synthesis^[11]^ and in literature.^[4]^

The C‐terminal segment 126–174 was synthesized via automated SPPS and the Gly(SeAUX) building block **14 G** was manually coupled.

Based on the optimized ligation conditions identified above, we incubated 1 equiv of G‐CSF 1–124 selenoester **28** with 2.5 equiv of the Gly(SeAUX)‐G‐CSF 126–174 peptide **29** in degassed ligation buffer. 0.5 equiv of TCEP/DPDS were added (Figure 3B). After 5 min of ultrasonication, the rapid formation of intermediate selenoester was observed (Figure S53A). As above, we found the Se‐ to N‐acyl shift to be the rate‐determining step in this reaction. After 50 h the reaction was quenched by the addition of TCEP, followed by photocleavage and addition of hydrazine. Analysis revealed the formation of product, as well as hydrolyzed selenoester and SeAUX cleaved N‐terminal fragment **29** amongst other unidentified side products. Purification via HPLC gave G‐CSF 1–174 **30** in 20 % yield (Figure 3C and D).

## Conclusions

These results clearly show the benefits of selenium based ligation auxiliaries, as a previous synthesis of G‐CSF using the mercapto‐auxiliary at the same ligation junction failed to produce any product.^[11]^ However, our results also clearly indicate the shortcomings of the auxiliary‐based reaction described here, which are mainly related to the rate‐limiting slow formation of the amide bond at ligation sites that are sterically more demanding than Gly‐Gly sites. This challenge can be overcome by different auxiliary designs, for example ones that do not attach the amino group directly to the benzylic position of the auxiliary scaffold and/or support amide bond formation via an intramolecular base.[^4^, ^5^] Even though such auxiliaries work well for many ligation junctions their radical‐based cleavage conditions are not easily compatible with all amino acids and peptide modifications in contrast to the photocleavable, selenium‐based auxiliary described in this work that further expands the set of available ligation strategies.

The synthesis of the selenol‐containing auxiliary easily allows the use of different amino acids as they can be added as starting material to the initial Petasis three‐component reaction. Coupling of the resulting auxiliary is conveniently achieved using standard Fmoc‐based SPPS conditions. While investigating the cleavage from resin and deprotection of the selenol group, we discovered that addition of dimethyl disulfide to the acidolytic cleavage cocktail facilitated the mild and efficient removal of the Mob group, and significantly simplified handling and purification of the obtained crude peptides. The successful ligation of Gly(SeAUX)‐peptides and selenoesters with various C‐terminal amino acids shows the feasibility of the presented strategy. Mild in situ cleavage of the auxiliary was achieved via UV‐irradiation of the crude ligation mixture and proved to be selective in presence of particularly sensitive amino acids, such as cysteine or methionine. We reason that development of our selenium‐based scaffold towards a photocleavable, α‐unsubstituted auxiliary[^4a^, ^5^, ^25^] has the potential to alleviate problems observed here and will be the subject of future investigation.

## Experimental Section

### Synthesis of Gly(SeAUX) building block 14 G

***tert*** **‐butyl‐(1‐(3,4‐dimethoxyphenyl)‐2‐hydroxyethyl)glycinate (10 G)**: 1,4‐dioxane‐2,5‐diol (glycolaldehyde dimer, 66 mg, 0.55 mmol, 0.5 equiv) **6** and (3,4‐dimethoxyphenyl) boronic acid (200 mg, 1.1 mmol, 1 equiv) **7** were suspended in 2,2,2‐trifluorethanol (2 mL). Upon addition of glycine *tert*‐butyl ester **8** (167 μL, 160 mg, 1.2 mmol, 1.1 equiv) the suspension turned clear. After 20 h stirring at room temperature, the solvent was evaporated and the concentrate taken up in ethyl acetate. The organic phase was washed three times with 1 M aqueous NaOH, then two times with MQ‐H_2_O and once with brine. The organic phase was dried over Na_2_SO_4_ and the solvent evaporated under reduced pressure. Column chromatography using neutralized silica gel (via addition of triethylamine to the silica gel slurry) and 5 % MeOH in DCM gave the product **10 G** in 44 % yield (150 mg, 0.48 mmol) as a brown oily liquid.

^1^H NMR (600 MHz, CDCl_3_) δ=6.95–6.92 (m, 1H), 6.88–6.81 (m, 2H), 3.89 (s, 3H), 3.87 (s, 3H), 3.85–3.79 (m, 1H), 3.77–3.69 (m, 2H), 3.32 (d, *J*=17.4, 1H), 3.21 (d, *J*=17.4, 1H), 1.44 (s, 9H). ^13^C NMR (151 MHz, CDCl_3_) δ=170.12, 148.31, 147.79, 130.38, 118.97, 110.16, 109.31, 80.79, 65.50, 63.06, 54.98, 54.91, 47.76, 27.05. HRMS (ESI): *m/z* calculated for C_16_H_25_NO_5_+Na^+^: 334.1625 [*M*+Na]^+^; found: 334.1623.

***tert*** **‐butyl‐2‐(4‐(3,4‐dimethoxyphenyl)‐2‐oxido‐1,2,3‐oxathiazolidin‐3‐yl)acetate (11 G)**: **10 G** (430 mg, 1.38 mmol, 1 equiv), imidazole (395 mg, 5.8 mmol, 4.2 equiv) and triethylamine (0.39 mL, 279 mg, 2.76 mmol, 2 equiv) were dissolved in anhydrous DCM (4.5 mL) under dry atmosphere. Thionyl chloride (0.15 mL, 246 mg, 2.1 mmol, 1.5 equiv) was added dropwise at 0 °C under stirring. After 30 min the solution was allowed to warm up to room temperature and stirred for 18 h. The reaction was quenched via addition of MQ‐H_2_O and the aqueous phase extracted with DCM. The combined organic phases were washed with brine and dried over MgSO_4_. Evaporation of the solvent under reduced pressure and short silica filtration (petroleum ether/ethyl acetate 1 : 1) gave product **11 G** as an inconsequential mixture of stereoisomers in 72 % yield (356 mg, 0.996 mmol). Diastereomeric ratio a:b 60 : 40, determined by integration of amino acid α‐protons (at 3.65 ppm and 3.42 ppm).

^1^H NMR (600 MHz, CDCl_3_, diastereomers a) δ=6.95–6.82 (m, 3H), 4.91 (dd, 1H), 4.75–4.63 (m, 1H), 4.18 (dd, 1H), 3.97–3.86 (br s, 6H), 3.63 (d, *J*=17.4, 1H), 3.42 (d, *J*=17.4, 1H), 1.42 (s, 9H). ^1^H NMR (600 MHz, CDCl_3_, diastereomers b) δ=7.01 (d, *J*=2.1, 1H), 6.95–6.82 (m, 2H), 4.75–4.63 (m, 2H), 3.91 (d, *J*=18.4,1H), 3.97–3.86 (br s, 6H), 3.42 (d, *J*=18.4, 1H), 1.44 (s, 9H). ^13^C NMR (151 MHz, CDCl_3_, for both diastereomers) δ=168.52 (b), 168.21 (a), 149.95, 149.75, 149.70, 149.63, 127.07 (a), 126.07 (b), 121.10 (b), 121.05 (a), 111.26 (a), 111.23 (b), 110.30 (b), 109.95 (a), 82.60 (b), 82.51 (a), 77.26 (a), 75.68 (b), 66.03 (b), 62.80 (a), 56.50, 56.01, 56.00, 55.97, 46.63 (a), 44.96 (b), 28.01 (b), 27.96 (a). HRMS (ESI): *m/z* calculated for C_16_H_23_NO_6_S+Na^+^: 380.1138 [*M*+Na]^+^; found: 380.1136.

***tert*** **‐butyl‐2‐(4‐(3,4‐dimethoxyphenyl)‐2,2‐dioxido‐1,2,3‐oxathiazolidin‐3‐yl)acetate (12 G)**: Starting material **11 G** (340 mg, 0.95 mmol, 1 equiv) was dissolved in acetonitrile (5.6 mL) and cooled on ice. A catalytic amount of RuCl_3_×H_2_O was added, followed by H_2_O (5.6 mL) and sodium periodate (244 mg, 1.14 mmol, 1.2 equiv). After stirring at 0 °C for 45 min, the reaction was stopped by addition of DCM and extraction of the organic phase, which was washed with brine and dried over Mg_2_SO_4_. A short silica filtration (petroleum ether/ethyl acetate 1 : 1) gave product **12 G** in 82 % yield (290 mg, 0.78 mmol).

^1^H NMR (600 MHz, CDCl_3_) δ=6.96 (d, *J*=2.1, 1H), 6.93 (dd, *J*=8.1, 2.1, 1H), 6.87 (d, *J*=8.1, 1H), 5.17 (dd, *J*=8.5, 6.9, 1H), 4.78 (dd, *J*=8.7, 6.9, 1H), 4.40 (t, *J*=8.6, 1H), 3.90 (s, 3H), 3.89 (s, 3H), 3.82 (d, *J*=17.9, 1H), 3.52 (d, *J*=17.9, 1H), 1.44 (s, 9H). ^13^C NMR (151 MHz, CDCl3) δ=166.93, 150.16, 149.86, 126.41, 120.71, 111.31, 109.85, 82.77, 72.94, 62.63, 56.04, 55.99, 45.20, 27.94. HRMS (ESI): *m/z* calculated for C_16_H_23_NO_7_S+Na^+^: 396.1087 [*M*+Na]^+^; found: 396.1088.

**2‐(4‐(4,5‐dimethoxy‐2‐nitrophenyl)‐2,2‐dioxido‐1,2,3‐oxathiazolidin‐3‐yl)acetic acid (13 G)**: **12 G** (290 mg, 0.78 mmol, 1 equiv) was added to trifluoracetic acid (TFA, 600 μL) and the solution cooled down to 0 °C with an ice bath. A solution of KNO_3_ (86 mg, 0.85 mmol, 1.1 equiv) in TFA (600 μL) was added at 0 °C and stirred for 1.5 h. The TFA was evaporated under a stream of nitrogen, the concentrate was taken up in DCM, washed with 1 M HCl followed by brine and the organic phase dried over MgSO_4_. Evaporation of the solvent under reduced pressure gave the crude product **13 G** (254 mg) that was used for the next step without further purification.

^1^H NMR (600 MHz, CD_3_OD) δ=7.77 (s, 1H), 7.69 (s, 1H), 5.77 (dd, *J*=7.4, 4.2, 1H), 5.25 (dd, *J*=9.0, 7.4, 1H), 4.44 (dd, *J*=9.0, 4.2, 1H), 4.07 (d, 2H), 3.99 (s, 3H), 3.93 (s, 3H). ^13^C NMR (151 MHz, CD_3_OD) δ=171.52, 155.62, 150.21, 141.44, 130.18, 111.59, 109.72, 74.64, 63.21, 56.98, 56.90, 48.77. HRMS (ESI): *m/z* calculated for C_12_H_14_N_2_O_9_S+Na^+^: 385.0312 [*M*+Na]^+^; found: 385.0308.

**(1‐(4,5‐dimethoxy‐2‐nitrophenyl)‐2‐((4‐methoxybenzyl)selanyl)ethyl)glycine (14 G)**: Dithiothreitol (21 mg, 0.14 mmol, 1 equiv) and diselenide **S2** (41 mg, 0.1 mmol, 0.75 equiv) were dissolved in anhydrous DMF (700 μL) under argon atmosphere and stirred for 15 min. Cs_2_CO_3_ (112 mg, 0.34 mmol, 2.5 equiv) was added and stirred for an additional 15 min. The solution was cooled to 0 °C and, under a stream of argon, **13 G** (50 mg, 0.14 mmol) was added. After 45 min HCl (2 M, 1.5 mL) was added, and stirred for 20 h. The solvent was evaporated under vacuum and gentle heating, followed by purification via column chromatography (DCM +10 % MeOH +AcOH). The solvent was evaporated under reduced pressure and the residue two times lyophilized (dissolved in ACN/aqueous 1 M HCl 1 : 1, 2 mL) to give product **14 G** in 23 % yield over two steps.

^1^H NMR (600 MHz, CD_3_OD) δ=7.64 (s, 1H), 7.28 (s, 1H), 7.17–7.13 (m, 2H), 6.84–6.79 (m, 2H), 5.14 (br s, 1H), 3.97 (s, 3H), 3.94 (s, 3H), 3.83–3.75 (m, 2H), 3.78 (s, 3H), 3.74 (d, *J*=4.2, 2H), 3.24 (dd, *J*=13.0, 5.9, 1H), 3.10 (dd, *J*=13.0, 9.4, 1H). ^13^C NMR (151 MHz, CD_3_OD) δ=168.62, 160.30, 155.20, 151.35, 144.59, 131.32, 131.11, 123.20, 115.10, 111.61*, 109.64, 58.05*, 57.41, 56.97, 55.69, 47.16, 28.37, 25.20. *Due to peak broadening in 1D ^13^C NMR spectrum, chemical shifts were taken from ^1^H‐^13^C HSQC cross peaks. HRMS (ESI): *m/z* calculated for C_20_H_24_N_2_O_7_Se+H^+^: 485.0821 [*M*+H]^+^; found: 485.0828.

**General procedure for synthesis of selenoesters 17 X**: Hydrazination, loading of the first amino acid and peptide synthesis were performed according to the procedure described in the Supporting Information (section 6.1 and 6.2).

Conversion to the selenoester was performed by dissolving the crude hydrazide peptide in conversion buffer (6 M Gdn‐HCl, 200 mM HEPES, 200 mM TCEP, 50 mM DPDS, pH 1.5) at a concentration of 10 mM. After addition of 30 equiv of acetylacetone, the solution was agitated for 1–2 h, after which remaining DPDS was extracted gently with hexane (ca. 1.5× the volume of reaction solution, 3–5 times). Direct purification (Kromasil 300‐10‐C4 10×250 mm semiprep) gave the selenoesters **17 X** (Supporting Information 6.3).

**Gly(SeAUX) model ligations of peptides 20 X**: Auxiliary peptide **16 G** (0.05 mg, 0.05 μmol, 1 equiv) and selenoester **17 X** (0.08 mg, 0.1 μmol, 2 equiv) were dissolved in ligation buffer (6 M Gdn‐HCl, 200 mM sodium acetate, pH 4.5; degassed with argon) at a concentration of 1.7 mM (30 μL, calculated for the reduced monomer). Then a solution of TCEP (0.15 mM, 0.5 equiv based on TCEP concentration) and DPDS (50 mM) in ligation buffer (agitated beforehand for ca. 15 min) were added. After 5 min 1.5 μL were taken from the reaction solution, diluted with 18.5 μL ligation buffer and analyzed via HPLC and LC–MS. The remaining reaction solution was shaken at 37 °C. For all further time points: 1.5 μL sample were taken, diluted in 10 μL ligation buffer and irradiated with UV light (365 nm, 166 mW/cm^2^) for 3 min, after which 10 μL of 2.5 % hydrazine (in MQ‐H_2_O) were added and samples measured via HPLC (Figure S38–S43) and LC–MS.

For more experimental details and data, please see Supporting Information.

## Supporting Information

Additional references cited within the Supporting Information.[^11^, ^21^, ^26^]

## Conflict of interest

The authors declare no conflict of interest.

1

## Supporting information

As a service to our authors and readers, this journal provides supporting information supplied by the authors. Such materials are peer reviewed and may be re‐organized for online delivery, but are not copy‐edited or typeset. Technical support issues arising from supporting information (other than missing files) should be addressed to the authors.

Supporting Information

## Data Availability

The data that support the findings of this study are available from the corresponding author upon reasonable request.
