# Supplementary material for: Light‐Cleavable Auxiliary for Diselenide–Selenoester Ligations of Peptides and Proteins
Source: Chemistry. 2023 Jul 18;29(46):e202301253. doi: 10.1002/chem.202301253 (PMC10946927; doi:10.1002/chem.202301253)
Supplement: Supplementary file 1 — Supporting Information [file CHEM-29-0-s001.pdf]

Chemistry–A European Journal

Supporting Information

Light-Cleavable Auxiliary for Diselenide–Selenoester Ligations of Peptides and Proteins

Maximilian Schrems, Alexander V. Kravchuk, Gerhard Niederacher, Florian Exler, Claudia Bello, and Christian F. W. Becker*

Contents

1. Materials ... 3
2. Instrumentation ... 3
3. Precursor synthesis ... 4
   3.1. Synthesis of bis(4-methoxybenzyl) diselenide[26a] ... 4
   3.2. L-Alanine tert-butyl ester ... 5
4. Synthesis of Gly(SeAUX) **14G** ... 6
   4.1. *tert*-butyl (1-(3,4-dimethoxyphenyl)-2-hydroxyethyl)glycinate **10G** ... 7
   4.2. *tert*-butyl 2-(4-(3,4-dimethoxyphenyl)-2-oxido-1,2,3-oxathiazolidin-3-yl)acetate **11G** ... 9
   4.3. *tert*-butyl 2-(4-(3,4-dimethoxyphenyl)-2,2-dioxido-1,2,3-oxathiazolidin-3-yl)acetate **12G** ... 11
   4.4. 2-(4-(4,5-dimethoxy-2-nitrophenyl)-2,2-dioxido-1,2,3-oxathiazolidin-3-yl)acetic acid **13G** ... 13
   4.5. (1-(4,5-dimethoxy-2-nitrophenyl)-2-((4-methoxybenzyl)selanyl)ethyl)glycine **14G** ... 15
5. Synthesis of Ala(SeAUX) **14A** ... 18
   5.1. tert-butyl (1-(3,4-dimethoxyphenyl)-2-hydroxyethyl)-L-alaninate **10A** ... 19
   5.2. *tert*-butyl (2S)-2-(4-(3,4-dimethoxyphenyl)-2-oxido-1,2,3-oxathiazolidin-3-yl)propanoate **11A** ... 22
   5.3. *tert*-butyl (2S)-2-(4-(3,4-dimethoxyphenyl)-2,2-dioxido-1,2,3-oxathiazolidin-3-yl)propanoate **12A** ... 25
   5.4. (2S)-2-(4-(4,5-dimethoxy-2-nitrophenyl)-2,2-dioxido-1,2,3-oxathiazolidin-3-yl)propanoic acid **13A** ... 28
   5.5. (1-(4,5-dimethoxy-2-nitrophenyl)-2-((4-methoxybenzyl)selanyl)ethyl)alanine **14A** ... 31
6. Peptide synthesis ... 35
   6.1. General procedure solid phase peptide synthesis ... 35
   6.2. General procedure resin hydrazine loading ... 35
   6.3. LYRAX-SePh **17X** synthesis and analysis ... 36
   6.4. Gly(SeAUX)-peptide **16G** synthesis ... 40
   6.5. Ala(SeAUX)-peptide **16A** synthesis ... 40
   6.6. Gly(SeAUX)-G-CSF 126-174 peptide **29** synthesis ... 41
   6.7. Synthesis of peptides aGVTSWA **Da-25** and AGVTSWA **25** ... 42
7. G-CSF selenoester synthesis ... 43
   7.1. Expression and purification of G-CSF 1-124-$NHNH_2$ **27** ... 43
   7.2. Conversion of G-CSF 1-124 hydrazide **27** to selenoester **28** ... 44
8. Native chemical ligation ... 46
   8.1. pH variation ligation ... 46

8.2. Gly(SeAUX) model ligation peptides **20X** ... 47
8.3. Ala(SeAUX) model ligation peptides **24X** ... 50
8.4. Gly(SeAUX) model ligation without TCEP peptides **20X** ... 53
8.5. Expressed protein selenoester ligation, G-CSF 1-124 selenoester **28** and Gly(SeAUX)-G-CSF 126-174 **29** ... 60
9. Racemization experiments Ala(SeAUX)-peptide **16A** ... 62
10. Abbreviations ... 62
11. References ... 63

1. Materials

All chemicals purchased from commercial vendors were used without further purification. Anhydrous solvents were acquired from commercial vendors and used as is. Preparative flash column chromatography for the purification of compounds was performed using Silica 60 (0.04-0.063 mm), Macherey-Nagel. For thin layer chromatography, pre-coated TLC sheets ALUGRAM Xtra SIL G/UV_{254} (0.2 mm silica gel 60), Macherey-Nagel were used.

2. Instrumentation

Analytical RP-HPLC analysis was performed on a Dionex Ultimate 3000 or Vanquish HPLC system, using a Macherey-Nagel Nucleodur 300-5 C4ec 4.6x150 mm column and the gradient indicated.

LC-MS measurements were performed either on a:

Waters Auto Purification HPLC/MS system (3100 Mass Detector, 2545 Binary Gradient Module, 2767 Sample Manager and 2489 UV/Visible Detector) by electrospray ionization (ESI), operating in positive ion mode, equipped with a Kromasil 300-5-C4 or 300-5-C18 column (50 × 4.6 mm, 5 µm particle size) at a flow rate of 1 mL/min running a linear gradient from 5-65% or 5-90 % of buffer B (ACN + 0.05% TFA) in buffer A (MQ-H_2O + 0.05% TFA) over 10 min.

Waters SQD2 and Arc HPLC-System by electrospray ionization (ESI), operating in positive ion mode, equipped with a Waters XBridge C4 column, 3.5 µm, 300A, 2.1x100 mm at a flow rate of 0.5 mL/min running a linear gradient of 5-65% of buffer B (ACN + 0.05% TFA) in buffer A (MQ-H_2O + 0.05% TFA) over 7 min.

Dionex Ultimate 3000 HPLC system, coupled to Thermo Scientific MSQ Plus single quadrupole by electrospray ionization (ESI), operating in positive ion mode, equipped with a Waters X-select CSH C18 XP (3 mm x 75 mm x 2.5 µm) column, running a linear gradient from 1-61% or 1-90% of buffer B (ACN + 0.08% formic acid) in buffer A (MQ-H_2O + 0.1% formic acid) in 6.5 min.

Preparative and semipreparative HPLC was performed either on a:

Waters Prep 150 LC System, equipped with a Kromasil 300-10-C4, 21.2x250 mm prep or Kromasil 300-10-C4 10x250 mm semiprep column, running a gradient as indicated.

Varian ProStar RP-HPLC system, equipped with a Kromasil 300-10-C4, 21.2x250 mm prep or Kromasil 300-10-C4 10x250 mm semiprep column, running a gradient as indicated. This system was used in combination with a Timberline TL105 HPLC column heater for purifications at 60 °C.

Small molecule High Resolution MS data was obtained on a Bruker maXis UHR-TOF instrument, using electrospray ionization in positive ion mode.

Protein High Resolution MS data was obtained with an LTQ Orbitrap Velos system attached to a Dionex Ultimate 3000 Nano instrument by electrospray ionization in positive ion mode with buffer A (MQ-H_2O + 0.1% formic acid) and buffer B (80% ACN in MQ-H_2O + 0.1% formic acid) as eluents.

NMR spectra were measured on a Bruker AV III 600 or Bruker AV II HD 700 instrument using the residual protonated solvent signal as reference.

Photocleavage was performed using a Thor-Labs CS2010 UV lamp at 365 nm and 166 mW/cm^2 for the indicated time.

3. Precursor synthesis

3.1. Synthesis of bis(4-methoxybenzyl) diselenide[26a]

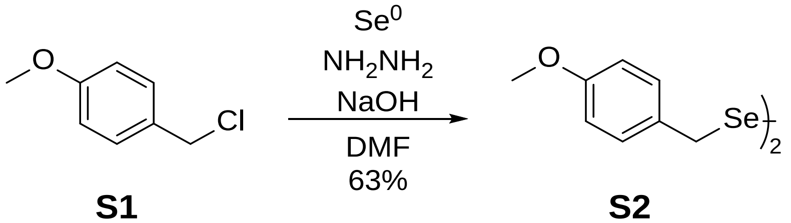

Scheme S1 Synthesis of diselenide **S2**

Selenium powder (1 g, 12.7 mmol) and NaOH (0.76 g, 19 mmol, 1.5 equiv.) were mixed under argon atmosphere in anhydrous DMF (6.5 mL). Hydrazine (64% in H_2O, 0.675 mL, 0.696 g, 13.9 mmol, 1.1 equiv.) was added dropwise to the stirred suspension, resulting in gas evolution and a dark brown solution. After 6 h 4-methoxybenzyl chloride **S1** (0.99 g, 6.3 mmol, 0.5 equiv.) was added, and stirred for another 45 minutes, after which the reaction was quenched with MQ-H_2O, and the solution extracted with DCM. The organic phases were washed with 6 M HCl, MQ-H_2O and brine and dried over $MgSO_4$. Purification via column chromatography (petroleum ether/ethyl acetate 9:1) gave **S2** in 0.8 g (2 mmol, 63%) yield, forming bright yellow crystals. Analysis is in agreement with the literature.[26b]

^{1}H NMR (700 MHz, $CDCl_3$) δ = 7.16 (d, *J*=8.7, 4H), 6.84 (d, *J*=8.7, 4H), 3.84 (s, 4H), 3.80 (s, 6H).

^{13}C NMR (176 MHz, $CDCl_3$) δ = 158.76, 131.08, 130.09, 113.84, 55.27, 32.22

^{77}Se NMR (114 MHz, $CDCl_3$) δ = 396.72.

HRMS (ESI): *m/z* calculated for $C_{16}H_{18}O_2Se_2$+Na^+: 424.9529 [*M*+Na]$^+$; found: 424.9525

3.2. L-Alanine tert-butyl ester

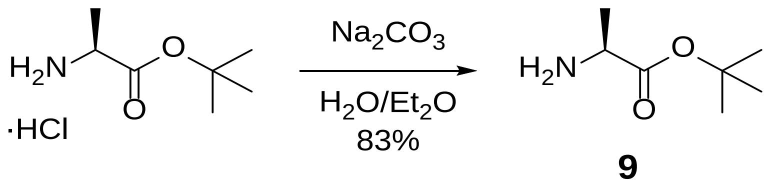

Scheme S 2 Conversion of L-alanine tert-butyl ester into free amine **9**

L-Alanine hydrochloride (1 g, 5.5 mmol, 1 equiv.) and disodium carbonate (2.9 g, 27.5 mmol, 5 equiv.) were dissolved in 33 mL MQ-H_2O. Diethyl ether (33 mL) was added, and the biphasic mixture was stirred at r.t. for 2.5 h. The solution was then extracted eight times with diethyl ether. The combined organic layers were dried over Na_2SO_4 and the solvent evaporated under reduced pressure. The crude was purified further via vacuum distillation to give the product **9** in 83% yield (665 mg) as a clear liquid. Analysis is in agreement with the literature.[26c]

^{1}H NMR (700 MHz, $CDCl_3$) δ = 3.43 (q, *J*=7.0, 1H), 1.45 (s, 9H), 1.30 (d, *J*=7.0, 3H).

^{13}C NMR (176 MHz, $CDCl_3$) δ = 175.70, 80.91, 50.55, 27.99, 20.58.

HRMS (ESI): *m/z* calculated for $C_7H_{15}NO_2$+H^+: 146.1176 [*M*+H]$^+$; found: 146.1176

4. Synthesis of Gly(SeAUX) **14G**

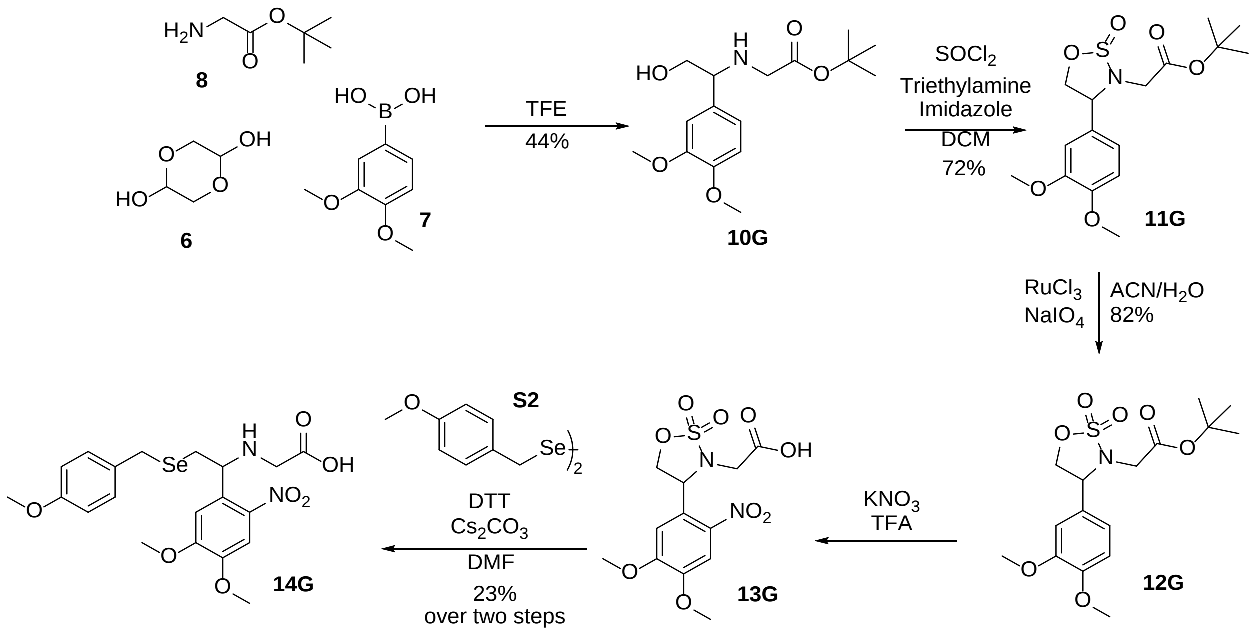

Scheme S 3 Synthesis scheme of Gly(SeAUX) **14G**

4.1. *tert*-butyl (1-(3,4-dimethoxyphenyl)-2-hydroxyethyl)glycinate **10G**

Detailed reaction conditions are reported in the experimental section of the main manuscript.

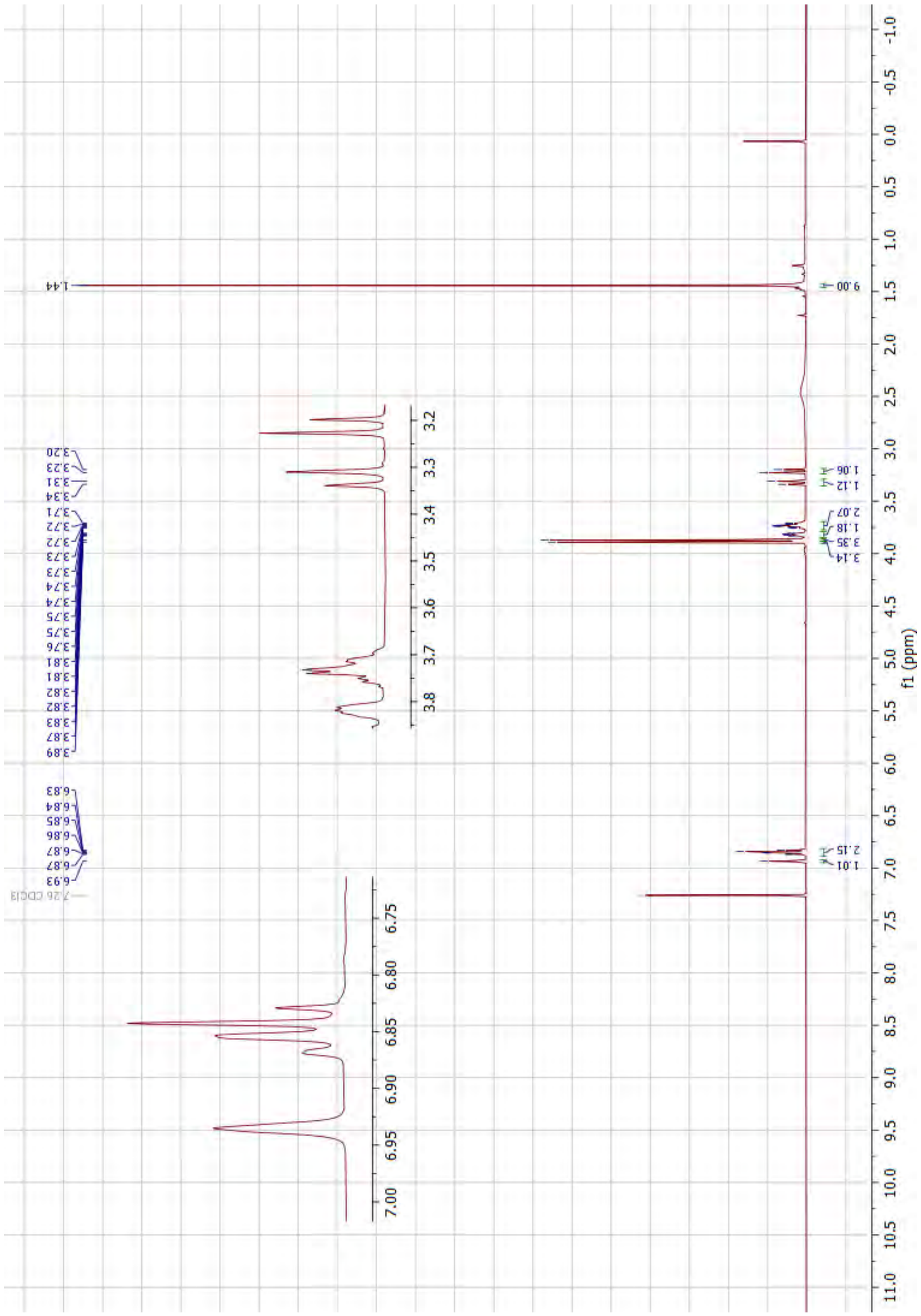

Figure S 1 ^{1}H NMR (600 MHz) of **10G** in $CDCl_3$

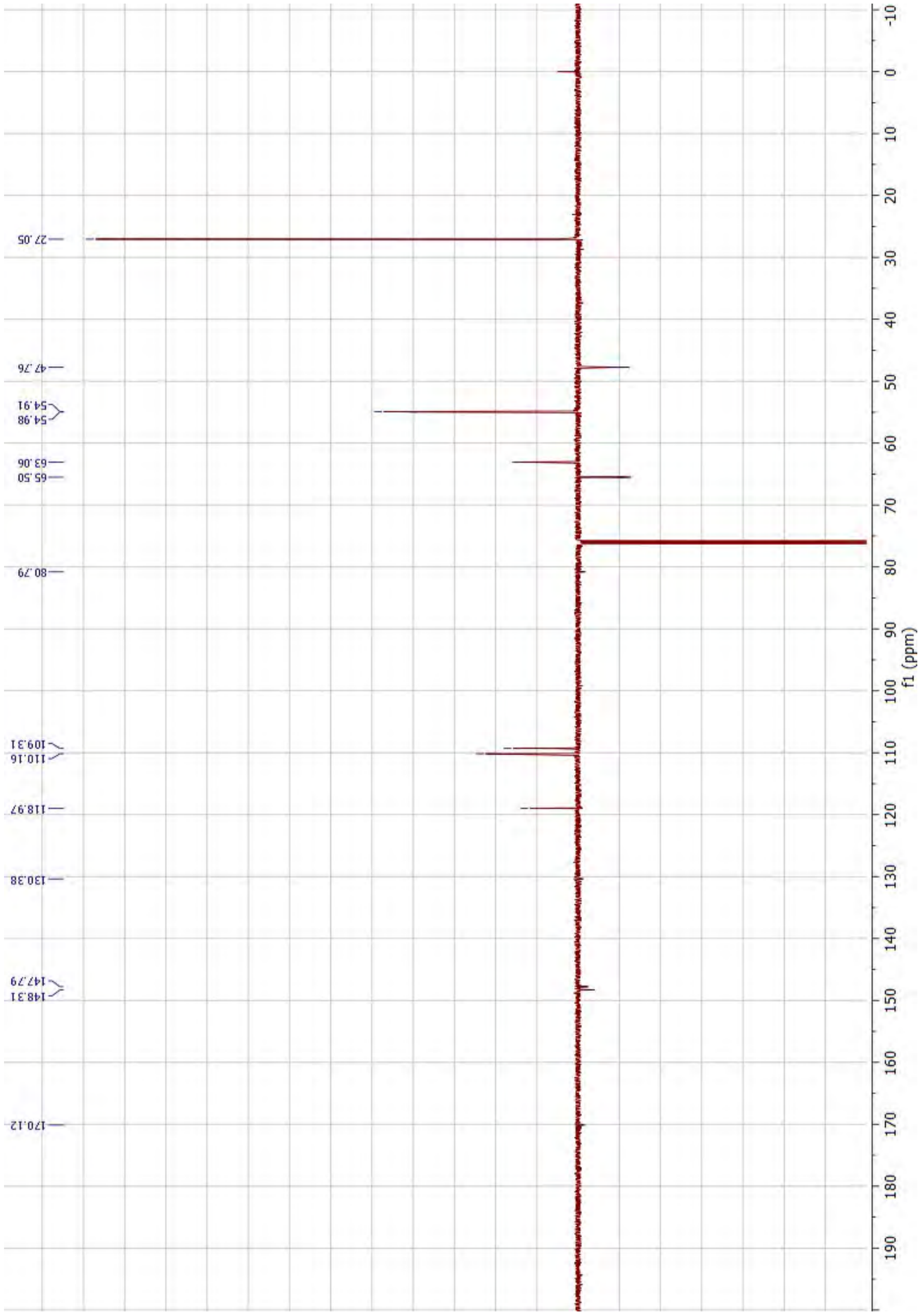

Figure S 2 ^{13}C NMR (151 MHz) of **10G** in $CDCl_3$

4.2. *tert*-butyl 2-(4-(3,4-dimethoxyphenyl)-2-oxido-1,2,3-oxathiazolidin-3-yl)acetate **11G**

Detailed reaction conditions are reported in the experimental section of the main manuscript.

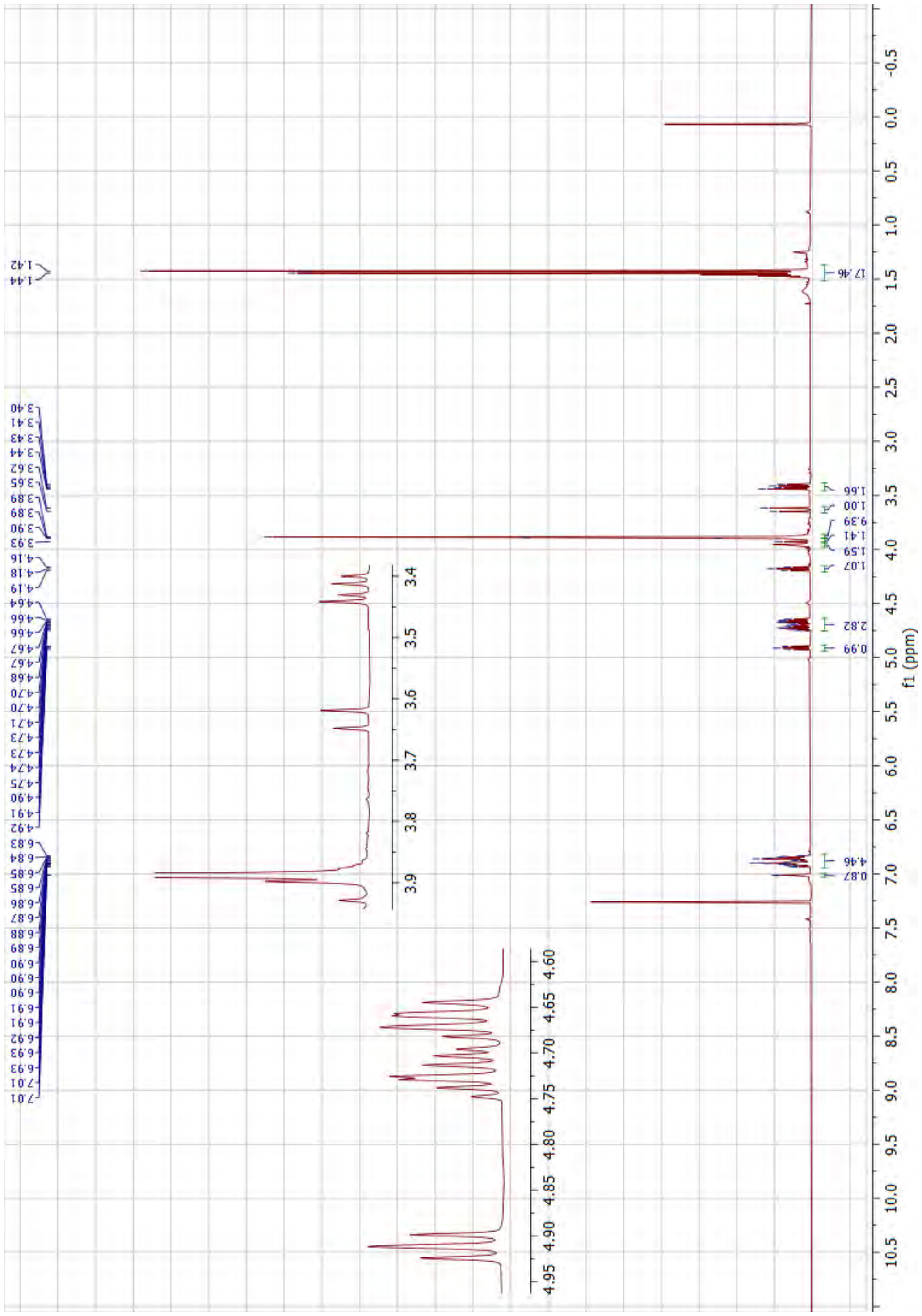

Figure S 3 ^{1}H NMR (600 MHz) of **11G** in $CDCl_3$

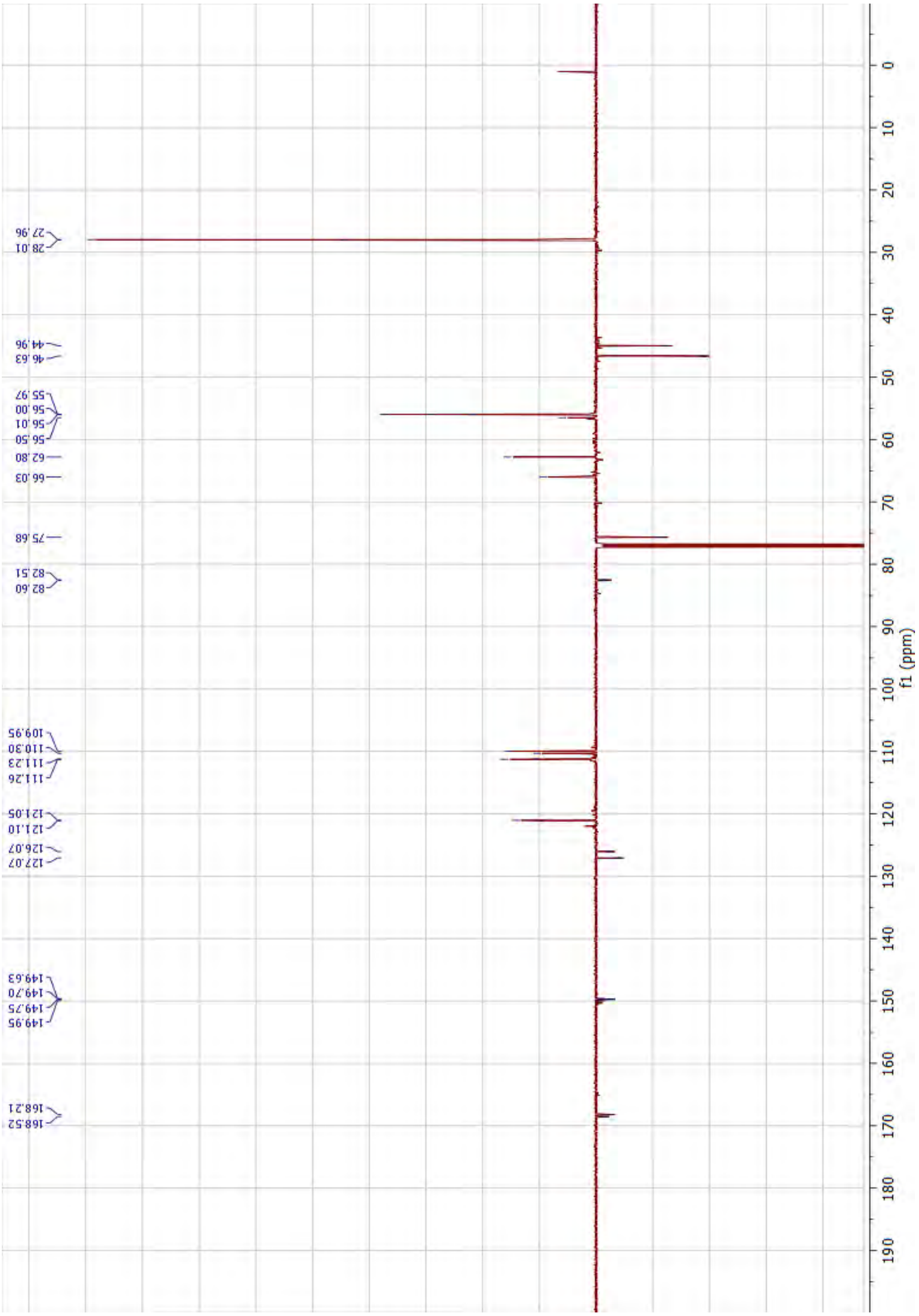

Figure S 4 ^{13}C NMR (151 MHz) of **11G** in $CDCl_3$

4.3. *tert*-butyl 2-(4-(3,4-dimethoxyphenyl)-2,2-dioxido-1,2,3-oxathiazolidin-3-yl)acetate **12G**

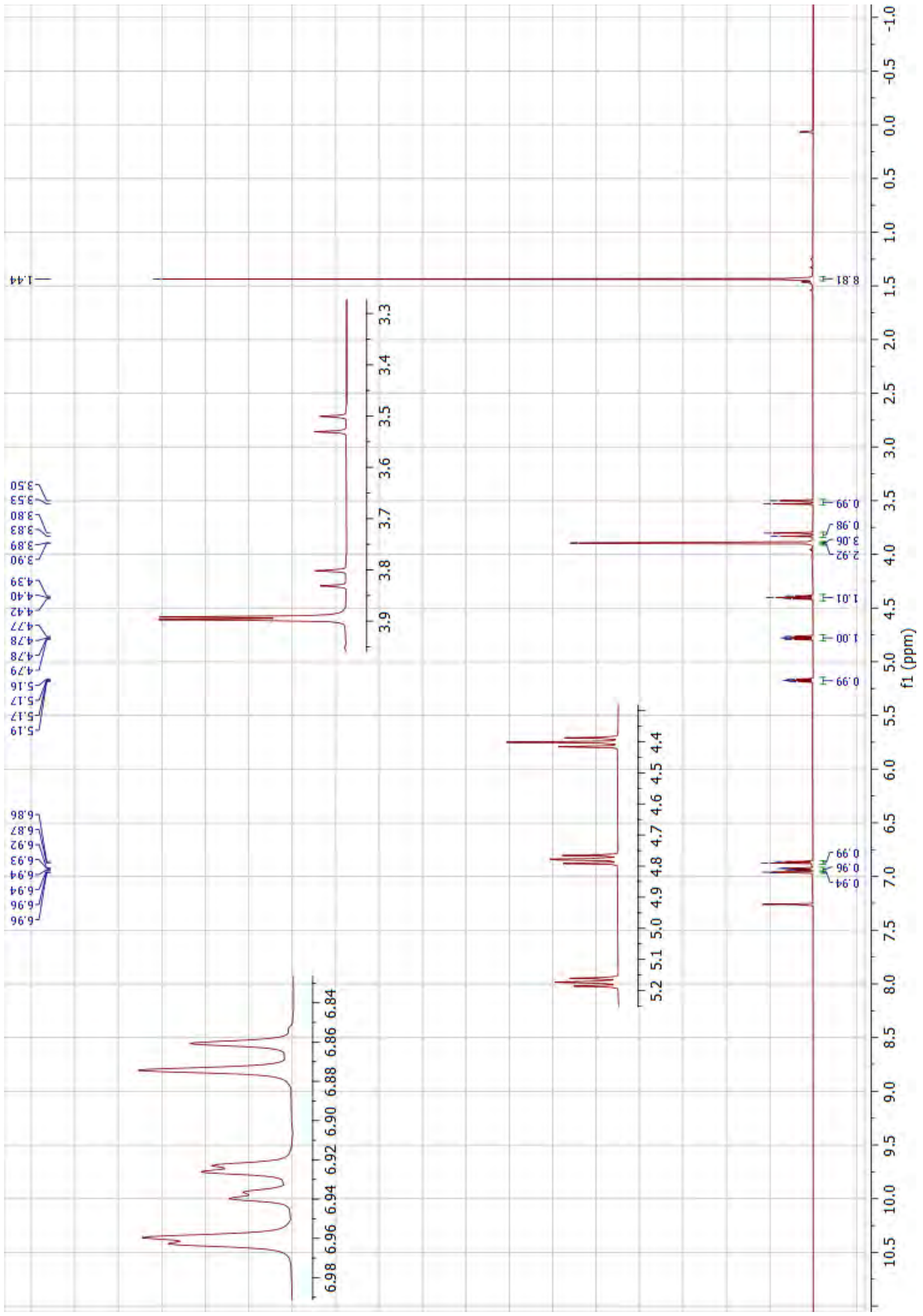

Figure S 5 ^{1}H NMR (600 MHz) of **12G** in $CDCl_3$

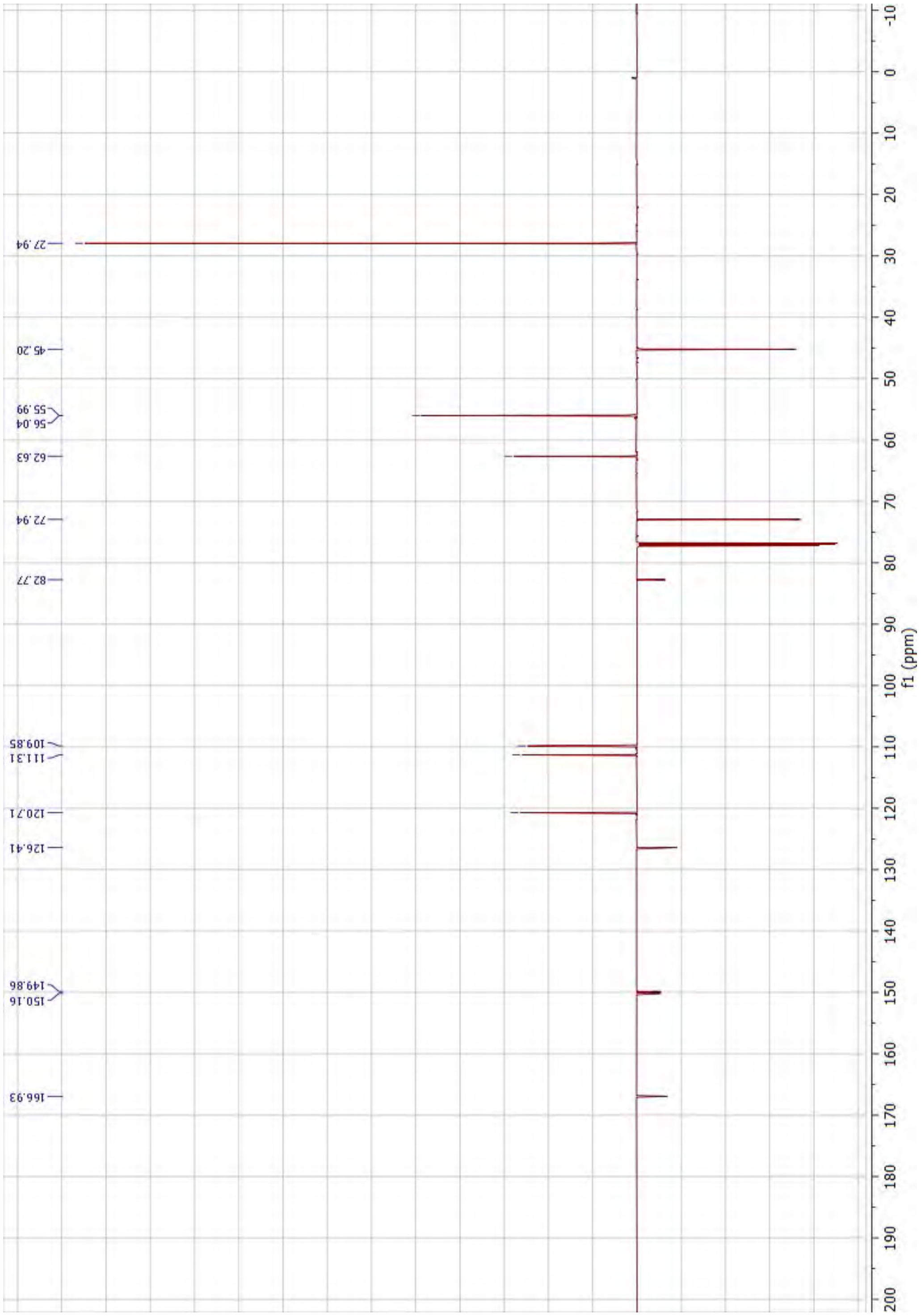

Figure S 6 ^{13}C NMR (151 MHz) of **12G** in $CDCl_3$

4.4. 2-(4-(4,5-dimethoxy-2-nitrophenyl)-2,2-dioxido-1,2,3-oxathiazolidin-3-yl)acetic acid **13G**

Detailed reaction conditions are reported in the experimental section of the main manuscript.

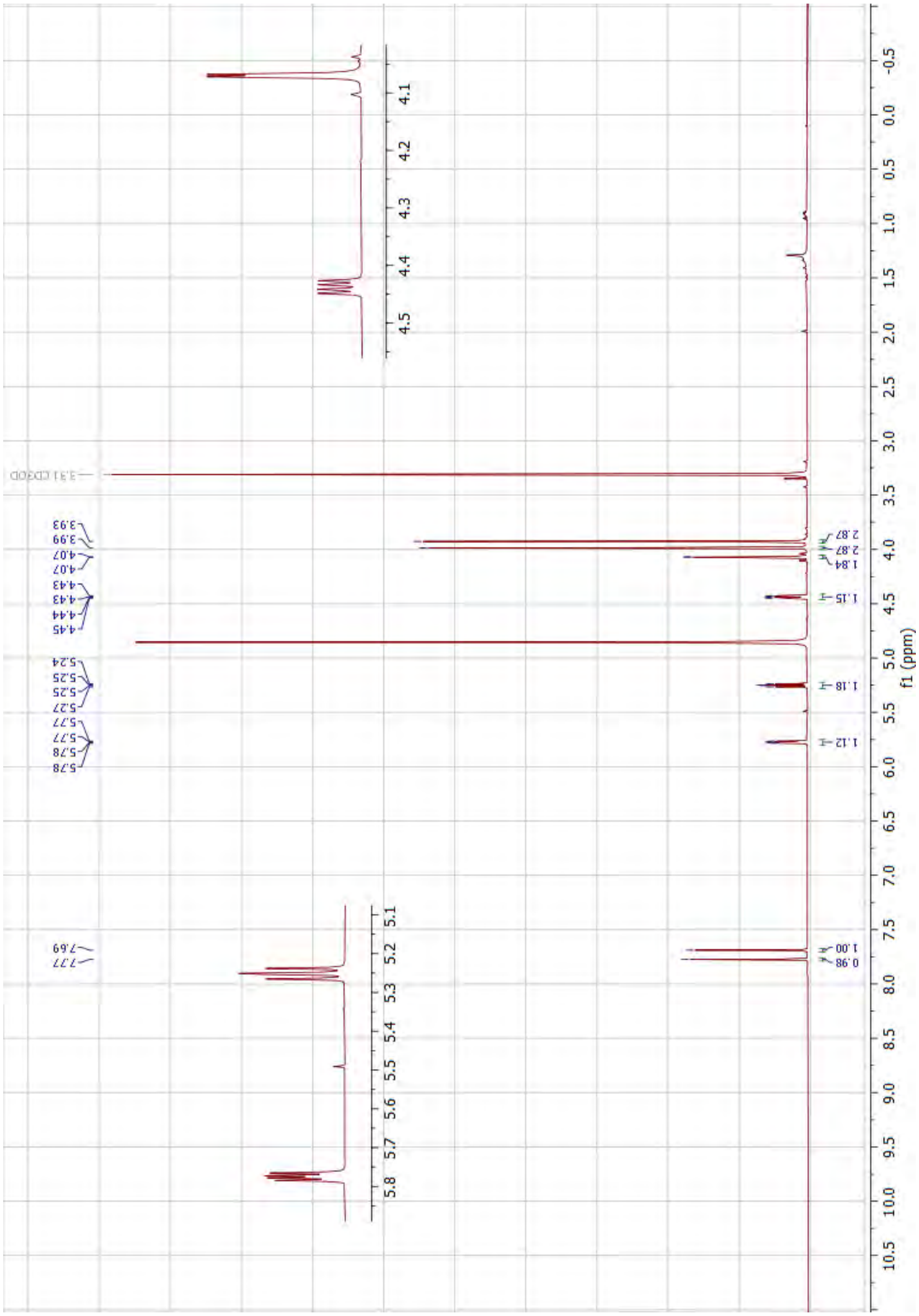

Figure S 7 ^{1}H NMR (600 MHz) of **13G** in CD_3OD

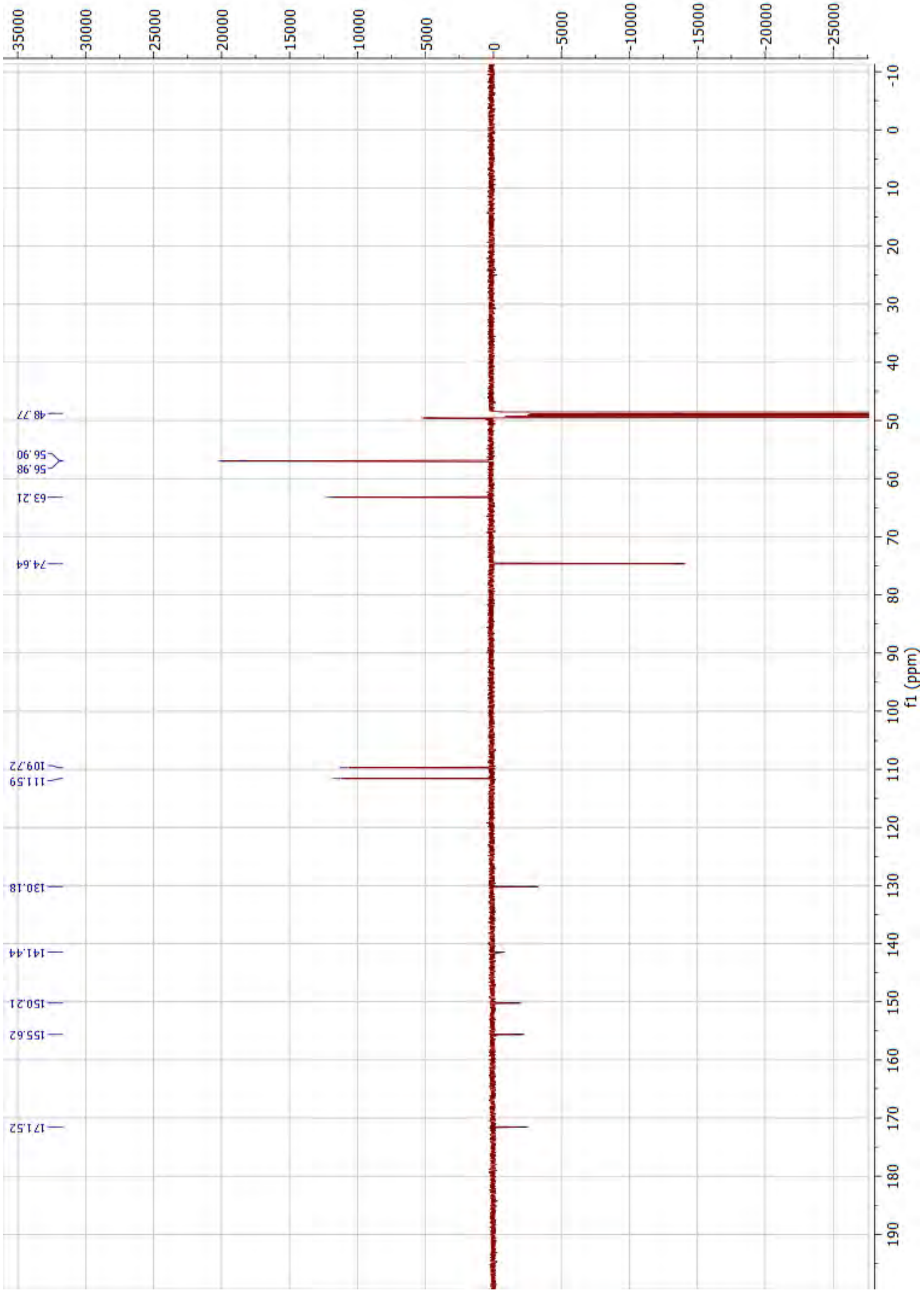

Figure S 8 ^{13}C NMR (151 MHz) of **13G** in CD_3OD

4.5. (1-(4,5-dimethoxy-2-nitrophenyl)-2-((4-methoxybenzyl)selanyl)ethyl)glycine **14G**

Detailed reaction conditions are reported in the experimental section of the main manuscript.

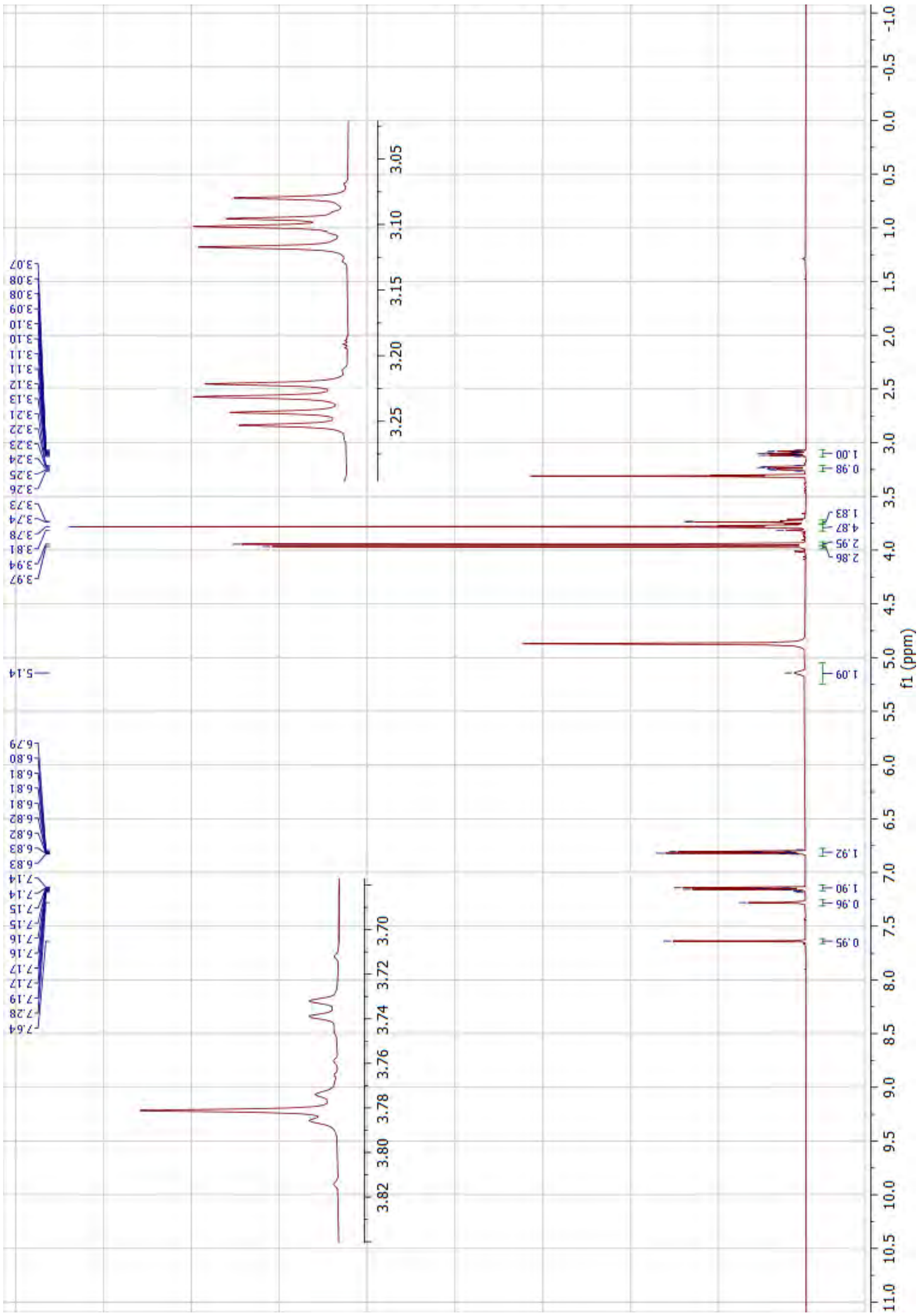

Figure S 9 ^{1}H NMR (600 MHz) of **14G** in CD_3OD

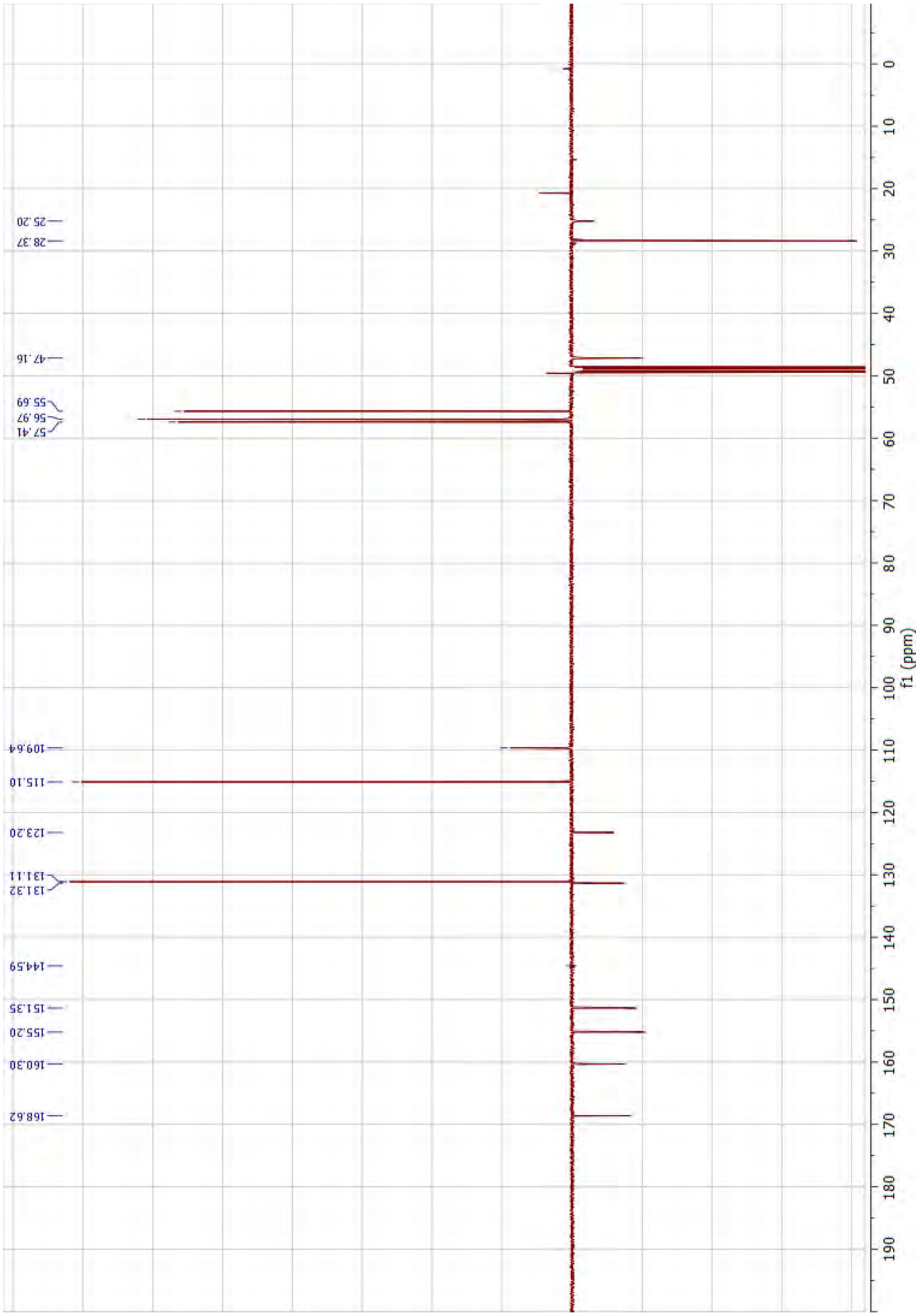

Figure S 10 ^{13}C NMR (151 MHz) of **14G** in CD_3OD

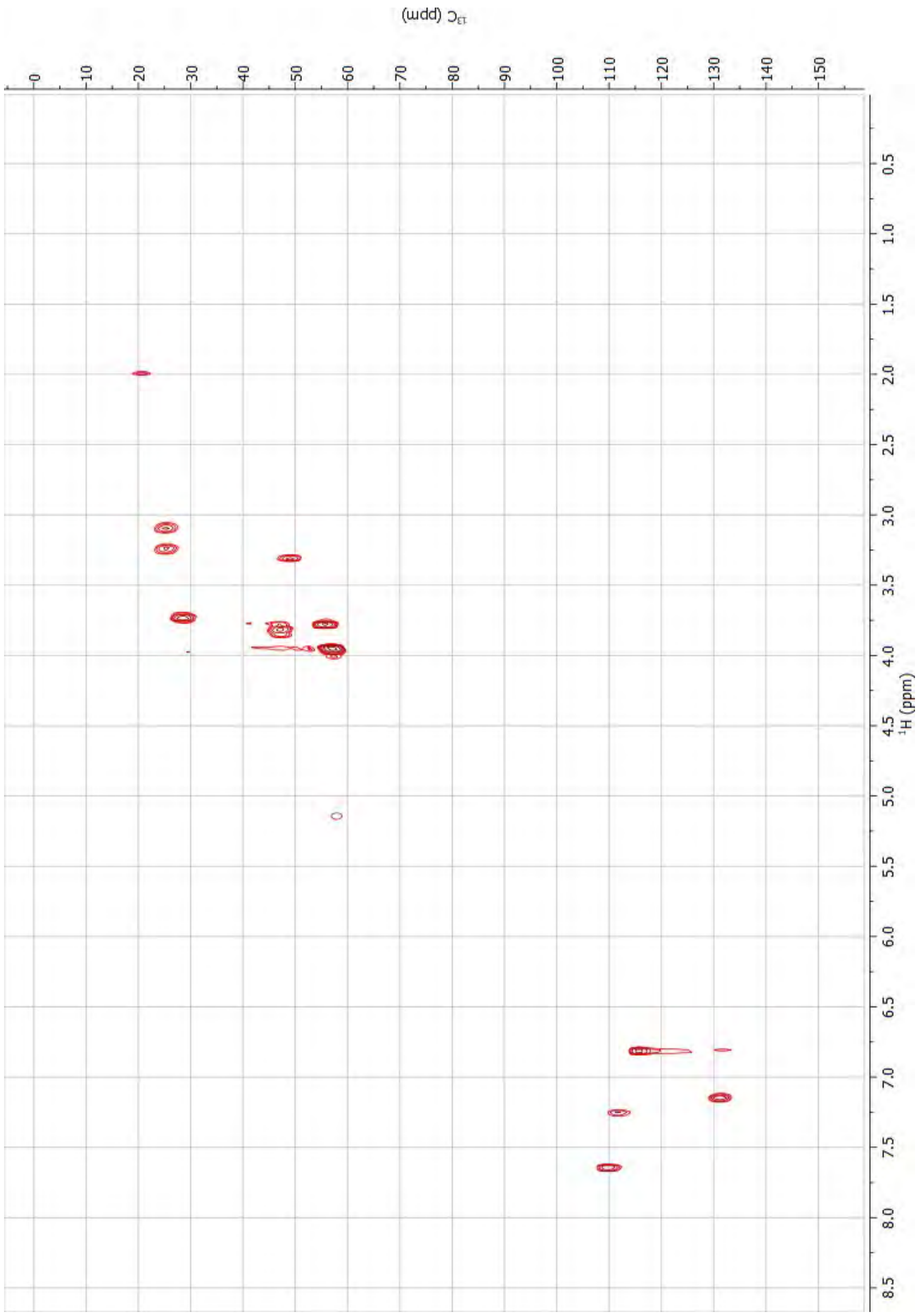

Figure S 11 ^{1}H-^{13}C HSQC spectrum of **14G** in CD_3OD

5. Synthesis of Ala(SeAUX) **14A**

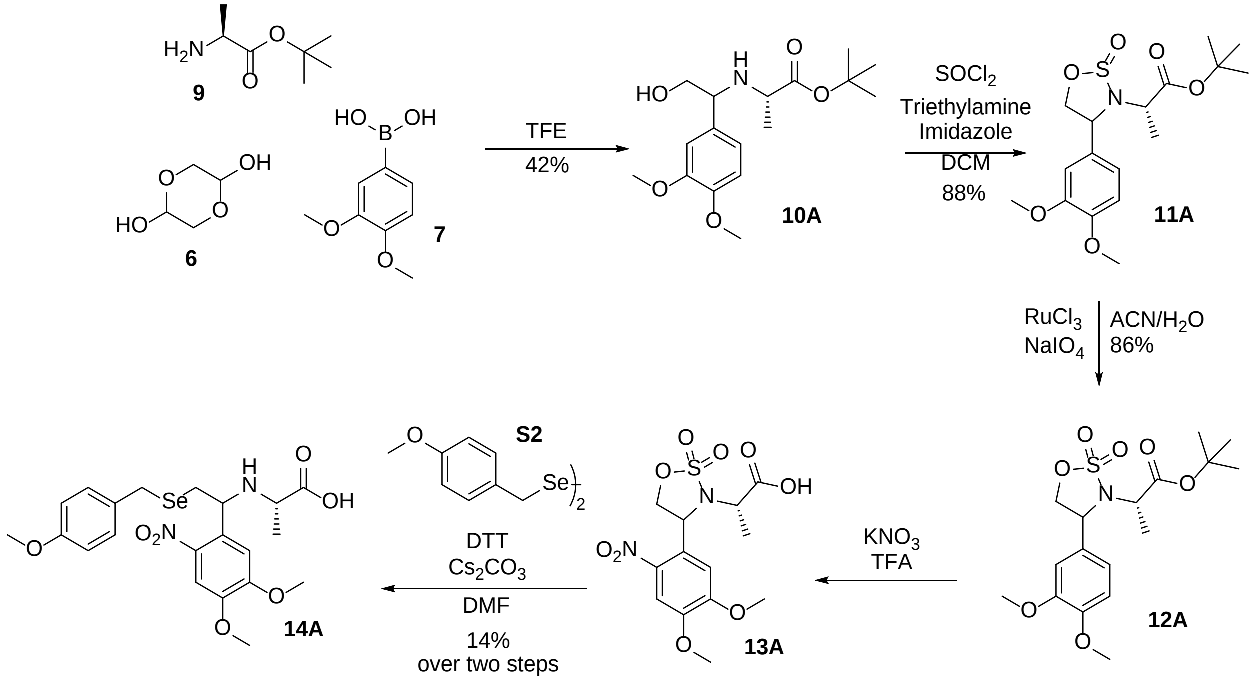

Scheme S 4 Synthesis scheme of Gly(SeAUX) **14A**

5.1. tert-butyl (1-(3,4-dimethoxyphenyl)-2-hydroxyethyl)-L-alaninate **10A**

1,4-dioxane-2,5-diol **6** (glycolaldehyde dimer; 66 mg, 0.55 mmol, 0.5 equiv.) and (3,4-dimethoxyphenyl) boronic acid **7** (200 mg, 1.1 mmol, 1 equiv.) were suspended in 2,2,2-trifluorethanol (2 mL). After addition of L-alanine *tert*-butyl ester **9** (167 µL, 160 mg, 1.1 mmol, 1 equiv.) the solution was stirred for 48 h at r.t. The solvent was then evaporated under reduced pressure and ethyl acetate was added. The organic phase was washed with 1 M NaOH (3x), followed by H_2O (2x), brine (1x) and dried over Na_2SO_4. Evaporation of the solvent under reduced pressure gave the crude product, which was then purified by column chromatography (silica gel neutralized using triethylamine) with DCM +5% MeOH, giving pure **10A** in 42% yield (150 mg, 0.46 mmol). Diastereomeric ratio 52:48, determined by integration of the amino acid α-proton (at 3.62-3.54 ppm and 3.25 ppm).

^{1}H NMR (600 MHz, $CDCl_3$, major diastereomer) δ = 6.96 (d, 1H), 6.87 – 6.81 (m, 2H), 3.89 (s, 3H), 3.87 (s, 3H), 3.77 (dd, *J*=8.5, 4.3, 1H), 3.72 – 3.67 (m, 1H), 3.65 (dd, *J*=10.9, 8.5, 1H), 3.13 (q, *J*=7.1, 1H), 1.46 (s, 9H), 1.28 (d, *J*=7.1, 3H),

^{1}H NMR (600 MHz, $CDCl_3$, minor diastereomer) δ = 6.87 – 6.81 (m, 3H), 3.88 (s, 3H), 3.86 (s, 3H), 3.74 (dd, *J*=7.8, 4.8, 1H), 3.72 – 3.67 (m, 1H), 3.58 (dd, *J*=10.8, 7.8, 1H), 3.25 (q, *J*=6.9, 1H), 1.38 (s, 9H), 1.28 (d, *J*=6.9, 3H).

^{13}C NMR (151 MHz, $CDCl_3$) δ = 174.50, 149.26, 149.17, 148.73, 148.61, 132.73 (minor), 131.73 (major), 120.11, 119.59, 111.21, 111.11, 110.39 (major), 110.20 (minor), 81.45 (major), 81.27 (minor), 67.13 (major), 66.02 (minor), 63.25 (major), 62.29 (minor), 55.99, 55.94, 55.91, 54.67 (minor), 54.46 (major), 28.08 (major), 27.93 (minor), 19.46 (major), 18.91 (minor).

HRMS (ESI): *m/z* calculated for $C_{17}H_{27}NO_5$+H^+: 326.1962 [*M*+H]$^+$; found: 326.1962

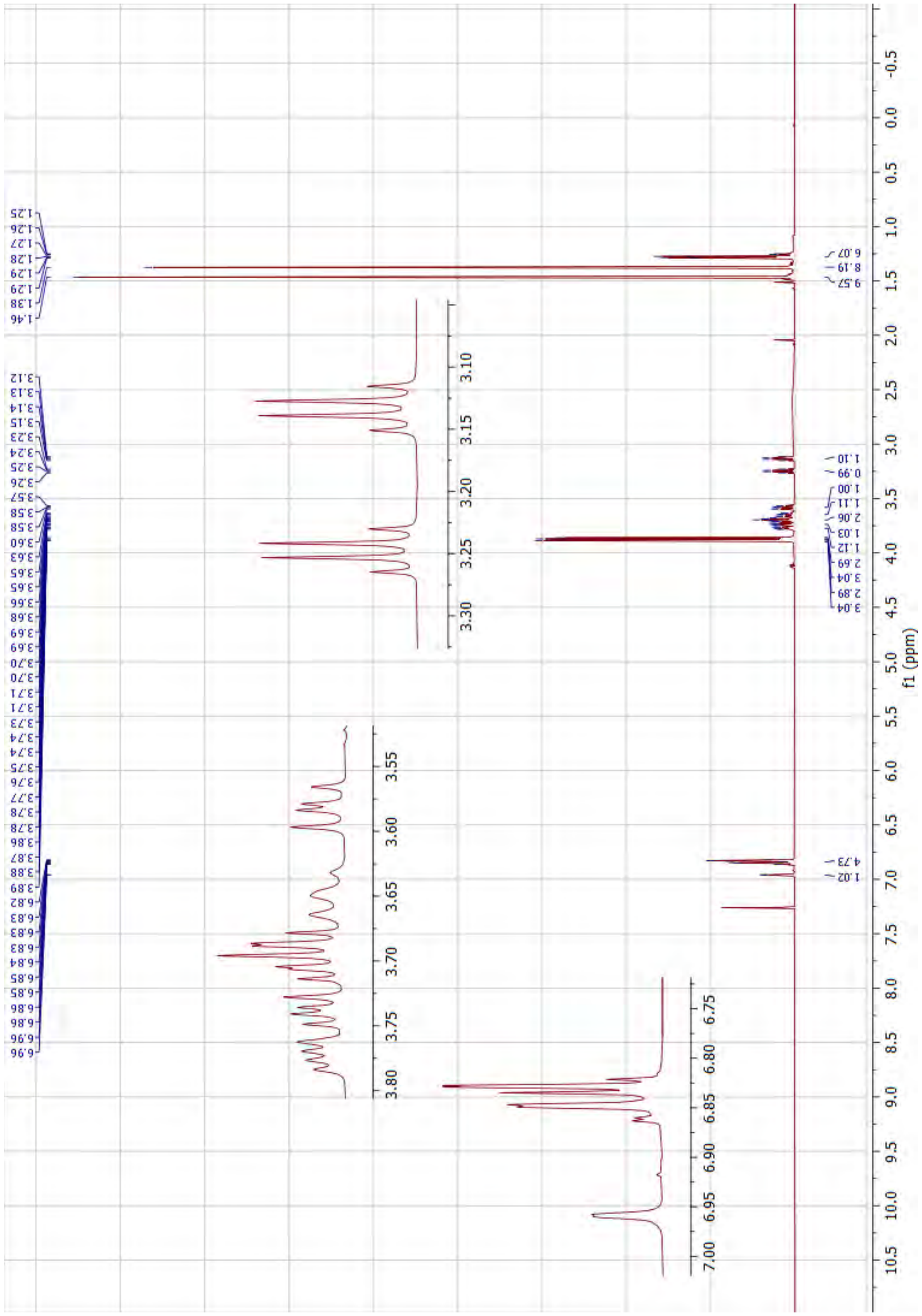

Figure S 12 ^{1}H NMR (600 MHz) of **10A** in $CDCl_3$

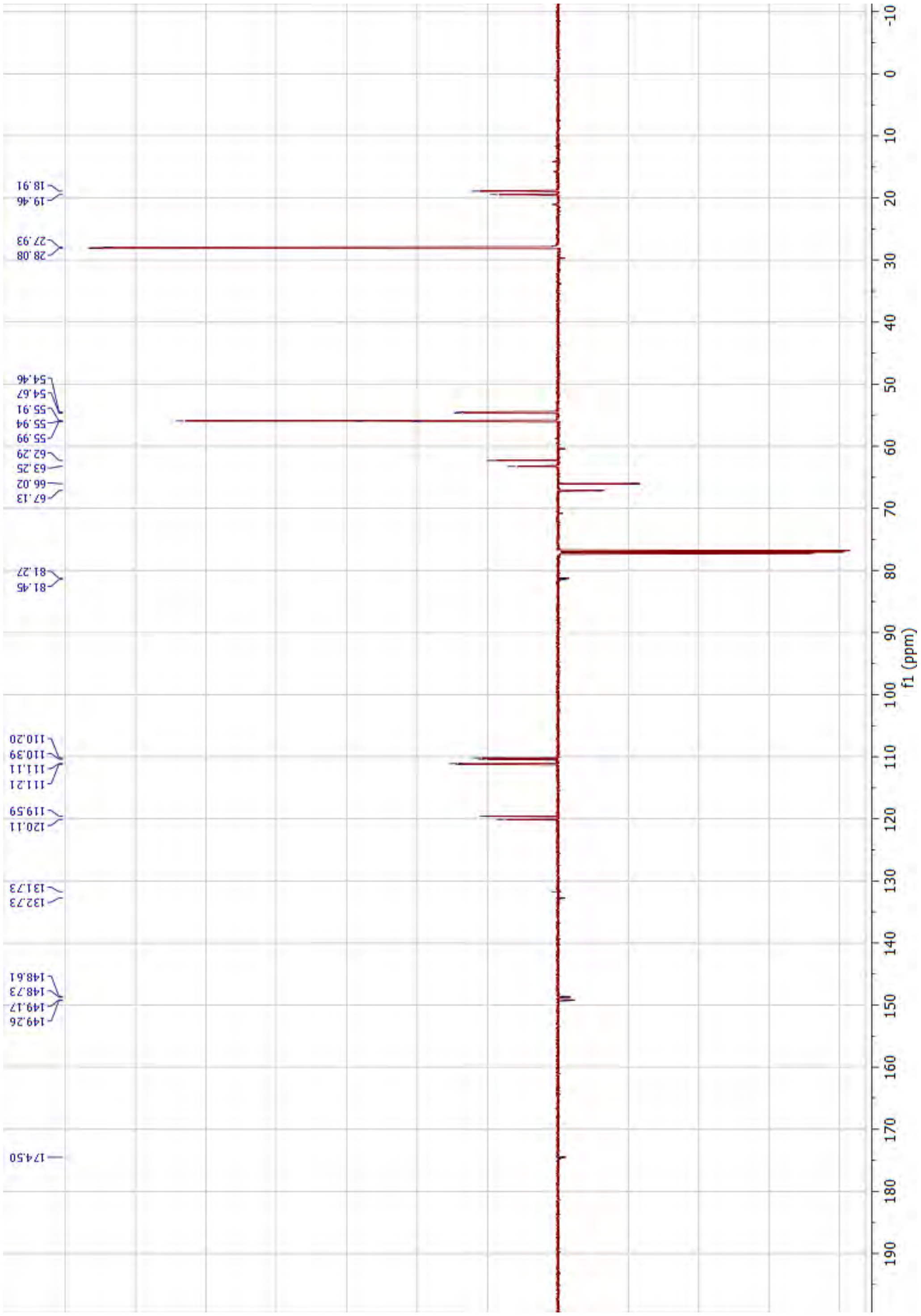

Figure S 13 ^{13}C NMR (151 MHz) of **10A** in $CDCl_3$

5.2. *tert*-butyl (2S)-2-(4-(3,4-dimethoxyphenyl)-2-oxido-1,2,3-oxathiazolidin-3-yl)propanoate **11A**

Starting material **10A** (140 mg, 0.43 mmol, 1 equiv.) and imidazole (123 mg, 1.81 mmol, 4.2 equiv.) were dissolved in anhydrous DCM (1.3 mL), triethylamine (120 µL, 87 mg, 0.86 mmol, 2 equiv.) was added and the solution was cooled down to 0 °C on ice. After addition of thionylchloride (47 µL, 77 mg, 0.645 mmol, 1.5 equiv.) the solution was stirred at 0 °C for 5 min and then at r.t. for 20 h. The reaction was quenched via addition of MQ-H_2O and the organic phase separated, washed with brine and dried over $MgSO_4$. After evaporation of the solvent under reduced pressure and a short silica filtration (petroleum ether/ethyl acetate 1:1, dry loaded) product **11A** was obtained in 88% yield (140 mg) as a mixture of four diastereomers in a ratio of a:b:c:d 12:24:28:36, determined by integration of the benzylic proton (5.06, 5.12, 4.57 and 4.43 ppm). Due to signal overlap, unambiguous assignment was not possible.

^{1}H NMR (600 MHz, $CDCl_3$) δ = 7.13 (d, *J*=2.0, 1H), 7.02 (d, *J*=2.0, 1H), 6.97 – 6.90 (m, 2H), 6.89 – 6.78 (m, 8H), 5.12 (dd, *J*=6.9, 4.5, 1H), 5.09 – 5.02 (m, 2H), 4.79 (dd, *J*=9.1, 7.2, 1H), 4.67 (dd, *J*=11.3, 8.4, 4H), 4.57 (dd, *J*=9.8, 7.2, 1H), 4.43 (dd, *J*=9.6, 7.8, 1H), 4.26 – 4.20 (m, 1H), 4.13 (q, *J*=7.3, 1H), 3.90 (s, 3H), 3.89 (s, 3H), 3.88 (d, *J*=2.0, 3H), 3.87 (s, 3H), 3.87 – 3.81 (m, 1H), 3.66 (q, *J*=7.4, 1H), 3.58 (q, *J*=7.5, 1H), 1.61 (d, *J*=7.6, 3H), 1.53 (d, 3H), 1.48 (s, 9H), 1.47 (s, 9H), 1.44 (s, 9H), 1.43 (s, 9H), 1.37 (d, *J*=7.3, 3H), 1.20 (d, *J*=7.3, 3H).

^{13}C NMR (151 MHz, $CDCl_3$) δ = 172.05, 171.67, 170.97, 149.71, 149.60, 149.57, 149.49, 149.18, 149.16, 131.14, 129.93, 129.48, 126.59, 120.76, 120.34, 120.04, 119.79, 111.24, 111.11, 110.96, 110.16, 110.02, 109.98, 109.59, 82.53, 82.40, 81.93, 76.96, 76.71, 76.25, 75.14, 67.29, 64.98, 61.12, 60.30, 56.02, 55.96, 55.93, 55.92, 55.44, 54.59, 53.97, 52.76, 28.01, 27.96, 27.92, 27.87, 19.04, 17.42, 16.98.

HRMS (ESI): *m/z* calculated for $C_{17}H_{25}NO_6S+Na^+$: 394.1295 $[M+Na]^+$; found: 394.1282

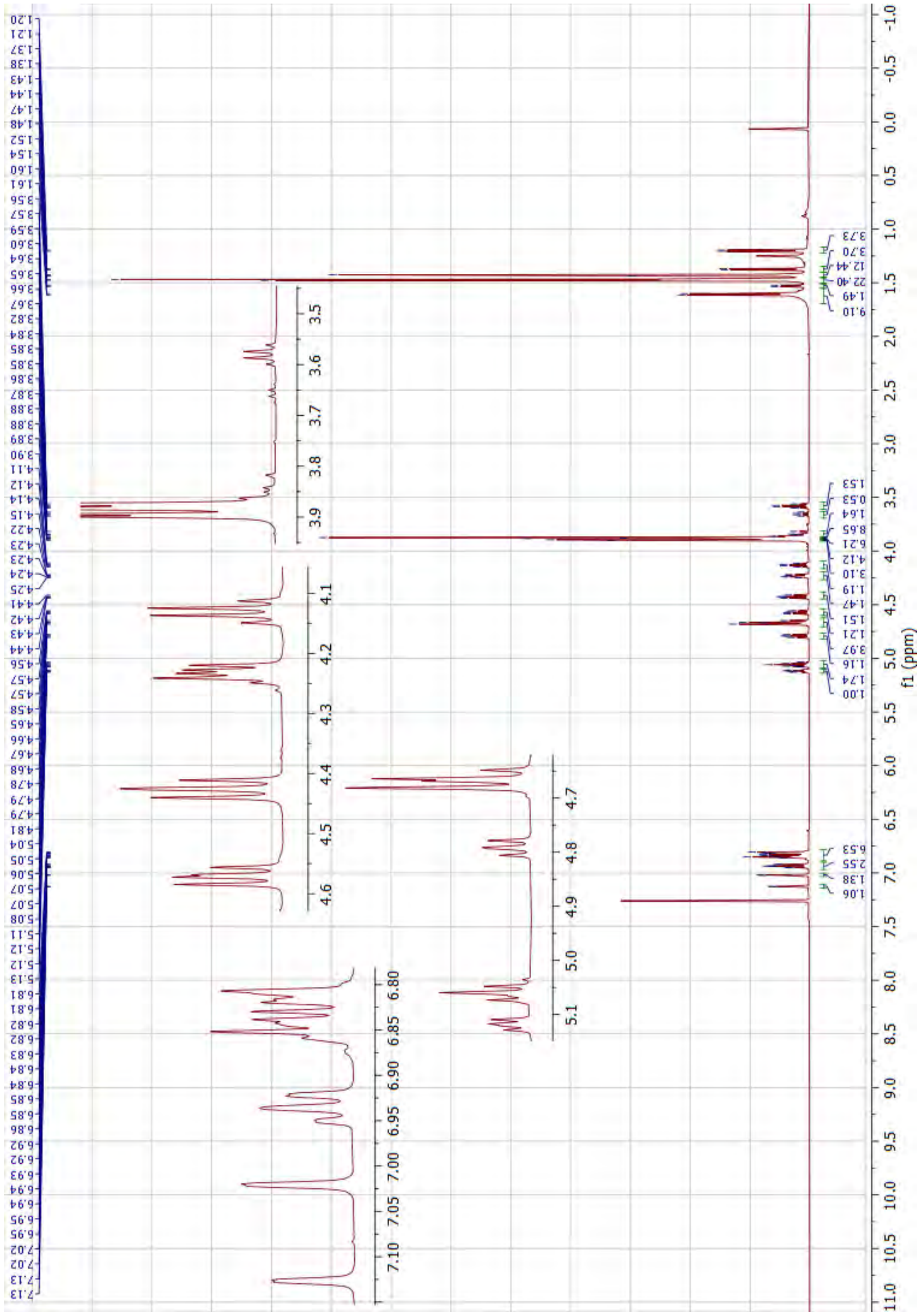

Figure S 14 ^{1}H NMR (600 MHz) of **11A** in $CDCl_3$

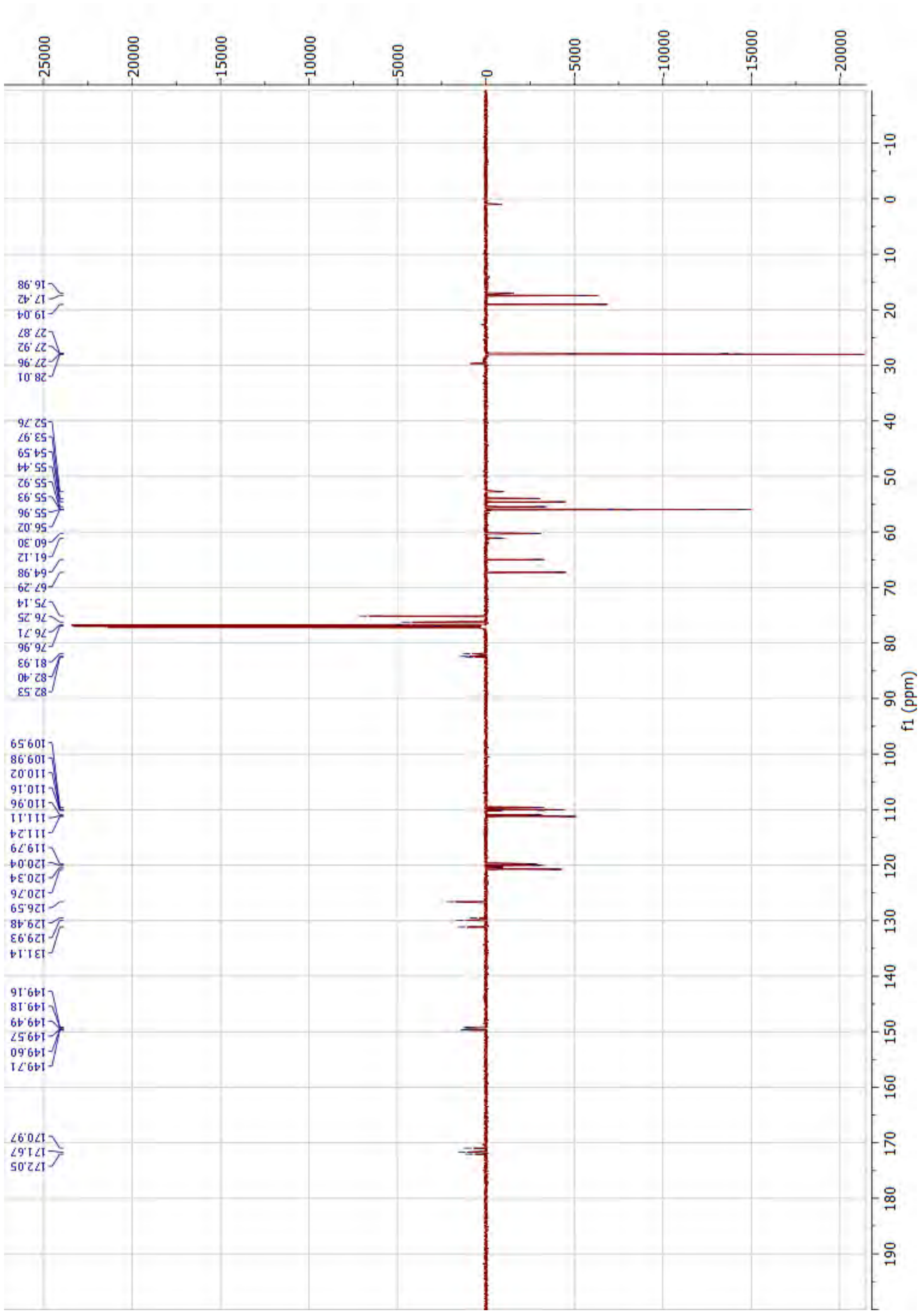

Figure S 15 ^{13}C NMR (151 MHz) of **11A** in $CDCl_3$

5.3. *tert*-butyl (2S)-2-(4-(3,4-dimethoxyphenyl)-2,2-dioxido-1,2,3-oxathiazolidin-3-yl)propanoate **12A**

11A (315 mg, 0.848 mmol, 1 equiv.) was dissolved in ACN (5 mL) and cooled to 0 °C. Then a catalytic amount of $RuCl_3xH_2O$ was added, followed by H_2O (5 mL) and sodium periodate (218 mg, 1.02 mmol, 1.2 equiv.). After 45 min the reaction was completed (monitored via TLC petroleum ether/ethyl acetate 1:1). The reaction mixture was extracted with DCM, the organic phase washed with brine and dried over $MgSO_4$. Purification via column chromatography (petroleum ether/ethyl acetate 1:1) gave the product in 86% yield (284 mg). Diastereomeric ratio 52:48, determined by integration of the α-proton of the amino acid (at 3.65 ppm and 4.27 ppm).

^{1}H NMR (600 MHz, $CDCl_3$, minor diastereomer) δ = 6.96 (d, *J*=2.1, 1H), 6.96 – 6.92 (m, 1H), 6.87 (d, *J*=8.1, 1H), 5.24 (dd, *J*=8.7, 6.9, 1H), 4.72 (dd, *J*=8.7, 6.9, 1H), 4.35 (t, *J*=8.7, 1H), 3.90 (s, 3H), 3.89 (s, 3H), 3.65 (q, *J*=7.4, 1H), 1.60 (d, *J*=7.4, 3H), 1.46 (s, 9H)

^{1}H NMR (600 MHz, $CDCl_3$, major diastereomer) δ = 7.04 (d, *J*=2.1, 1H), 6.96 – 6.92 (m, 1H), 6.84 (d, *J*=8.2, 1H), 5.40 (dd, *J*=7.3, 5.2, 1H), 4.91 (dd, *J*=8.7, 7.3, 1H), 4.27 (dd, *J*=8.7, 5.2, 1H), 4.21 (q, *J*=7.4, 1H), 3.90 (s, 3H), 3.88 (s, 3H), 1.49 (s, 9H), 1.24 (d, *J*=7.4, 3H).

^{13}C NMR (151 MHz, $CDCl_3$, both diastereomers) δ = 171.28 (major), 169.40 (minor), 150.03, 149.83, 149.66, 149.33, 131.78 (major), 127.47 (minor), 120.36 (minor), 118.92 (major), 111.34 (minor), 111.19 (major), 109.65 (minor), 109.16 (major), 82.54, 82.52, 73.35 (major), 72.47 (minor), 63.75 (minor), 60.06 (major), 56.02, 56.00, 55.96, 55.93, 54.69 (major), 53.73 (minor), 27.94, 27.91, 17.35 (major), 15.34 (minor).

HRMS (ESI): *m/z* calculated for $C_{17}H_{25}NO_7S+Na^+$: 410.1244 $[M+Na]^+$; found: 410.1243

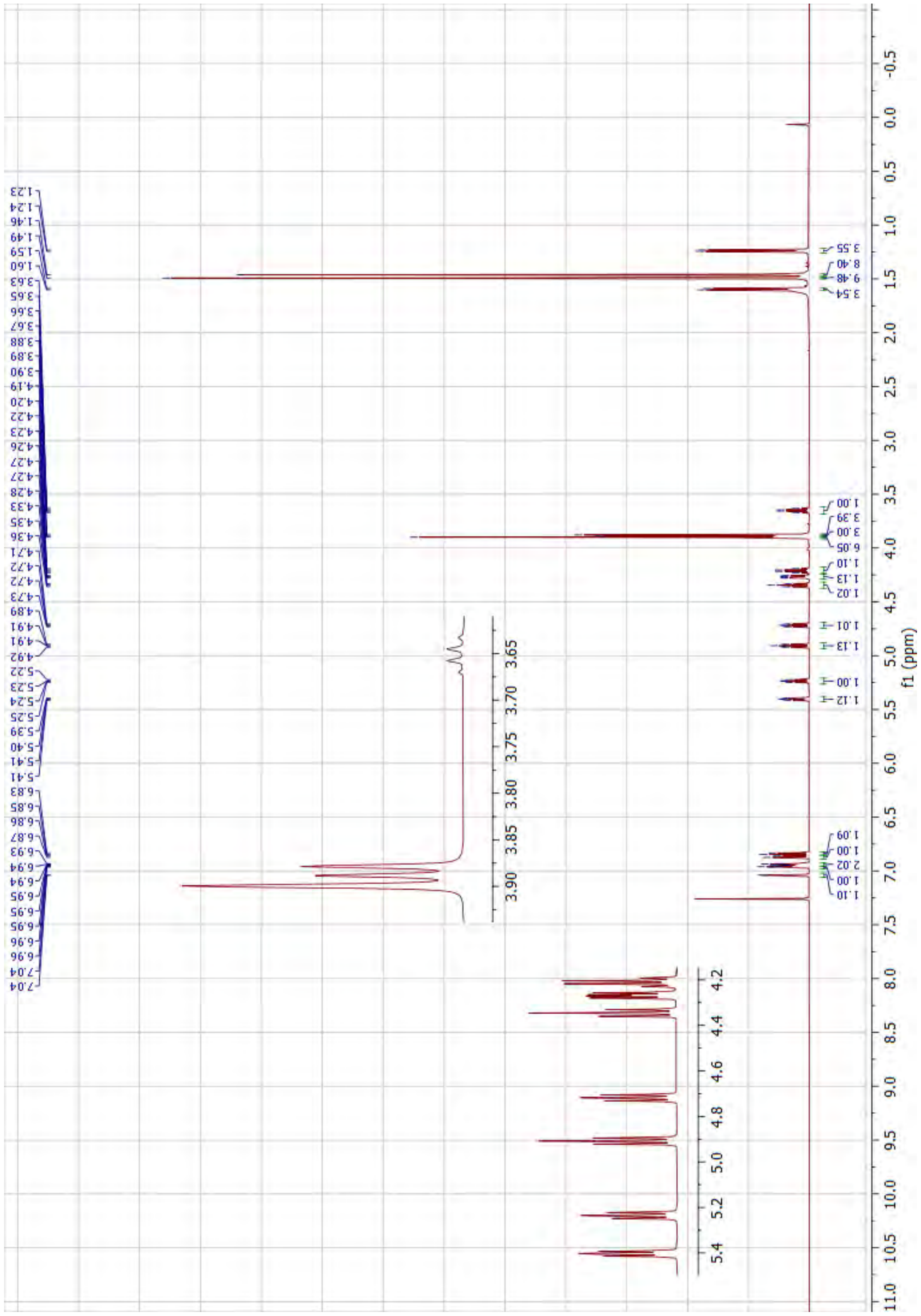

Figure S 16 ^{1}H NMR (600 MHz) of **12A** in $CDCl_3$

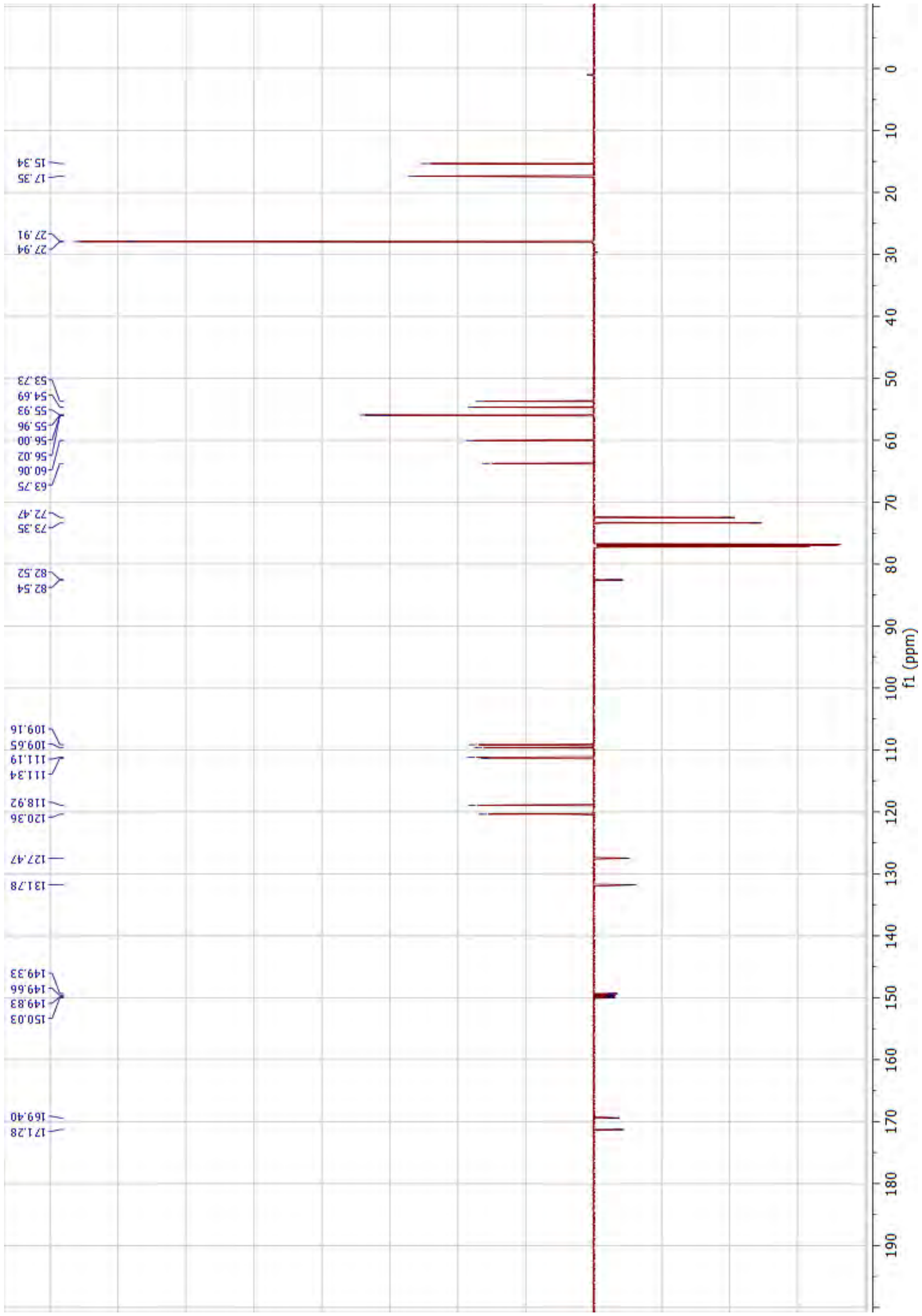

Figure S 17 ^{13}C NMR (151 MHz) of **12A** in $CDCl_3$

5.4. (2S)-2-(4-(4,5-dimethoxy-2-nitrophenyl)-2,2-dioxido-1,2,3-oxathiazolidin-3-yl)propanoic acid **13A**

Starting material **12A** (264 mg, 0.681 mmol, 1 equiv.) was dissolved in trifluoroacetic acid (TFA, 1 mL), the solution was cooled down to 0 °C and KNO_3 (75 mg, 0.75 mmol, 1.1 equiv.) was added. After 2 h the TFA was evaporated under a stream of nitrogen. The concentrate was taken up in DCM, washed with 1 M HCl followed by brine and the organic phase dried over $MgSO_4$. Evaporation of the solvent under reduced pressure gave the crude product (248 mg) that was used for the next step without further purification. Diastereomeric ratio 53:47, determined by integration of the benzylic protons (at 5.88 ppm and 6.06 ppm).

^{1}H NMR (600 MHz, CD_3OD, major diastereomer) δ = 7.80 (s, 1H), 7.63 (s, 1H), 6.06 (dd, *J*=7.3, 2.5, 1H), 5.26 (dd, *J*=8.8, 7.3, 1H), 4.49 – 4.41 (m, 2H), 3.97 (s, 3H), 3.93 (s, 3H), 1.34 (d, 3H).

^{1}H NMR (600 MHz, CD_3OD, minor diastereomer) δ = 7.78 (s, 1H), 7.61 (s, 1H), 5.88 (dd, *J*=7.2, 3.3, 1H), 5.20 (dd, *J*=9.0, 7.2, 1H), 4.49 – 4.41 (m, 1H), 4.22 (q, *J*=7.3, 1H), 3.98 (s, 3H), 3.93 (s, 3H), 1.64 (d, *J*=7.3, 3H).

^{13}C NMR (151 MHz, CD_3OD) δ = 174.57 (major), 173.61 (minor), 155.60, 155.58, 150.22, 150.20, 141.25 (minor), 140.61 (major), 132.17 (major), 130.85 (minor), 111.86 (major), 111.46 (minor), 109.79 (minor), 109.76 (major), 75.81 (major), 74.84 (minor), 62.19 (minor), 59.32 (major), 56.97, 56.92, 56.28 (major), 55.93 (minor), 17.61 (major), 15.52 (minor).

HRMS (ESI): *m/z* calculated for $C_{13}H_{16}N_2O_9S+Na^+$: 399.0469 $[M+Na]^+$; found: 399.0467

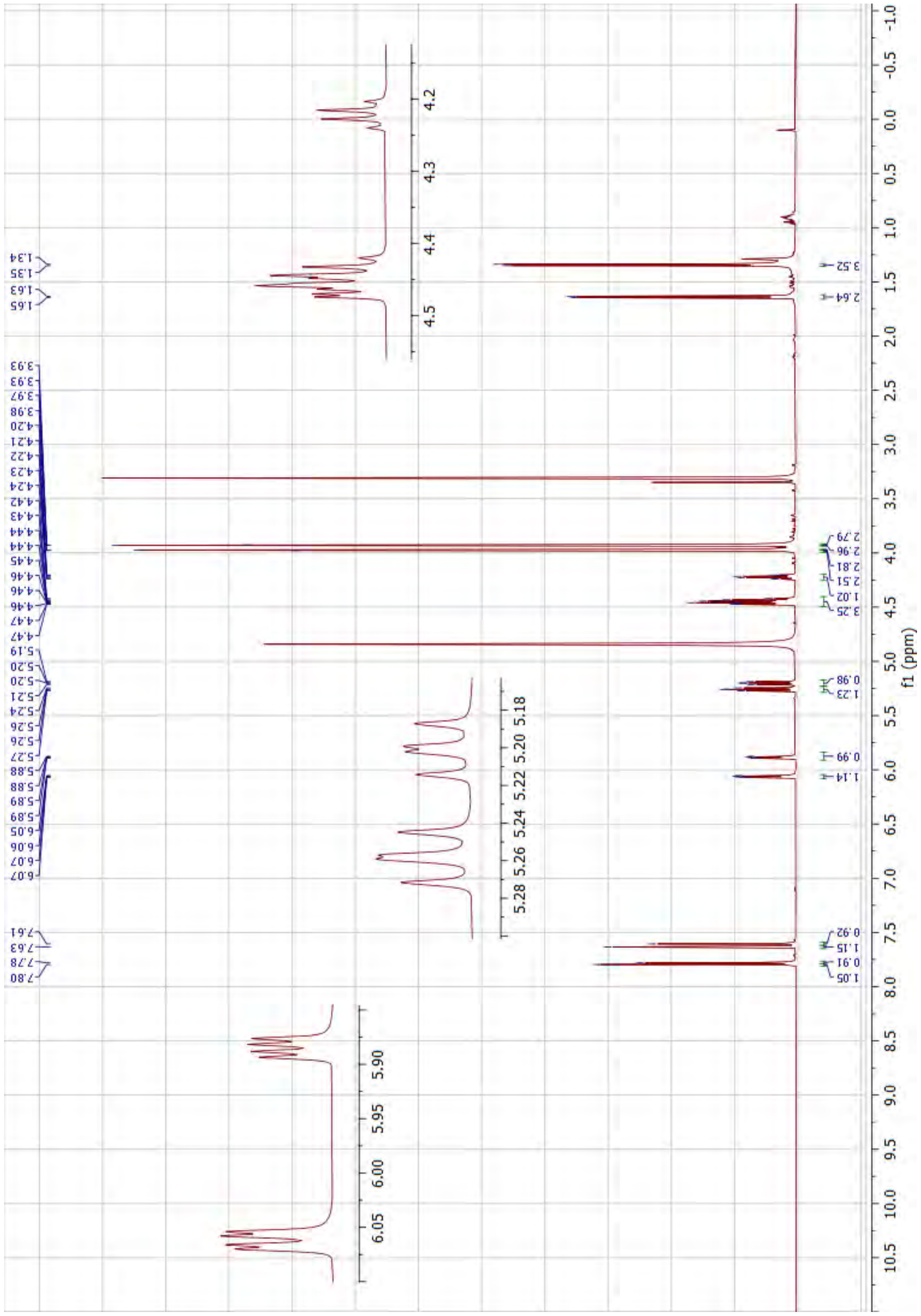

Figure S 18 ^{1}H NMR (600 MHz) of **13A** in CD_3OD

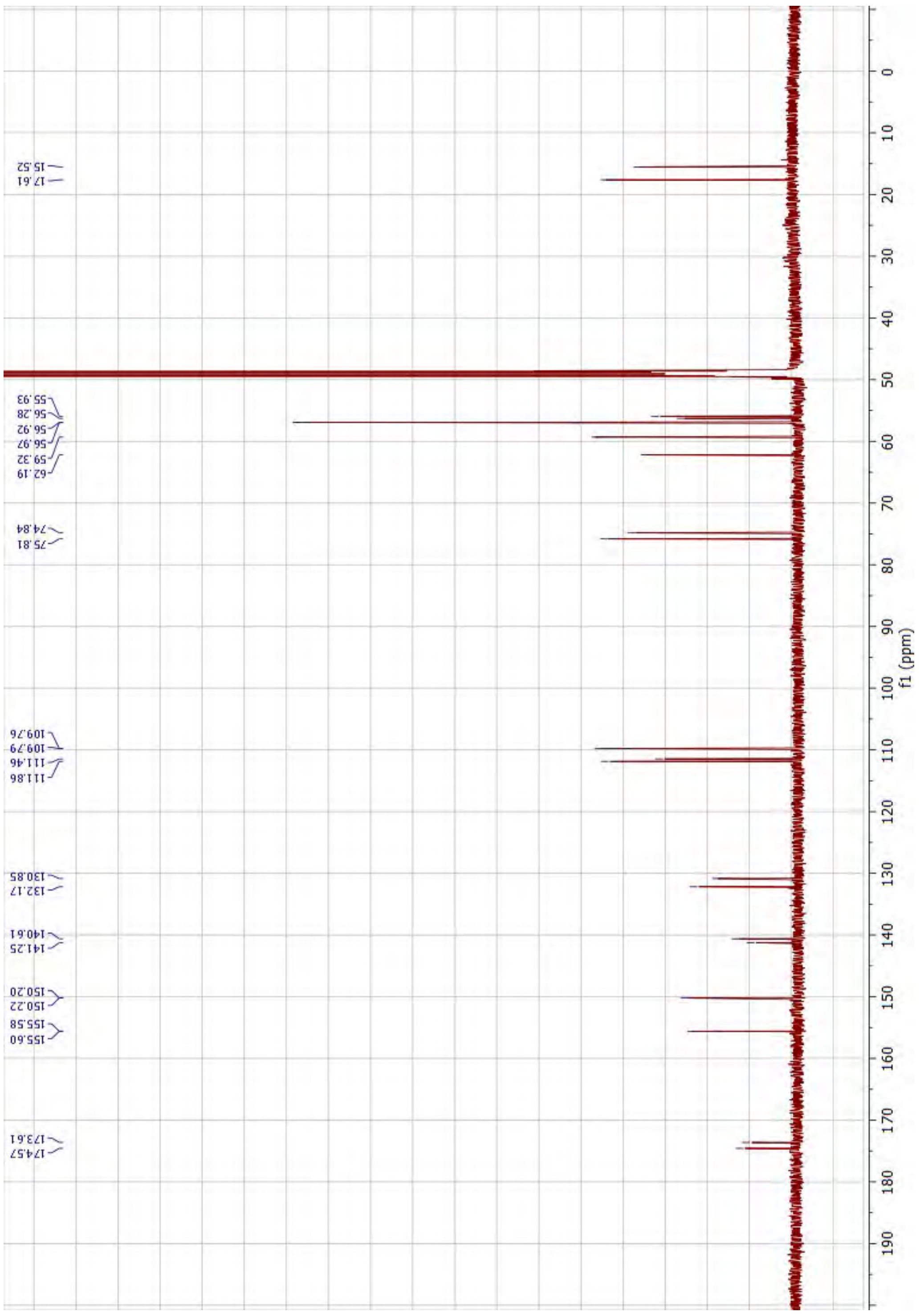

Figure S 19 ^{13}C NMR (151 MHz) of **13A** in CD_3OD

5.5. (1-(4,5-dimethoxy-2-nitrophenyl)-2-((4-methoxybenzyl)selanyl)ethyl)alanine **14A**

Dithiothreitol (21 mg, 0.14 mmol, 1 equiv.) and diselenide **S2** (41 mg, 0.1 mmol, 0.75 equiv.) were dissolved in anhydrous DMF (700 µL) under argon atmosphere and stirred for 15 min. Cs_2CO_3 (112 mg, 0.34 mmol, 2.5 equiv.) were added and stirred for an additional 15 min. Under a stream of argon, **13A** (50 mg, 0.14 mmol) was added. After 45 min an aqueous solution of HCl (2 M, 1 mL) was added, and stirred for 20 h. The solvent was evaporated under vacuum and gentle heating, followed by purification via column chromatography (DCM +10 % MeOH +AcOH). The solvent was evaporated under reduced pressure and the residue two times lyophilized (dissolved ACN/aqueous 1 M HCl 1:1, 2 mL) to give product **14A** in 14% yield over two steps as mixture of two diastereomers in a ratio of 57:43 (3.08-3.03 and 3.20 ppm).

^{1}H NMR (600 MHz, CD_3OD, major diastereomers) δ = 7.71 – 7.58 (m, 1H), 7.25 (br s, 1H), 7.15 (t, *J*=8.5, 2H), 6.82 (t, *J*=8.5, 2H), 5.41 (br s, 1H), 4.08 (br s, 1H), 3.96 (s, 3H), 3.95 (s, 3H), 3.78 (s, 3H), 3.73 – 3.68 (m, 2H), 3.30 – 3.28 (m, 1H)*, 3.08 – 3.03 (m, 1H), 1.59 (d, *J*=7.2, 3H).

* Overlap with methanol solvent peak.

^{1}H NMR (600 MHz, CD_3OD, minor diastereomers) δ = 7.71 – 7.58 (m, 1H), 7.38 (br s, 1H), 7.15 (t, *J*=8.5, 2H), 6.82 (t, *J*=8.5, 2H), 5.34 (s, 1H), 3.98 (s, 3H), 3.95 (s, 3H), 3.85 – 3.80 (m, 1H), 3.79 (s, 3H), 3.73 – 3.68 (m, 2H), 3.20 (dd, *J*=13.0, 5.2, 1H), 3.08 – 3.03 (m, 1H), 1.57 (d, *J*=7.2, 3H).

^{13}C NMR (151 MHz, CD_3OD) δ = 171.74 (minor), 171.56 (major), 160.27, 155.32, 155.11, 151.41, 151.20, 141.25**, 132.25, 131.43, 131.11, 131.09, 124.21 (major), 123.18 (minor), 115.13, 115.11, 111.15 (major)**, 111.05 (minor)**, 109.56, 108.94, 57.54, 57.36, 57.02 (major), 56.97, 56.96, 56.77 (minor)**, 56.61 (major)**, 56.04 (minor)**, 55.69, 55.68, 28.50 (major), 28.19 (minor), 25.86 (major)**, 25.51, 16.33 (minor), 15.68 (major).
**Due to peak broadening in 1D ^{13}C NMR spectrum, chemical shifts were taken from ^{1}H-^{13}C HSQC cross peaks.

HRMS (ESI): *m/z* calculated for $C_{21}H_{26}N_2O_7$+H^+: 499.0978 [*M*+H]$^+$; found: 499.0981

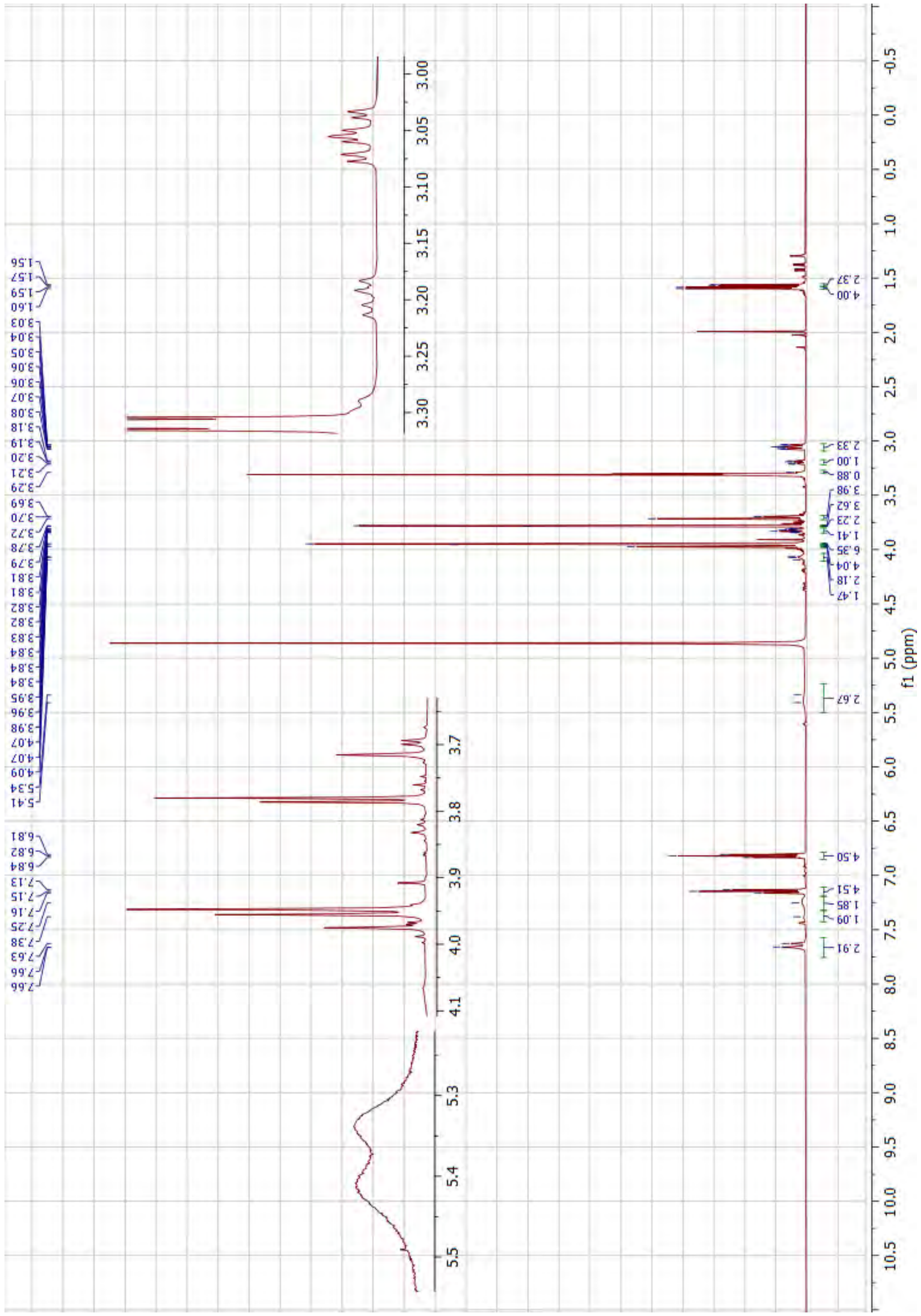

Figure S 20 ^{1}H NMR (600 MHz) of **14A** in CD_3OD

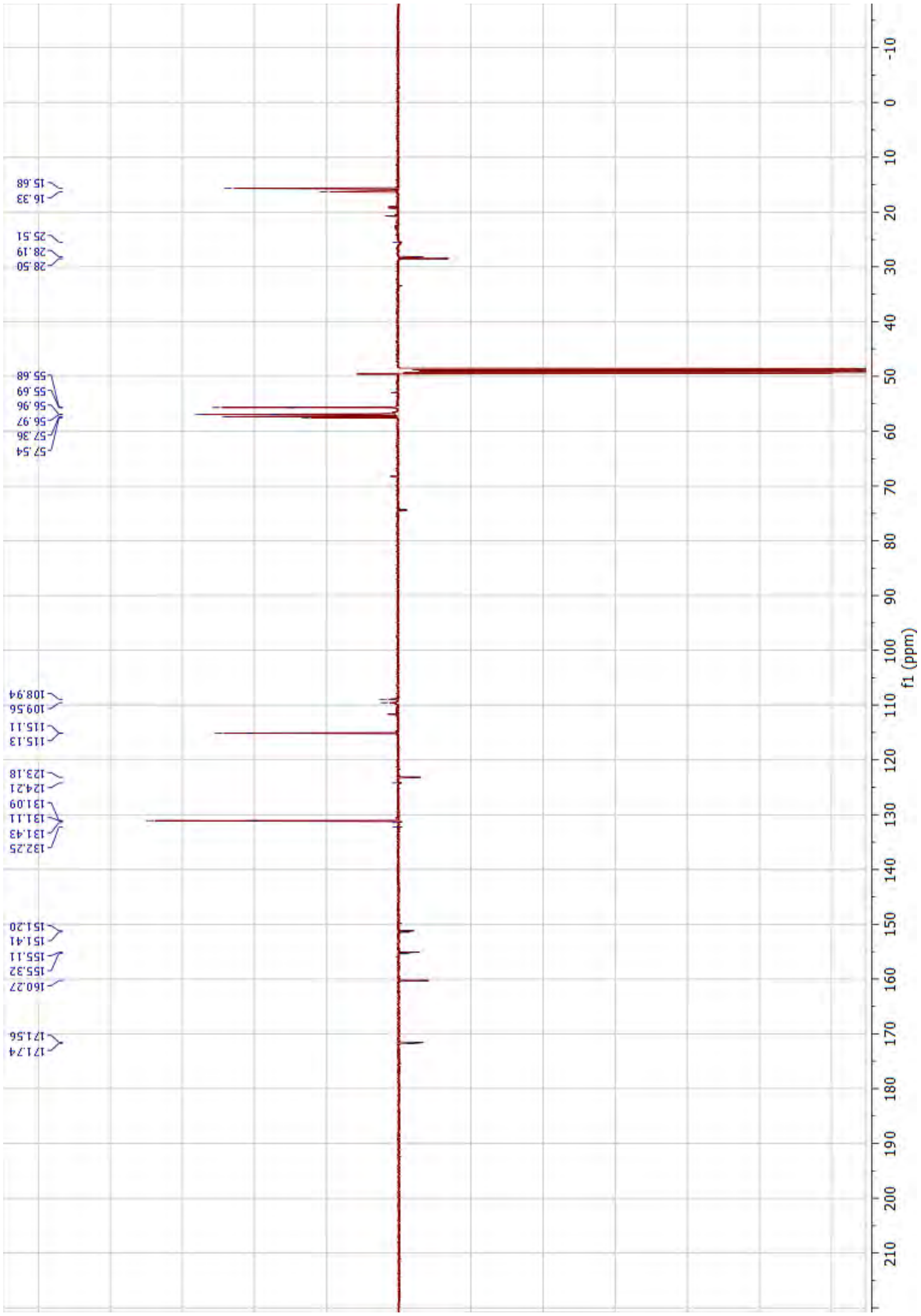

Figure S 21 ^{13}C NMR (151 MHz) of **14A** in CD_3OD

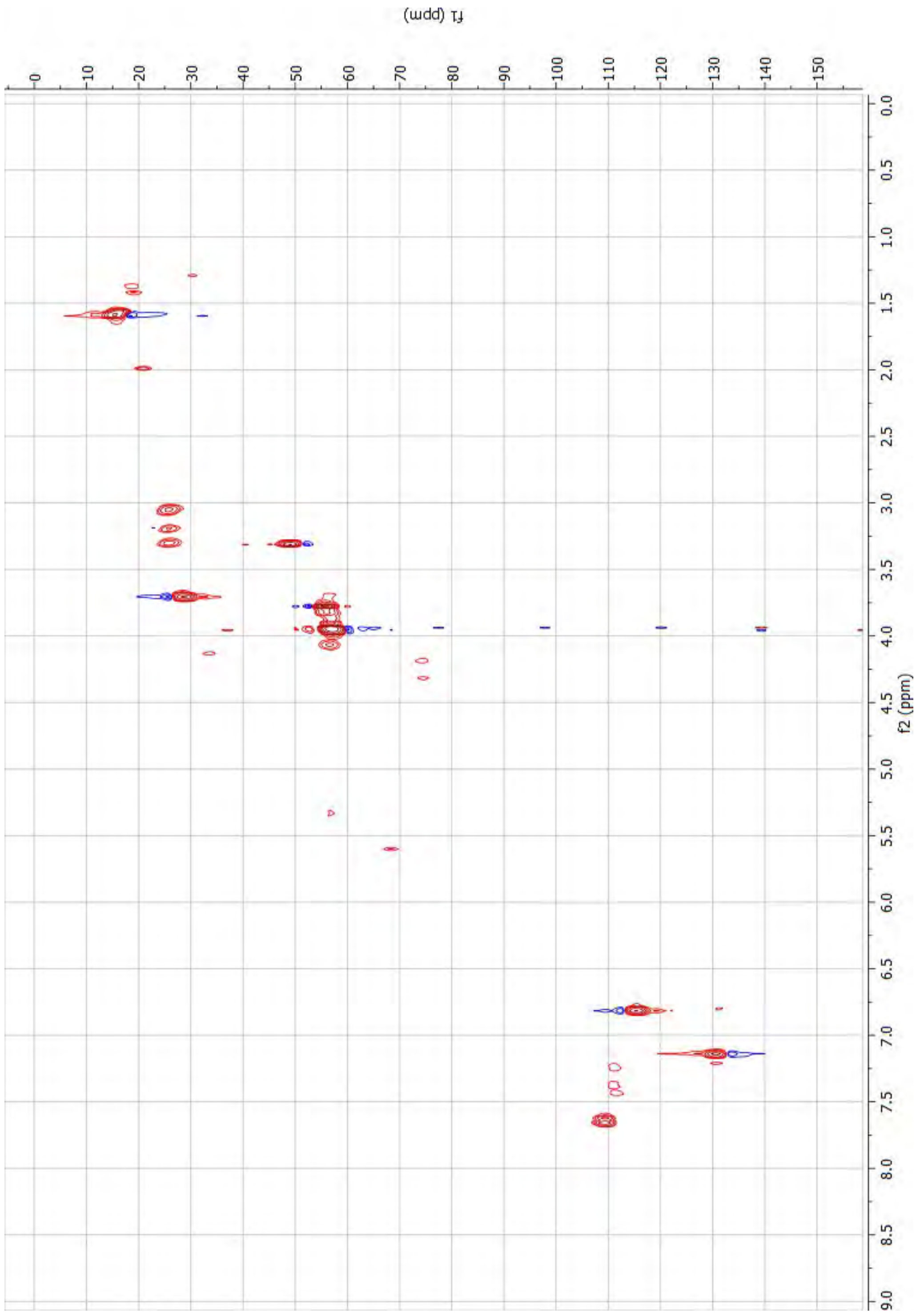

Figure S 22 ^{1}H-^{13}C HSQC spectrum of **14A** in CD_3OD

6. Peptide synthesis

6.1. General procedure solid phase peptide synthesis

Manual peptide synthesis was performed, unless otherwise stated, using the following protocol. Preloaded TentaGel® R PHB resin was swollen in DMF for 2 h prior to synthesis. After swelling, the peptide was elongated via repeated cycles of deprotection and coupling with the required amino acids orthogonally protected. Briefly: (1) deprotection of the N-terminal Fmoc protecting group by treating the peptidyl resin twice with a solution of piperidine in DMF (20% v/v, 3 + 7 min); (2) flow wash for 1 min; (3) coupling of the amino acid by addition of a solution of the protected amino acid, HATU and DIEA in DMF (2.5 equiv., 2.4 equiv. and 5 equiv., respectively); (4) shaking of the suspension for 20 min at room temperature; (5) solvent removal and washing of the peptidyl resin with DMF. After final Fmoc-deprotection, the resin was washed with DMF and DCM and dried in a desiccator overnight. The standard cleavage was performed using a cocktail of 92% TFA, 5% TIPS, 2.5% MQ-H_2O for 2 h. The solution was concentrated under argon flow or the crude was directly precipitated using ice-cold diethyl ether (ca. 5-8 fold volume), centrifuged (4000 rpm, 5 min), dried under argon flow, dissolved in MQ-H_2O /acetonitrile 1:1 (+ 0.1% TFA) and purified via HPLC after lyophilization.

Automated peptide synthesis was performed on a PTI Tribute synthesizer (Protein Technologies, Inc., USA). Fmoc-deprotection: 20% Piperidine in DMF, 2x5 min; Coupling: 2.5 equiv. Fmoc-protected amino acid, 2.25 equiv. HBTU (0.45 M in DMF), 5 equiv. DIPEA (1 M in DMF), 30 min

Microwave assisted peptide synthesis was performed on a CEM Liberty Blue synthesizer. Fmoc deprotection: 20% Piperidine in DMF (+ 0.1 M Oxyma), 90 °C for 75 sec; coupling: 5 equiv. Fmoc protected amino acid, 10 equiv. DIC (1 M in DMF), 5 equiv. Oxyma (1 M in DMF), 90 °C 265 sec

6.2. General procedure resin hydrazine loading

Hydrazination of the 2-chlorotrityl resin was performed following a modified protocol by Li *et. al.*[21] 2-CTC resin (1.45 mmol/g) was swollen in DMF/DCM 1:1 for 30 min and washed 3x with DMF. Hydrazine monohydrate (10% in DMF, 15 mL/g resin) was added to the resin, agitated for 30 min and washed with DMF. This step was performed in total two times. To cap unreacted sites, the resin was then incubated in 5% MeOH in DMF (15 mL/g resin) for 20 min. After final washing with DMF and DCM, the resin was dried in a desiccator and stored at -18 °C.

To load the C-terminal amino acid of the sequence, the hydrazine resin was first swollen in DMF for 30 min, then 2.5 equiv. of the Fmoc-amino acid, 2.4 equiv. of 0.5 M HATU and 5 equiv. of DIEA were added to the resin and the suspension was incubated at r.t. for 30 min. After washing with DMF, this step was repeated. After final washing with DMF and DCM, the resin was thoroughly dried in a desiccator overnight.

A 10 mL measuring flask with dry resin (ca. 10 mg exact) was filled with 20% piperidine in DMF and incubated for 30 min. Then, 500 µL were taken out and diluted with 20% piperidine in DMF to 5 mL, the absorbance measured at a wavelength of 301 nm and the loading calculated according to Eq. (1).

$$Loading\ [mmol\ g^{-1}] = \frac{A \times d}{\varepsilon \times l \times m} \times V \times 10^{3} \quad \textbf{Eq. (1)}$$

A – UV absorbance at 301 nm
ε – extinction coefficient for dibenzofulvene adduct (7731.9 mol^{-1} L cm^{-1})
m – mass of resin [mg]
d – dilution faction (10)
l – length of cuvette (1 cm)
V – volume (10 mL)

Peptides were synthesized on this preloaded hydrazide resin according to the general procedure above and cleaved (section 6.1).

6.3. LYRAX-SePh **17X** synthesis and analysis

LYRAG-SePh **17G**

The selenoester was prepared according to the general procedure described in the experimental section of the manuscript and Section 6.2 (0.014 mmol scale) and purified via preparative HPLC (Kromasil 300-10-C4, 21.2x250 mm prep, 5-65% buffer B (ACN +0.08% TFA) in buffer A (MQ-H_2O +0.1% TFA) in 50 min), giving the product in 54% yield (based on crude, 5.4 mg, see Figure S23).

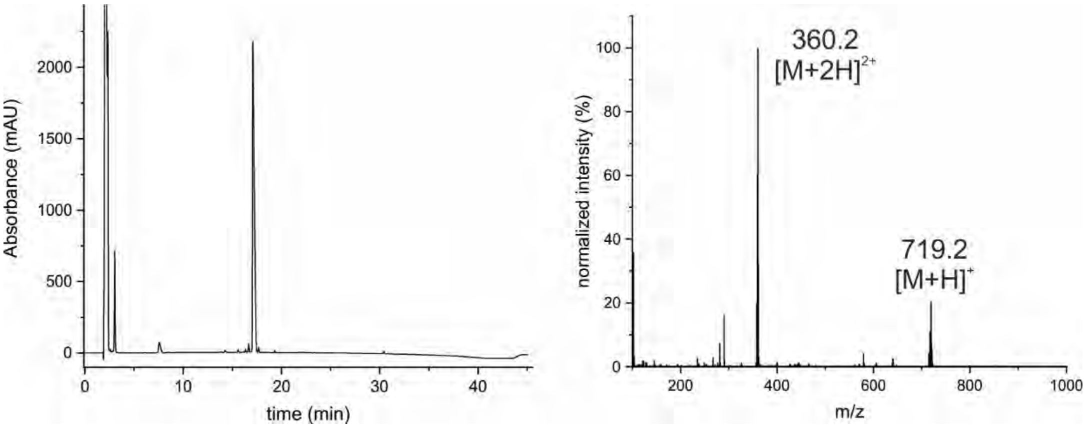

Figure S23 HPLC chromatogram (5-65% B in 30 min) and ESI-MS direct injection of peptide **17G**

LYRAL-SePh **17L**

The selenoester was prepared according to the general procedure described in Section 6.2 (0.046 mmol scale) and purified via preparative HPLC (Kromasil 300-10-C4, 21.2x250 mm prep, 5-65% buffer B (ACN +0.08% TFA) in buffer A (MQ-H_2O +0.1% TFA) in 50 min), giving the product in 50% yield (based on crude, 19.7 mg, see Figure S 24).

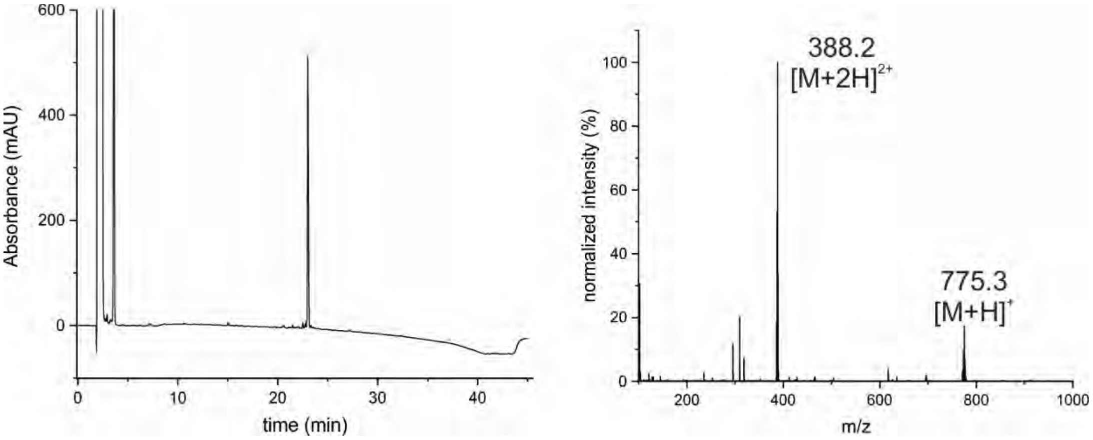

Figure S 24 HPLC chromatogram (5-65% B in 30 min) and ESI-MS direct injection of peptide **17L**

LYRAI-SePh **17I**

The selenoester was prepared according to the general procedure described in the experimental section of the manuscript and Section 6.2 (0.014 mmol scale) and purified via preparative HPLC (Kromasil 300-10-C4, 21.2x250 mm prep, 5-65% buffer B (ACN +0.08% TFA) in buffer A (MQ-H_2O +0.1% TFA) in 50 min), giving the product in 33% yield (based on crude, 8.2 mg, see Figure S 25).

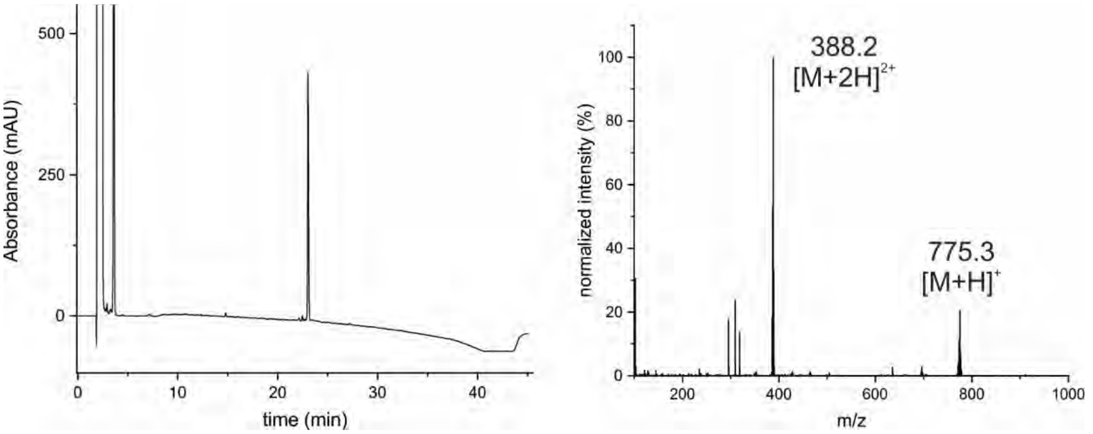

Figure S 25 HPLC chromatogram (5-65% B in 30 min) and ESI-MS direct injection of peptide **17I**

LYRAA-SePh **17A**

The selenoester was prepared according to the general procedure described in the experimental section of the manuscript and Section 6.2 (0.014 mmol scale) and purified via preparative HPLC (Kromasil 300-10-C4, 21.2x250 mm prep, 5-65% buffer B (ACN +0.08% TFA) in buffer A (MQ-H_2O +0.1% TFA) in 50 min), giving the product in 37% yield (based on crude, 9 mg, see Figure S 26).

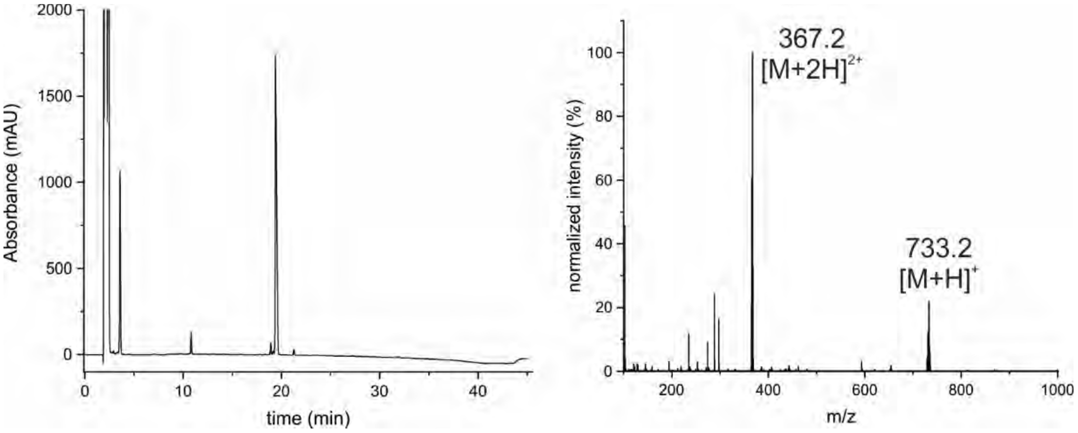

Figure S 26 HPLC chromatogram (5-65% B in 30 min) and ESI-MS direct injection of peptide **17A**

LYRAT-SePh **17T**

The selenoester was prepared according to the general procedure described in the experimental section of the manuscript and Section 6.2 (0.014 mmol scale) and purified via preparative HPLC (Kromasil 300-10-C4, 21.2x250 mm prep, 5-65% buffer B (ACN +0.08% TFA) in buffer A (MQ-H_2O +0.1% TFA) in 50 min), giving the product in 33% yield (based on crude, 10.3 mg, see Figure S 27).

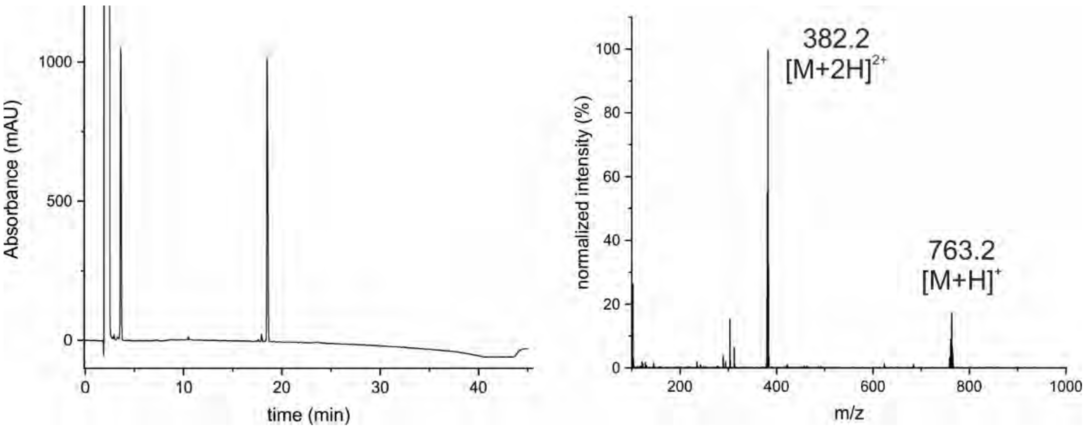

Figure S 27 HPLC chromatogram (5-65% B in 30 min) and ESI-MS direct injection of peptide **17T**

LYRAV-SePh **17V**

The selenoester was prepared according to the general procedure described in the experimental section of the manuscript and Section 6.2 (0.014 mmol scale) and purified via preparative HPLC (Kromasil 300-10-C4, 21.2x250 mm prep, 5-65% buffer B (ACN +0.08% TFA) in buffer A (MQ-H_2O +0.1% TFA) in 50 min), giving the product in 42% yield (based on crude, 13.8 mg, see Figure S 28).

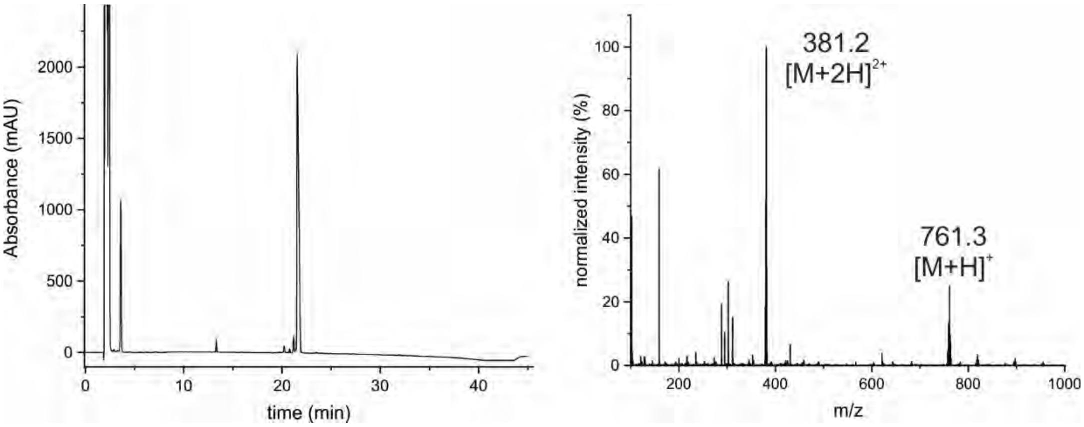

Figure S 28 HPLC chromatogram (5-65% B in 30 min) and ESI-MS direct injection of peptide **17V**

LYRAa-SePh **17a**

Fmoc-hydrazine resin was synthesized according to a modified procedure by Bird et. al.[26d] 200 mg 2-CTC resin (1.6 mmol/g loading, 0.32 mmol) were swollen in DMF/DCM 1:1 for 30 min. To load the resin, 2.5 equiv. (0.8 mmol, 203 mg) of Fmoc-hydrazine were dissolved in DMF/DCM (5:3) and added to the drained resin for 45 min, two times. Afterwards, the resin was washed with DMF and capped with a solution of 10 equiv. DIPEA in MeOH for 30 min.

The loading was determined according to Section 6.2. After manual peptide synthesis (section 6.1) and cleavage from the resin, the crude peptide hydrazide was converted into the selenoester described in the experimental section of the manuscript at a 0.013 mmol scale. After purification via semipreparative HPLC (Kromasil 300-10-C4 10x250 mm semiprep, 5-65% buffer B (ACN +0.08% TFA) in buffer A (MQ-H_2O +0.1% TFA) in 50 min), the product could be obtained in 51% yield (based on crude, 5 mg, see Figure S 29).

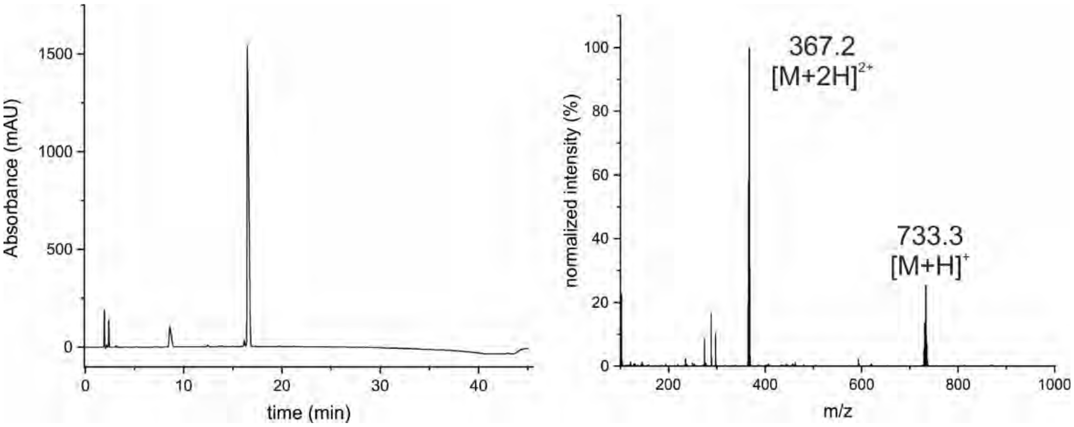

Figure S 29 HPLC chromatogram (5-65% B in 30 min) and ESI-MS direct injection of peptide **17a**

6.4. Gly(SeAUX)-peptide **16G** synthesis

Peptide **16G** was synthesized via microwave-assisted solid phase peptide synthesis as described above (Section 6.1). The auxiliary was manually coupled to the resin (73 mg, ca. 14 µmol,) using 1.25 equiv. of Gly(SeAUX) **14G**, 1.25 equiv. of Oxyma (0.5 M in DMF) and 1.25 equiv. DIC for 2 h. Global deprotection was done with a cleavage cocktail consisting of 87% TFA, 5% TIPS, 5% DMDS, 2.5% MQ-H_2O for 2 h. After precipitation, the residue was dissolved in 500 µL of ACN/MQ-H_2O +0.1% TFA and lyophilized. The crude peptide was dissolved in a 6 M aqueous solution of Gdn-HCl (pH 4.7), 2 equiv. DTT (100 mM in MQ-H_2O) were added and the solution shaken at room temperature for 10 min. Direct purification via semipreparative HPLC (Kromasil 300-10-C4 10x250 mm semiprep, 5-65% buffer B (ACN +0.08% TFA) in buffer A (MQ-H_2O +0.1% TFA) in 50 min) gave the product in 24% yield (3.3 mg, 3.4 µmol, based on synthesis scale, calculated for the reduced monomer, see Figure S 30).

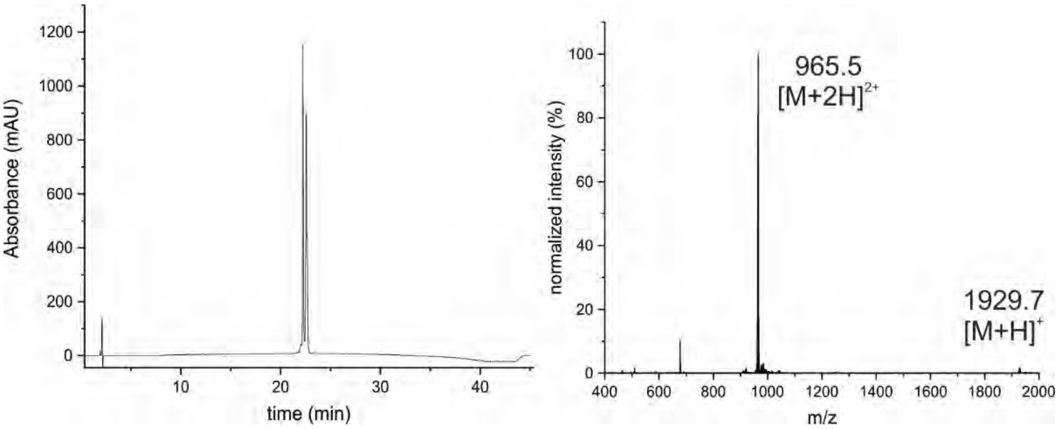

Figure S 30 HPLC chromatogram (5-65% B in 30 min) and ESI-MS direct injection of peptide **16G**

6.5. Ala(SeAUX)-peptide **16A** synthesis

Peptide **16A** was synthesized via microwave-assisted automated solid phase peptide synthesis as described above (see section 6.1). The auxiliary was manually coupled to the resin (42 mg, ca. 12.5 µmol) using 1.9 equiv. of Ala(SeAUX) **14A**, 1.9 equiv. of Oxyma (0.5 M in DMF) and 1.9 equiv. DIC for 2 h. Global deprotection was done with a cleavage cocktail consisting of 87% TFA, 5% TIPS, 5% DMDS, 2.5% MQ-H_2O for 2 h. After precipitation the residue was dissolved in ca. 500 µL ACN/MQ-H_2O +0.1% TFA and lyophilized. The crude peptide was dissolved in a 6 M aqueous solution of Gdn-HCl (pH 4.7), 2 equiv. DTT (100 mM in MQ-H_2O) were added and the solution shaken at room temperature for 10 min. Direct purification via semipreparative HPLC (Kromasil 300-10-C4 10x250 mm semiprep, 5-65% buffer B (ACN +0.08% TFA) in buffer A (MQ-H_2O +0.1% TFA) in 50 min) gave the product in 23% yield (2.9 mg, 3 µmol, based on synthesis scale, calculated for the reduced monomer, see Figure S 31).

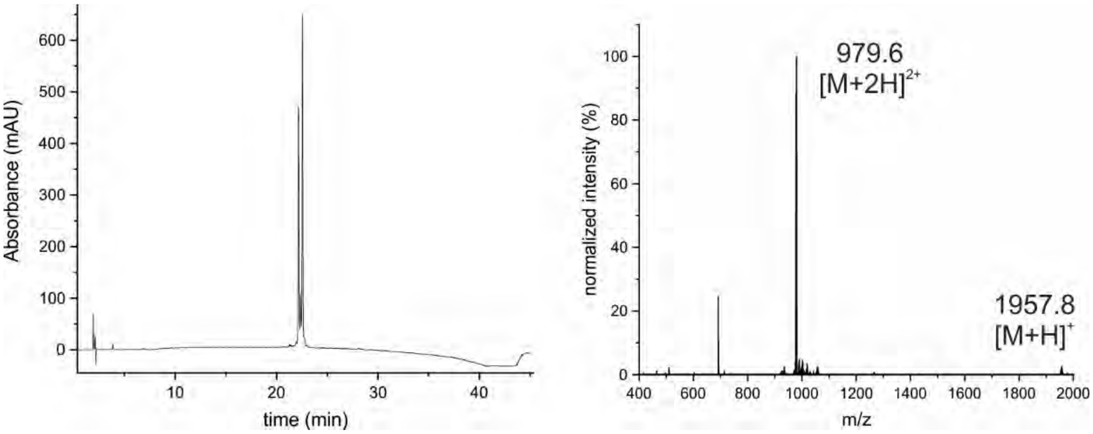

Figure S 31 HPLC chromatogram (5-65% B in 30 min) and ESI-MS direct injection of peptide **16A**

6.6. Gly(SeAUX)-G-CSF 126-174 peptide **29** synthesis

Gly(SeAUX)-G-CSF 126-174 **29** was synthesized using the automated peptide synthesis procedure (Section 6.1). The auxiliary **14A** was coupled to the resin (34 mg, ca. 5.8 µmol) using 2.5 equiv. of HATU (0.5 M in DMF), 2.4 equiv. DIEA for 3.5 h. Global deprotection was done with a cleavage cocktail consisting of 87% TFA, 5% TIPS, 5% DMDS, 2.5% MQ-H_2O for 2 h. After precipitation the residue was dissolved in 500 µL ACN/MQ-H_2O +0.1% TFA and lyophilized. The crude peptide was dissolved in a 6 M aqueous solution of Gdn-HCl (pH 4.7), 2 equiv. DTT (100 mM in MQ-H_2O) were added and the solution shaken at room temperature for 10 min. Direct purification via semipreparative HPLC Kromasil 300-10-C4 10x250 mm semiprep, 5-95% buffer B (ACN +0.08% TFA) in buffer A (MQ-H_2O +0.1% TFA) in 40 min) at 60 °C gave the product in 6% yield (1.9 mg, 0.3 µmol, based on synthesis scale, calculated for the reduced monomer, see Figure S 32).

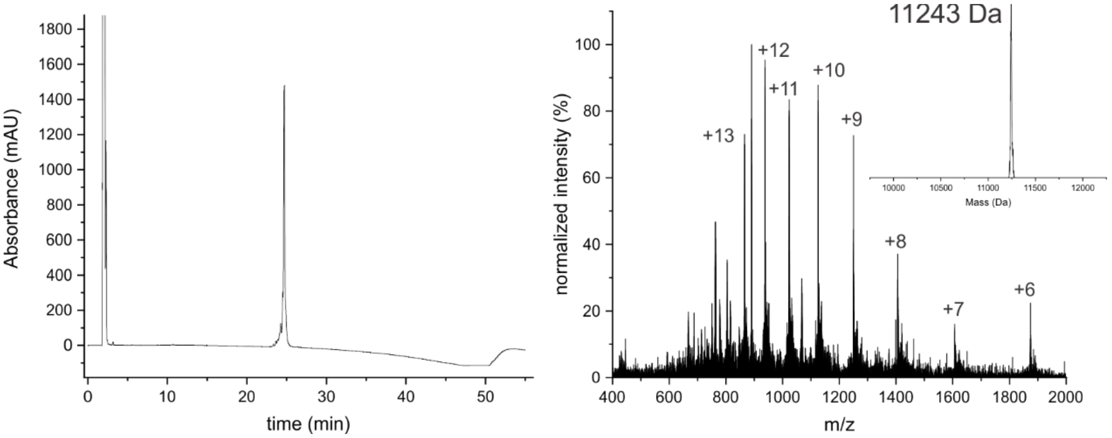

Figure S 32 HPLC chromatogram (5-95% B in 40 min at 60 °C) and ESI-MS direct injection of peptide Gly(SeAUX)-G-CSF 126-174 **29**; calculated mass: 11244 Da

6.7. Synthesis of peptides aGVTSWA **Da-25** and AGVTSWA **25**

Peptides **Da-25** and **25** were manually synthesized according to the general method outlined above (section 6.1), using preloaded Tentagel R resin (0.21 mmol/g), at a 0.025 mmol scale. After semipreparative HPLC (Kromasil 300-10-C4 10x250 mm semiprep, 5-65% buffer B (ACN +0.08% TFA) in buffer A (MQ-H_2O +0.1% TFA) in 50 min) peptide **Da-25** (see Figure S 33) could be isolated with a yield of 45% (7.8 mg) and **25** (see Figure S 34) with a yield of 22% (3.8 mg).

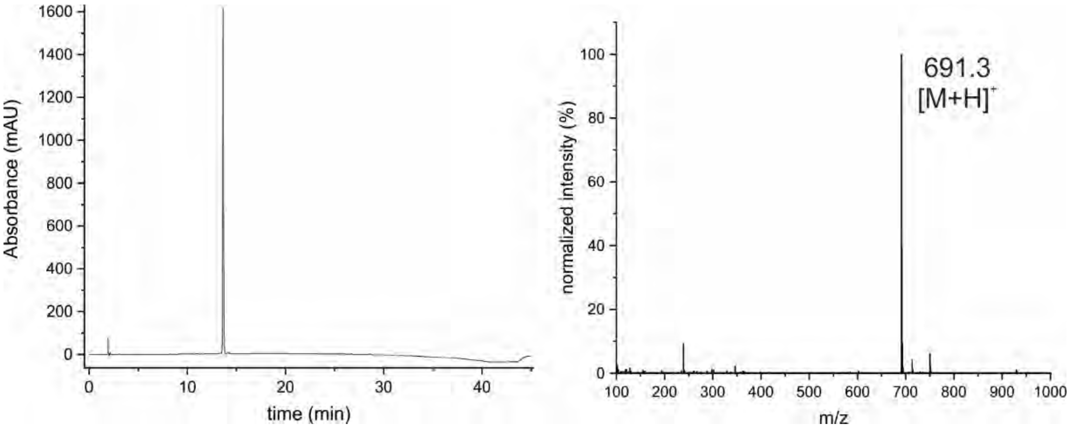

Figure S 33 HPLC chromatogram (5-65% B in 30 min) and ESI-MS direct injection of peptide **25**

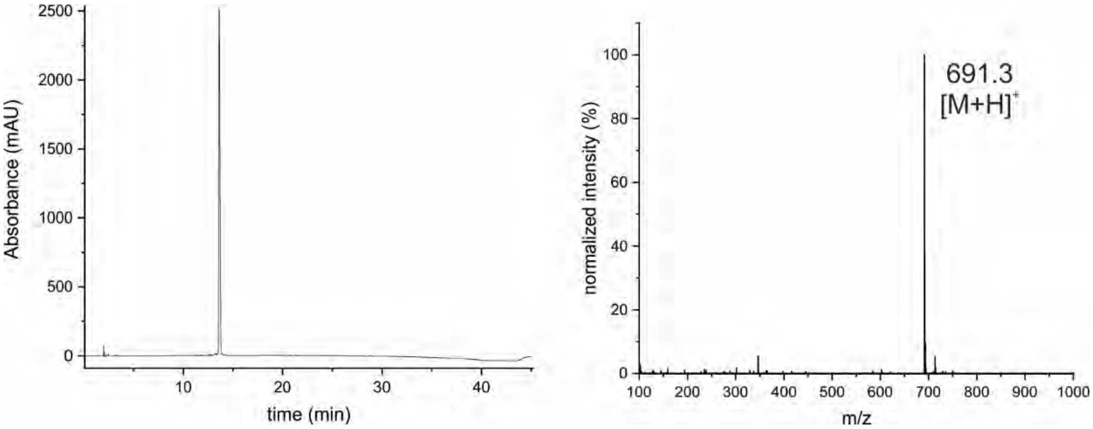

Figure S 34 HPLC chromatogram (5-65% B in 30 min) and ESI-MS direct injection of peptide **Da-25**

7. G-CSF selenoester synthesis

7.1. Expression and purification of G-CSF 1-124-$NHNH_2$ **27**

The expression plasmid containing G-CSF 1-124 C-terminally fused to Mxe-GyrA inteine, a HIS7 tag and a chitin binding domain (CBD) was already available in our laboratory.[11] Expression was carried out in *E. coli* Rosetta2(DE3) strain (Novagen) using 2YT medium (16 g/l tryptone, 10 g/l yeast extract, 5 g/l NaCl) containing 100 µg/mL ampicillin and 30 µg/mL chloramphenicol. Overnight cultures were diluted to OD600= 0.2, grown at 37 °C until OD600= 0.7 and overexpression was induced with 1 mM isopropyl thiogalactopyranoside (IPTG). After 4 h, cells were harvested by centrifugation (10.000 g), cell pellets were resuspended in TBS (50 mM Tris, 150 mM NaCl, pH 7.5) buffer and lysed by passing twice through a high-pressure cell disrupter (Constant Systems). The lysate was centrifuged (60.000 g) and the insoluble fraction, containing the expressed G-CSF-Mxe-HIS7-CBD **26** in inclusion bodies, was washed 1x with TBS +0.1% Tween and 2x with TBS followed by solubilization in a 6 M aqueous solution of Gdn-HCl (pH 4.7, 10 mL/L *E. coli* culture) overnight. The resulting protein solution was centrifuged (60.000 g) and the clear supernatant was loaded on a NiNTA column (GE Healthcare His-trap HP 5 mL) equilibrated with 6 M Gdn-HCl in TBS pH 8. After washing with 5 column volumes of 6 M Gdn-HCl in TBS pH 8 the protein was eluted from the column with a gradient of 0–300 mM imidazole in 6 M Gdn-HCl in TBS pH 8 over 60 min. The fractions containing clean G-CSF-Mxe-HIS7-CBD **26** were identified by SDS-PAGE, pooled and concentrated to a volume of 10-15 mL using centrifugal filters with 10 kDa MWCO (Amicon, Millipore). The buffer of the concentrated protein solution was exchanged to an 8 M solution of urea in TBS at pH 8 using PD10 columns (Cytiva). Hydrazine cleavage of the Mxe-GyrA intein was induced by dropwise addition of a cleavage solution containing hydrazine and DTT in TBS in order to reach the final cleavage conditions of 2 % hydrazine, 100 mM DTT and 2 M urea in TBS. After overnight cleavage at room temperature, around 1/5 volume of a 6 M aqueous solution of Gdn-HCl (pH 4.7) was added to dissolve the precipitated protein and the pH was lowered to 8.5 by addition of a 6 M aqueous solution of HCl. The resulting solution was filtered and purified by RP-HPLC on a preparative C4 column with a gradient of 5–30% buffer B (ACN +0.08% TFA) over 5 min and 30-80% buffer B over 30 min in buffer A (MQ-H_2O +0.1% TFA) at a flow rate of 10 mL/min. Fractions containing the G-CSF 1-124-$NHNH_2$ **27** were identified by mass spectroscopy, pooled, lyophilized and analyzed (8 mg, 2.7 mg/L culture, see Figure S 35).

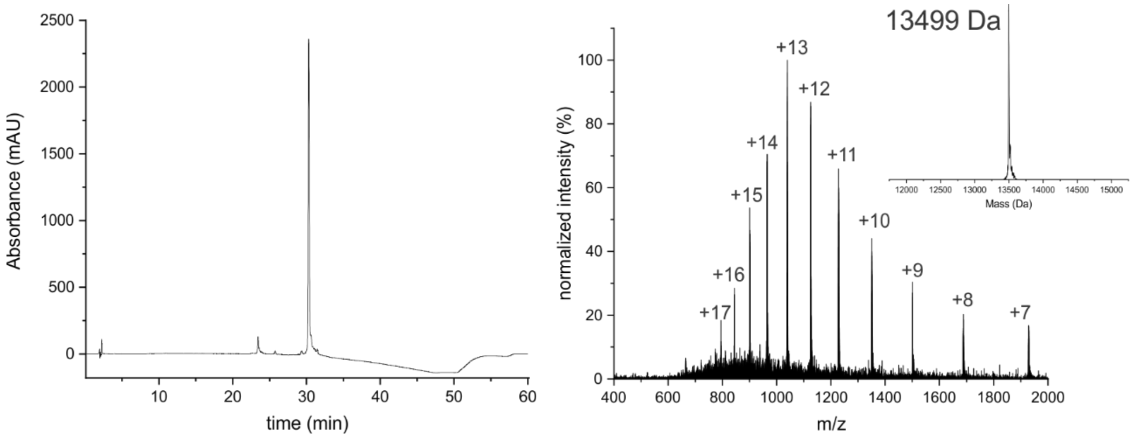

Figure S 35 HPLC chromatogram (5-95% B in 40 min, 60 °C) and ESI-MS direct injection of peptide G-CSF 1-124-$NHNH_2$ **27**

7.2. Conversion of G-CSF 1-124 hydrazide **27** to selenoester **28**

For the conversion of G-CSF 1-124 hydrazide **27** into G-CSF 1-124 selenoester **28,** 3 mg (0.22 µmol) of hydrazide were dissolved in conversion buffer (1.5 mL of 6 M Gdn-HCl, 200 mM HEPES, 200 mM TCEP, 50 mM DPDS, pH 1.5, 15 min sonication) at a concentration of 0.15 mM. 7 µL of acetylacetone (68.6 µmol, 310 equiv.) were added and the solution sonicated for 5 min. After 1 h of reaction, monitored via LCMS, the solution was extracted five times with hexane (2x the reaction volume) and purified via semipreparative HPLC (Kromasil 300-10-C4 10x250 mm semiprep, 5-95% buffer B (ACN +0.08% TFA) in buffer A (MQ-H_2O +0.1% TFA) in 50 min) at 60 °C. A yield of 1.96 mg (64%, 0.14 µmol) could be obtained (see Figure S 36).

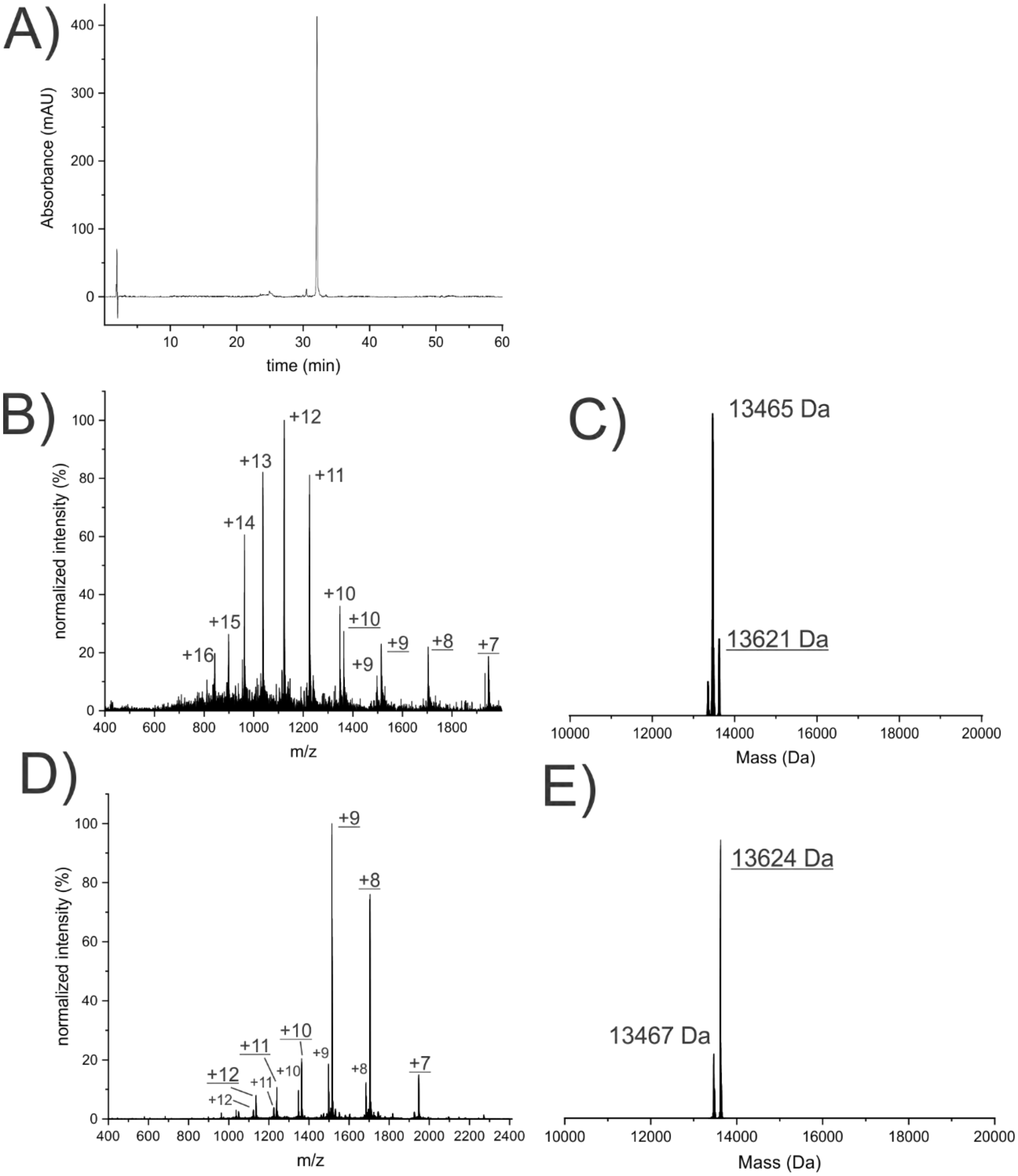

Figure S 36 A) HPLC chromatogram (5-95% B in 40 min, 60 °C) of G-CSF 1-124 selenoester **28**; B) ESI-MS direct injection from low resolution Waters 3100 mass detector and C) deconvoluted mass; D) ESI-MS from high resolution LTQ Orbitrap Velos system and E) deconvoluted mass; expected mass 13624 Da; charge states and deconvoluted mass corresponding to the expected mass are underlined. The observed side product is presumably formed during ionization due to fragmentation of the selenoester. Analysis on a high resolution instrument (D and E) shows mainly the expected mass (see also main manuscript).

8. Native chemical ligation

8.1. pH variation ligation

Gly(SeAUX)-peptide **16G** (0.05 mg, 0.05 µmol, 1 equiv.) and LYRAG-SePh **17G** (0.06 mg, 0.08 µmol, 1.5 equiv.) were dissolved in ligation buffer (6 M Gdn-HCl, 200 mM bis-tris, pH as indicated) at a concentration of 0.5 mM. Samples were taken after the indicated time points, and directly analyzed via LC/MS (Figure S 37).

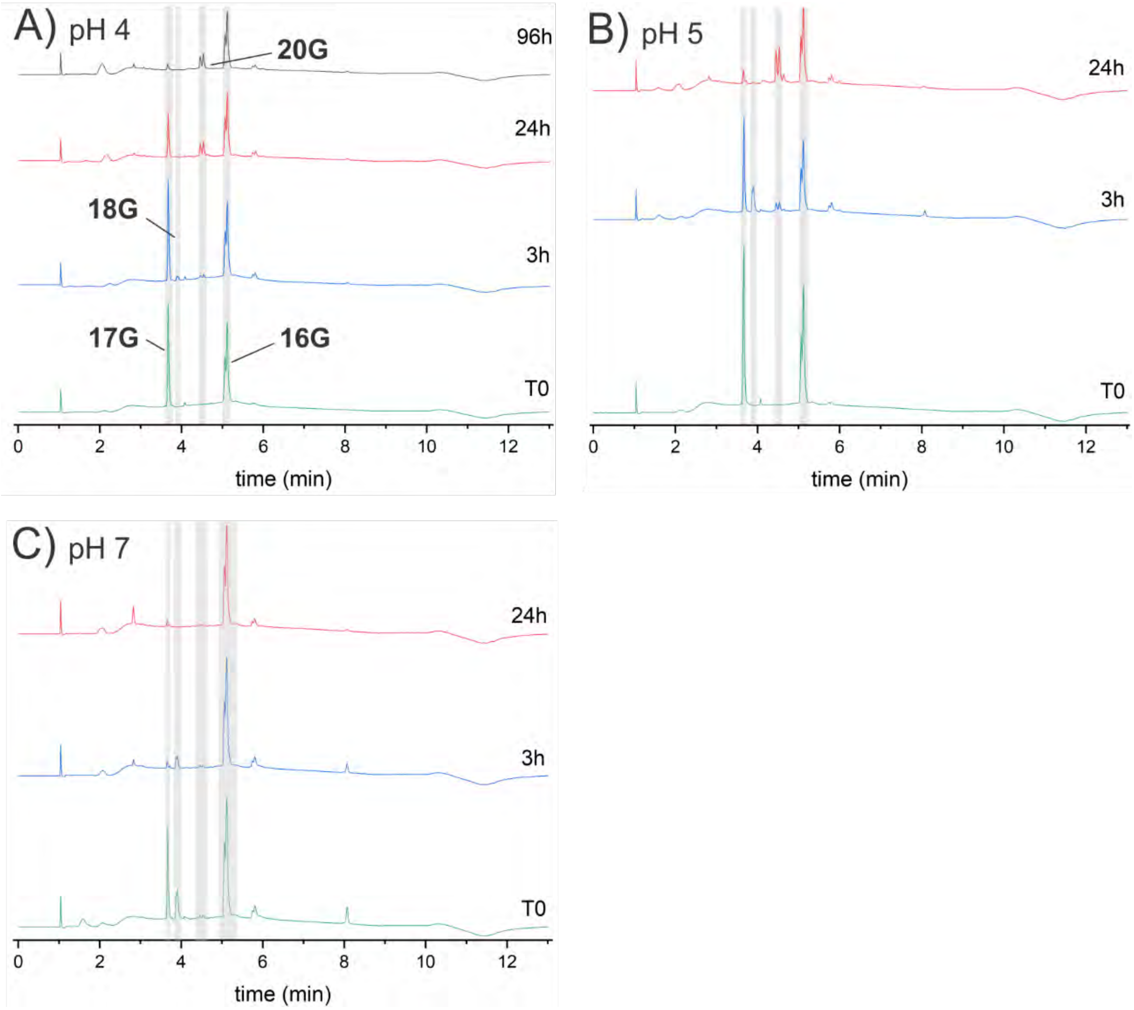

Figure S 37 HPLC chromatogram (1-90% of buffer B (ACN + 0.08% formic acid) in buffer A (MQ-H_2O + 0.1% formic acid) in 6.5 min) ligation of **16G** and **17G** A) at pH 4, B) pH 5 and C) pH 7

8.2. Gly(SeAUX) model ligation peptides **20X**

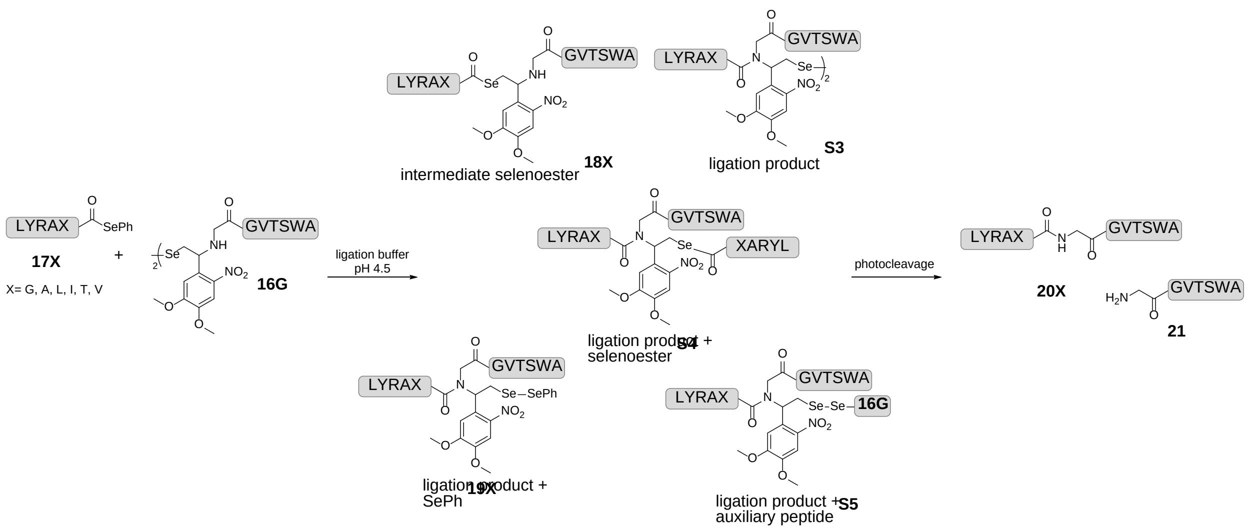

Scheme S 5 Starting material, observed intermediates and products of ligation of Gly(SeAUX)-peptide **16G** with selenoester **17X**

Detailed reaction conditions are reported in the experimental section of the main manuscript.

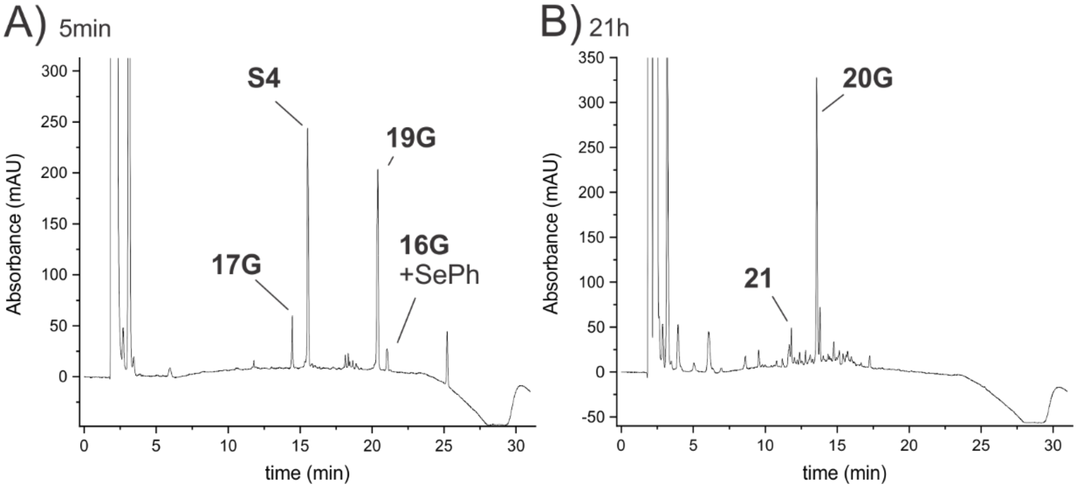

Figure S 38 HPLC chromatogram (5-45% B in 17 min) ligation Gly(SeAUX)-peptide **16G** and selenoester **17G**; withdrawn after A) 5 min, sample diluted in ligation buffer and analyzed, and B) 21 h, sample diluted in ligation buffer, photocleaved, 2.5% hydrazine added and analyzed

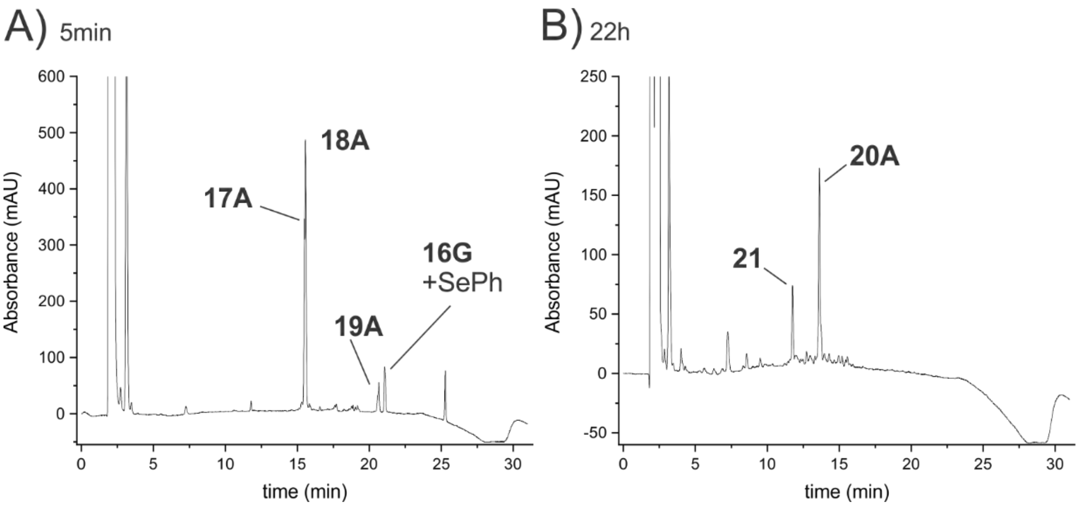

Figure S 39 HPLC chromatogram (5-45% B in 17 min) ligation Gly(SeAUX)-peptide **16G** and selenoester **17A**; withdrawn after A) 5 min, sample diluted in ligation buffer and analyzed, and B) 22 h, sample diluted in ligation buffer, photocleaved and 2.5% hydrazine added

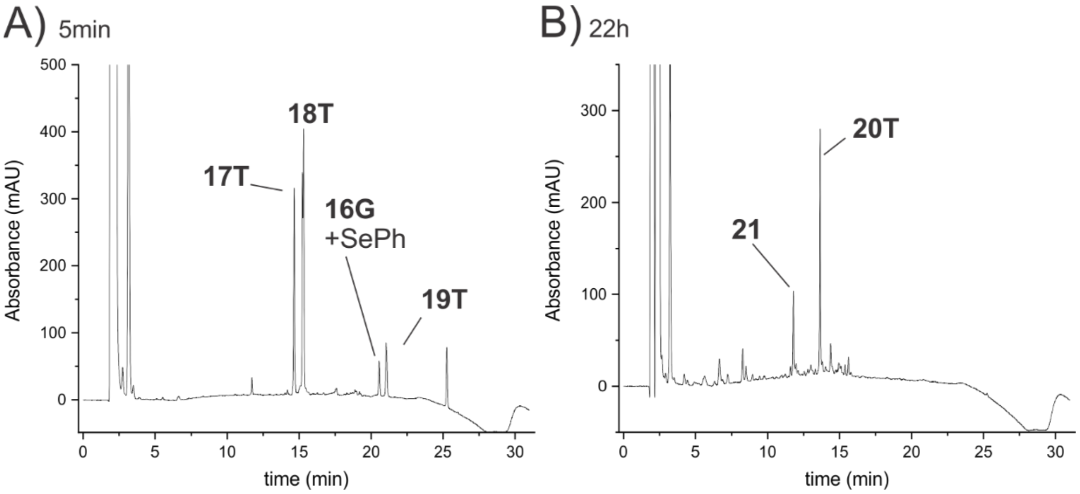

Figure S 40 HPLC chromatogram (5-45% B in 17 min) ligation Gly(SeAUX)-peptide **16G** and selenoester **17T**; withdrawn after A) 5 min, sample diluted in ligation buffer and analyzed, and B) 22 h, sample diluted in ligation buffer, photocleaved and 2.5% hydrazine added

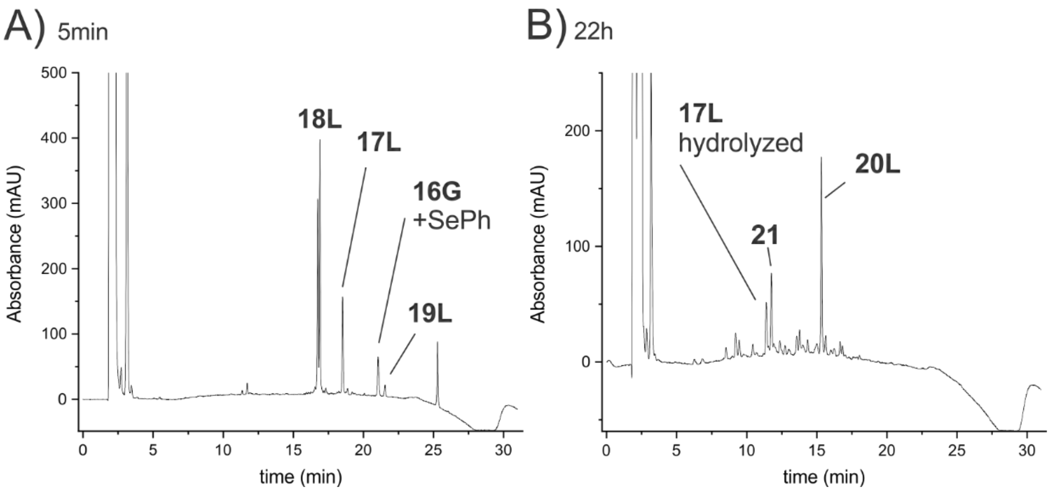

Figure S 41 HPLC chromatogram (5-45% B in 17 min) ligation Gly(SeAUX)-peptide **16G** and selenoester **17L**; withdrawn after A) 5 min, sample diluted in ligation buffer and analyzed, and B) 22 h, sample diluted in ligation buffer, photocleaved and 2.5% hydrazine added

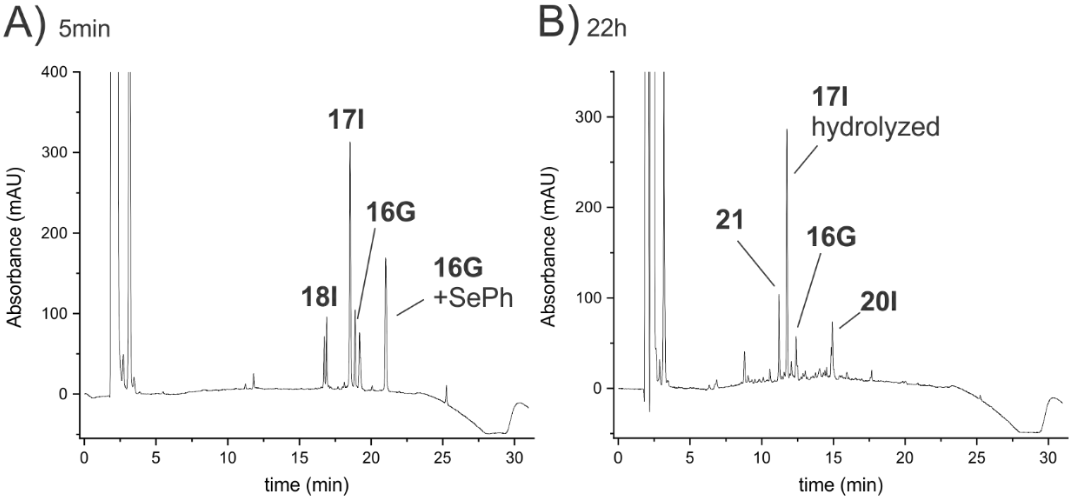

Figure S 42 HPLC chromatogram (5-45% B in 17 min) ligation Gly(SeAUX)-peptide **16G** and selenoester **17I**; withdrawn after A) 5 min, sample diluted in ligation buffer and analyzed, and B) 22 h, sample diluted in ligation buffer, photocleaved and 2.5% hydrazine added

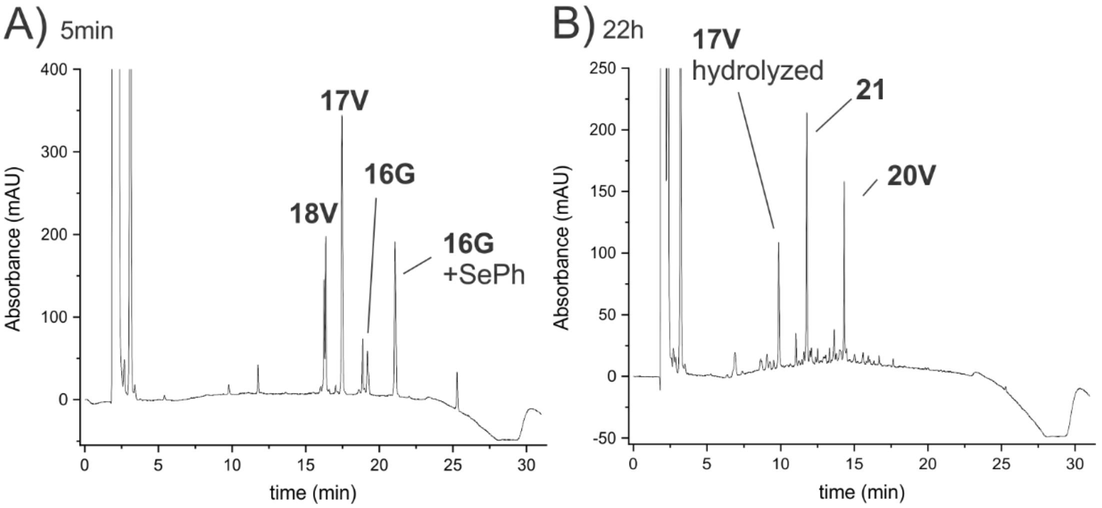

Figure S 43 HPLC chromatogram (5-45% B in 17 min) ligation Gly(SeAUX)-peptide **16G** and selenoester **17V**; withdrawn after A) 5 min, sample diluted in ligation buffer and analyzed, and B) 22 h, sample diluted in ligation buffer, photocleaved and 2.5% hydrazine added

8.3. Ala(SeAUX) model ligation peptides **24X**

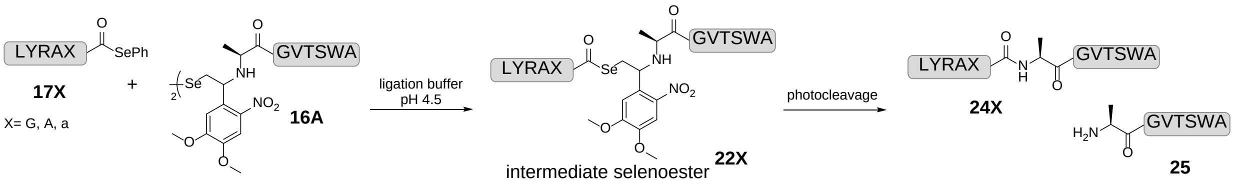

Scheme S 6 Starting material, observed intermediate and products of ligation of Ala(SeAUX)-peptide **16A** with selenoester **17X**

Ala(SeAUX)-peptide **16A** (0.05 mg, 0.05 µmol, 1 equiv.) and selenoester **17X** (0.08 mg, 0.1 µmol, 2 equiv.) were dissolved in ligation buffer (6 M Gdn-HCl, 200 mM sodium acetate, pH 4.5; degassed with argon) at a concentration of 1.7 mM (30 µL, calculated for the reduced monomer). Then a solution of TCEP (15 mM, 0.5 equiv. based on TCEP concentration) and DPDS (50 mM) in ligation buffer (agitated beforehand for ca. 15 min) were added. After 5 min 1.5 µL were taken from the reaction solution, diluted with 18.5 µL ligation buffer and analyzed via HPLC and LC-MS. The remaining reaction solution was shaken at 37 °C. For all further time points: 1.5 µL sample were taken, diluted in 10 µL ligation buffer and irradiated with UV light (365 nm, 166 mW/cm^2) for 3 min, after which 10 µL of 2.5% hydrazine (in MQ-H_2O) were added and samples measured via HPLC (Figure S 44 - Figure S 46 and Scheme S 6) and LC-MS. For samples that were treated with 5% acetic acid in DMF, 3 µL of the reaction mixture were withdrawn after 1 h and 20 µL of 5% AcOH in DMF added for 20 h. The solution was then diluted with 1 mL MQ-H_2O and lyophilized, after which the residue was dissolved in 20 µL of ligation buffer, irradiated with UV light (365 nm, 166 mW/cm^2) for 3 min, 20 µL of 2.5% hydrazine (in MQ-H_2O) added and samples measured via HPLC (Figure S 44-Figure S 46 and Scheme S 6) and LC-MS.

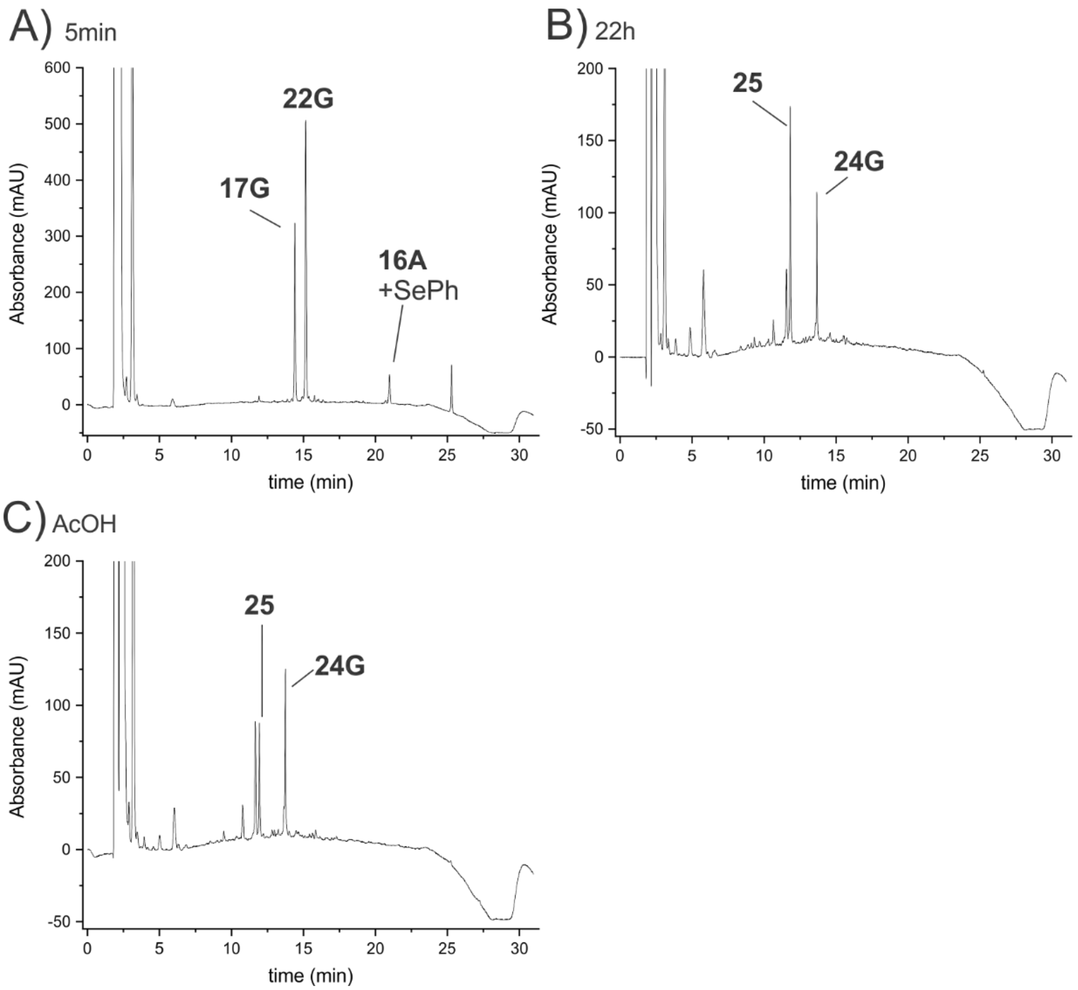

Figure S 44 HPLC chromatogram (5-45% B in 17 min) ligation Ala(SeAUX)-peptide **16A** and selenoester **17G**; withdrawn after A) 5 min, sample diluted in ligation buffer and analyzed, B) 22 h, sample diluted in ligation buffer, photocleaved and 2.5% hydrazine added and C) after treatment with AcOH in DMF

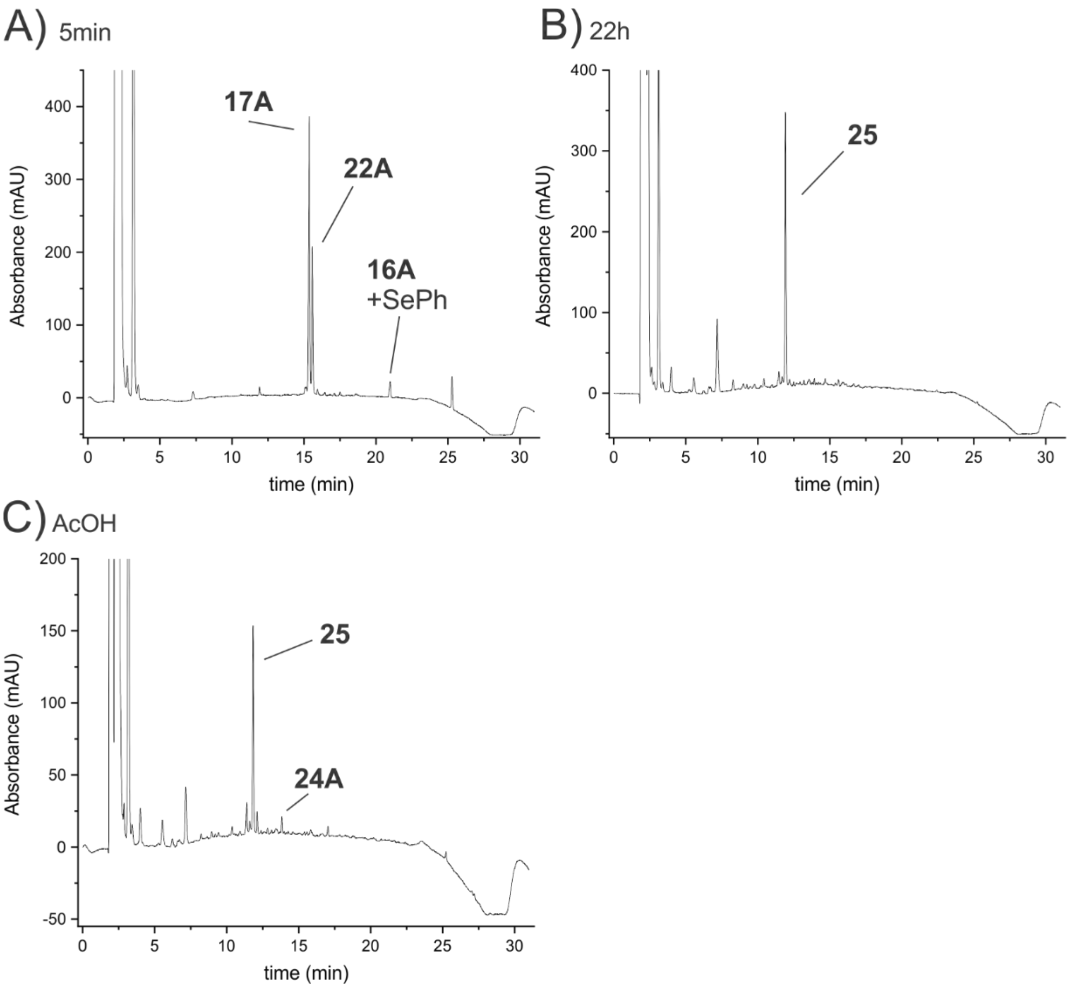

Figure S 45 HPLC chromatogram (5-45% B in 17 min) ligation Ala(SeAUX)-peptide **16A** and selenoester **17A**; withdrawn after A) 5 min, sample diluted in ligation buffer and analyzed, B) 22 h, sample diluted in ligation buffer, photocleaved and 2.5% hydrazine added and C) after treatment with AcOH in DMF

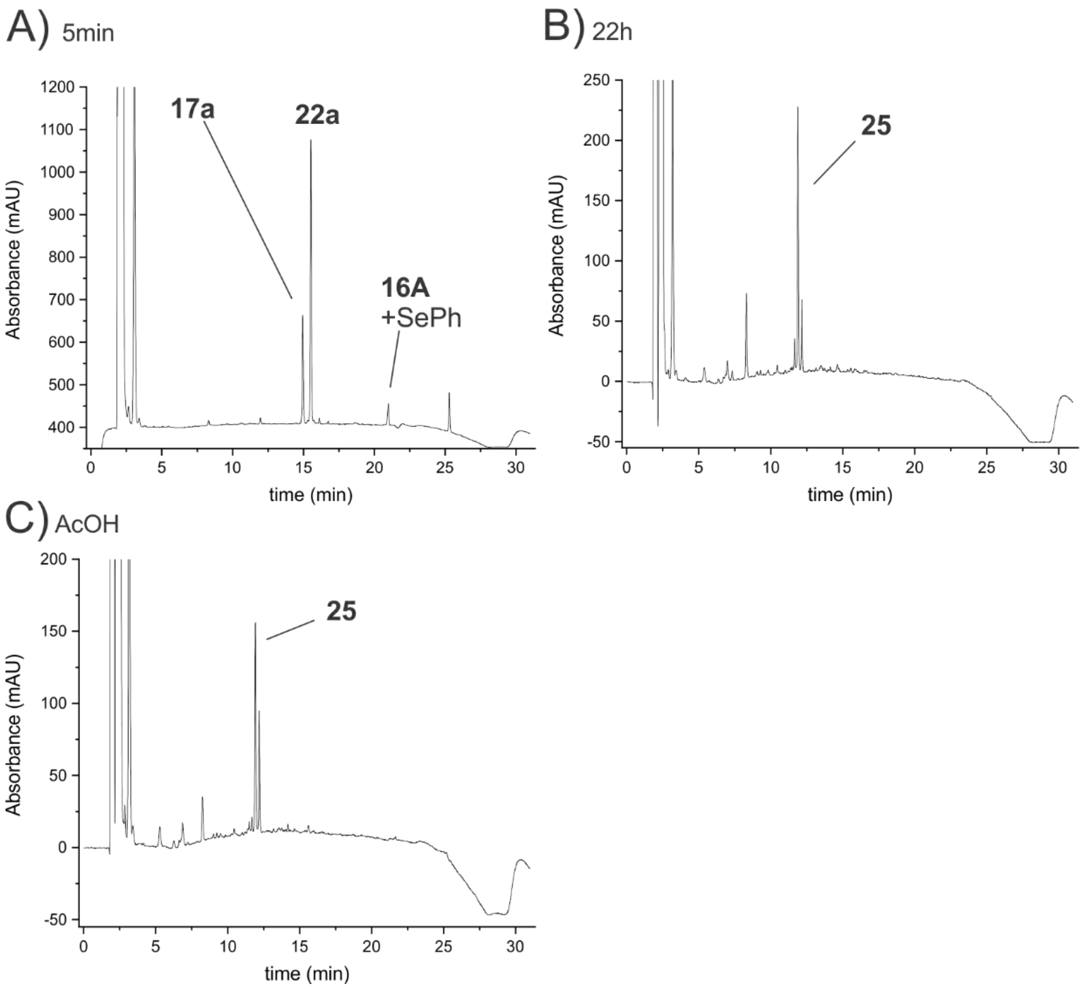

Figure S 46 HPLC chromatogram (5-45% B in 17 min) ligation Ala(SeAUX)-peptide **16A** and selenoester **17a**; withdrawn after A) 5 min, sample diluted in ligation buffer and analyzed, B) 22 h, sample diluted in ligation buffer, photocleaved and 2.5% hydrazine added and C) after treatment with AcOH in DMF

8.4. Gly(SeAUX) model ligation without TCEP peptides **20X**

Auxiliary peptides **16G** (0.05mg, 0.05 µmol, 1 equiv.) and selenoester **17X** (0.08 mg, 0.1 µmol, 2 equiv.) were dissolved in ligation buffer (6 M Gdn-HCl, 200 mM sodium acetate, pH 4.5; degassed with argon) at a concentration of 1.7 mM (30 µL, calculated for the reduced monomer). After 5 min 1.5 µL were taken from the reaction solution, diluted with 18.5 µL ligation buffer and analyzed via HPLC and LC-MS. The remaining reaction solution was shaken at 37 °C. After the indicated reaction time, 1.5 µL sample were taken, diluted in 10 µL ligation buffer and irradiated with UV light (365 nm, 166 mW/cm^2) for 3 min, after which 10 µL of 2.5% hydrazine (in MQ-H_2O) were added and samples measured via HPLC (see Figure S 47-Figure S 52 and Scheme S 5) and LC-MS.

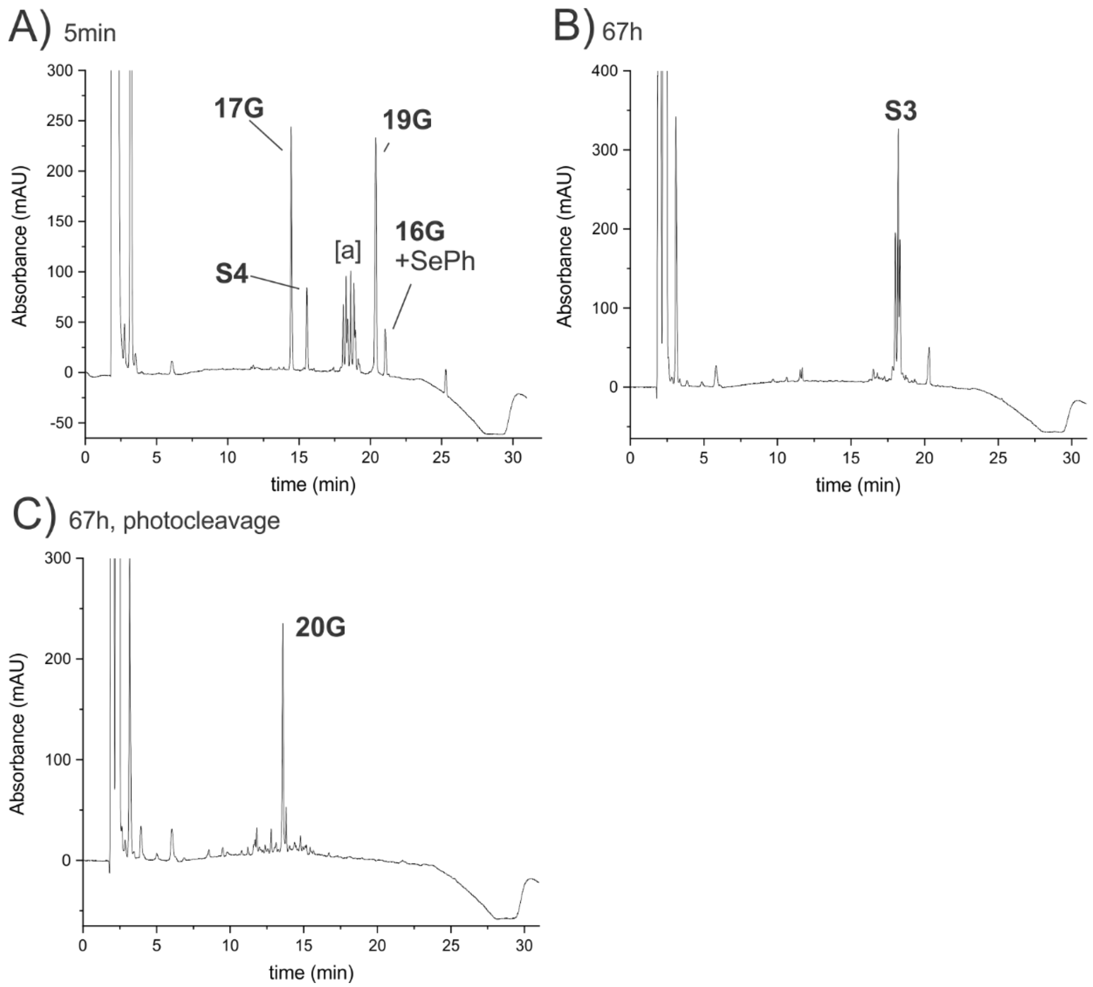

Figure S 47 HPLC chromatogram (5-45% B in 17 min) ligation Gly(SeAUX)-peptide **16G** and selenoester **17G** without addition of TCEP/DPDS; withdrawn after A) 5 min, sample diluted in ligation buffer and analyzed, and B) 67 h, sample diluted in ligation buffer, 2.5% hydrazine added and analyzed; C) 67 h, sample diluted in ligation buffer, photocleaved, 2.5% hydrazine added and analyzed; [a] mixture of **S3**, **S5** and **16G**

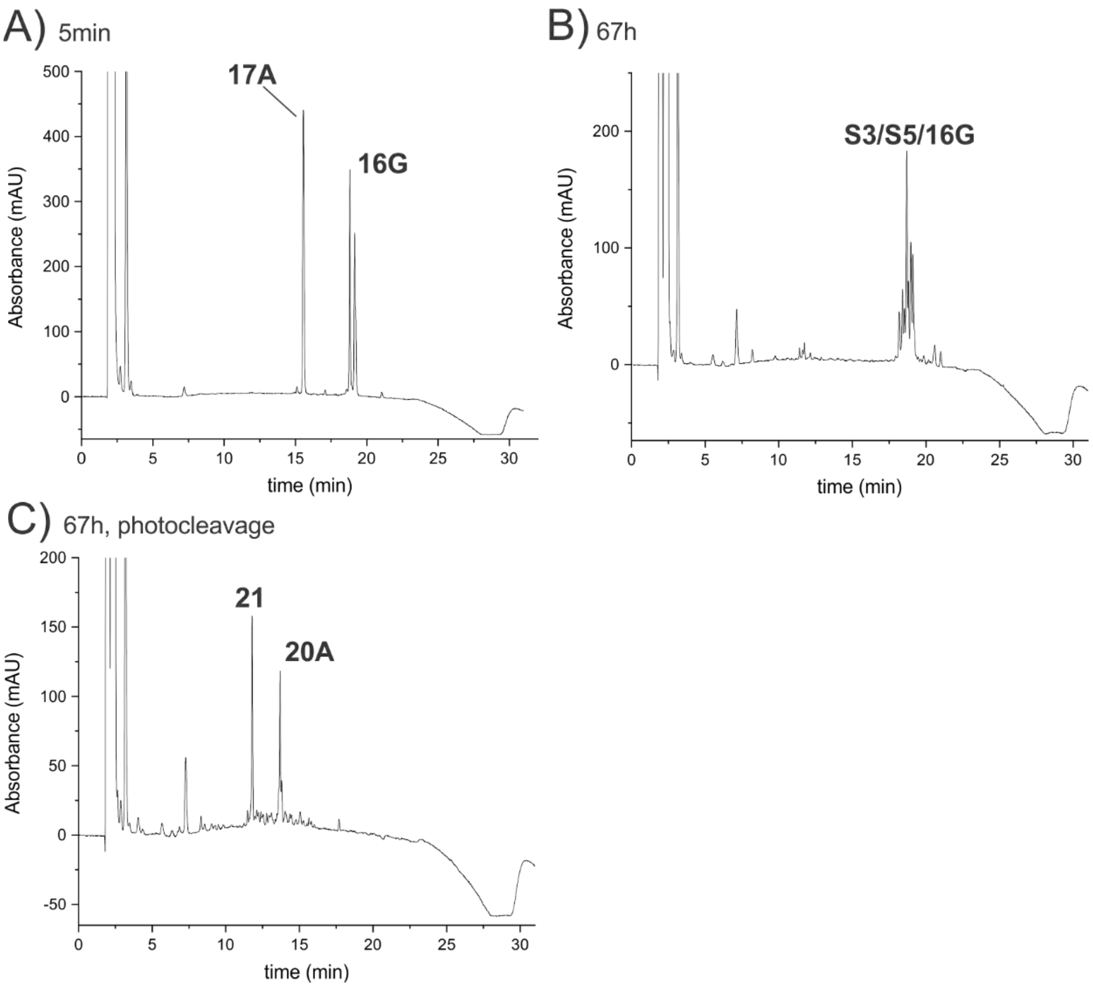

Figure S 48 HPLC chromatogram (5-45% B in 17 min) ligation Gly(SeAUX)-peptide **16G** and selenoester **17A** without addition of TCEP/DPDS; withdrawn after A) 5 min, sample diluted in ligation buffer and analyzed, and B) 67 h, sample diluted in ligation buffer, 2.5% hydrazine added and analyzed; C) 67 h, sample diluted in ligation buffer, photocleaved, 2.5% hydrazine added and analyzed

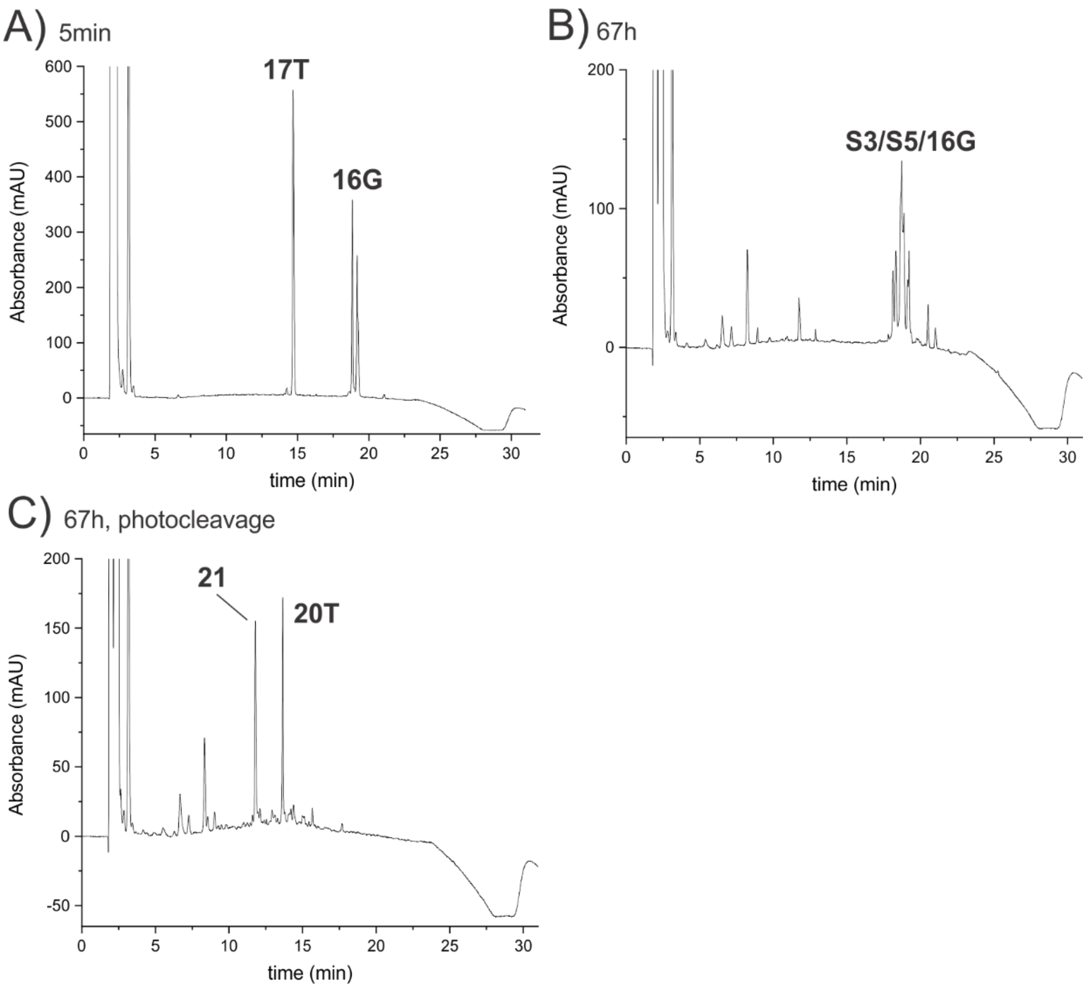

Figure S 49 HPLC chromatogram (5-45% B in 17 min) ligation Gly(SeAUX)-peptide **16G** and selenoester **17T** without addition of TCEP/DPDS; withdrawn after A) 5 min, sample diluted in ligation buffer and analyzed, and B) 67 h, sample diluted in ligation buffer, 2.5% hydrazine added and analyzed; C) 67 h, sample diluted in ligation buffer, photocleaved, 2.5% hydrazine added and analyzed

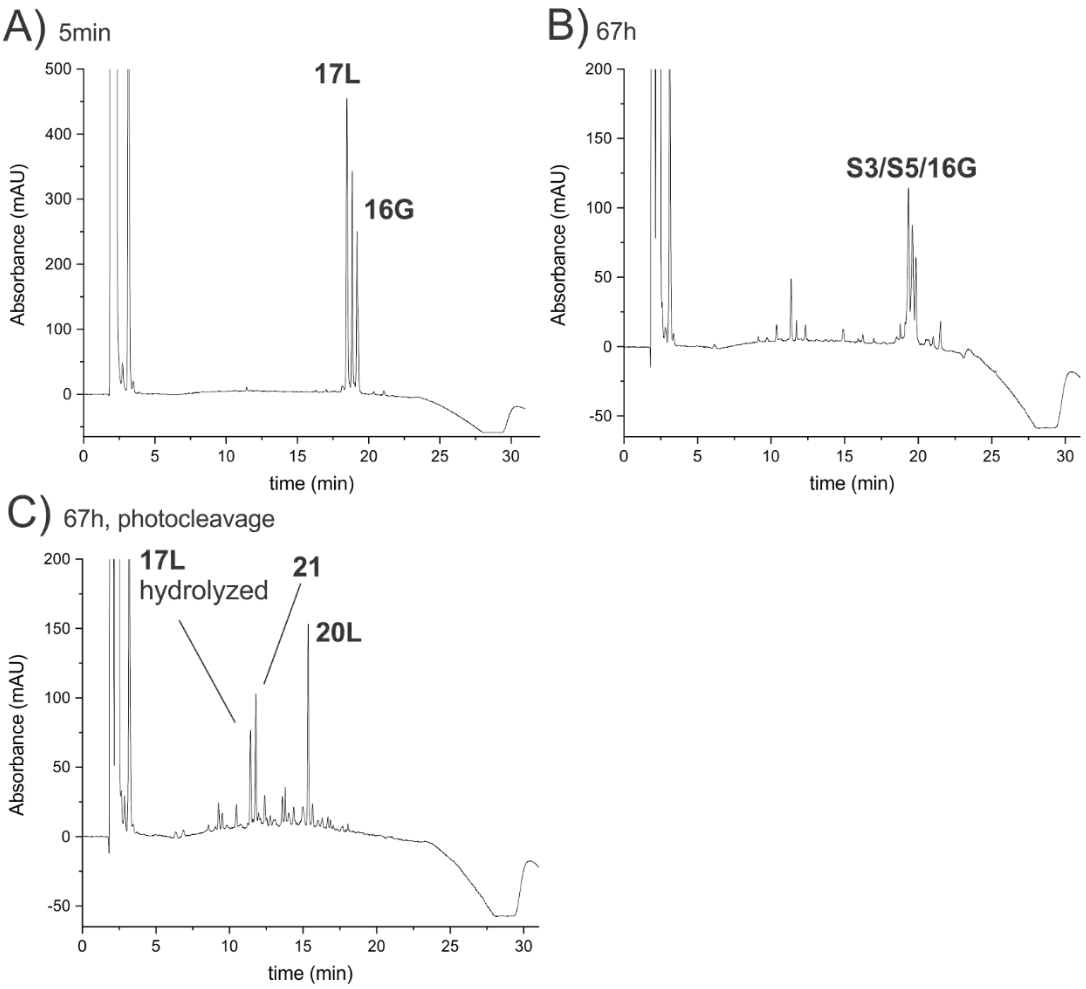

Figure S 50 HPLC chromatogram (5-45% B in 17 min) ligation Gly(SeAUX)-peptide **16G** and selenoester **17L** without addition of TCEP/DPDS; withdrawn after A) 5 min, sample diluted in ligation buffer and analyzed, and B) 67 h, sample diluted in ligation buffer, 2.5% hydrazine added and analyzed; C) 67 h, sample diluted in ligation buffer, photocleaved, 2.5% hydrazine added and analyzed

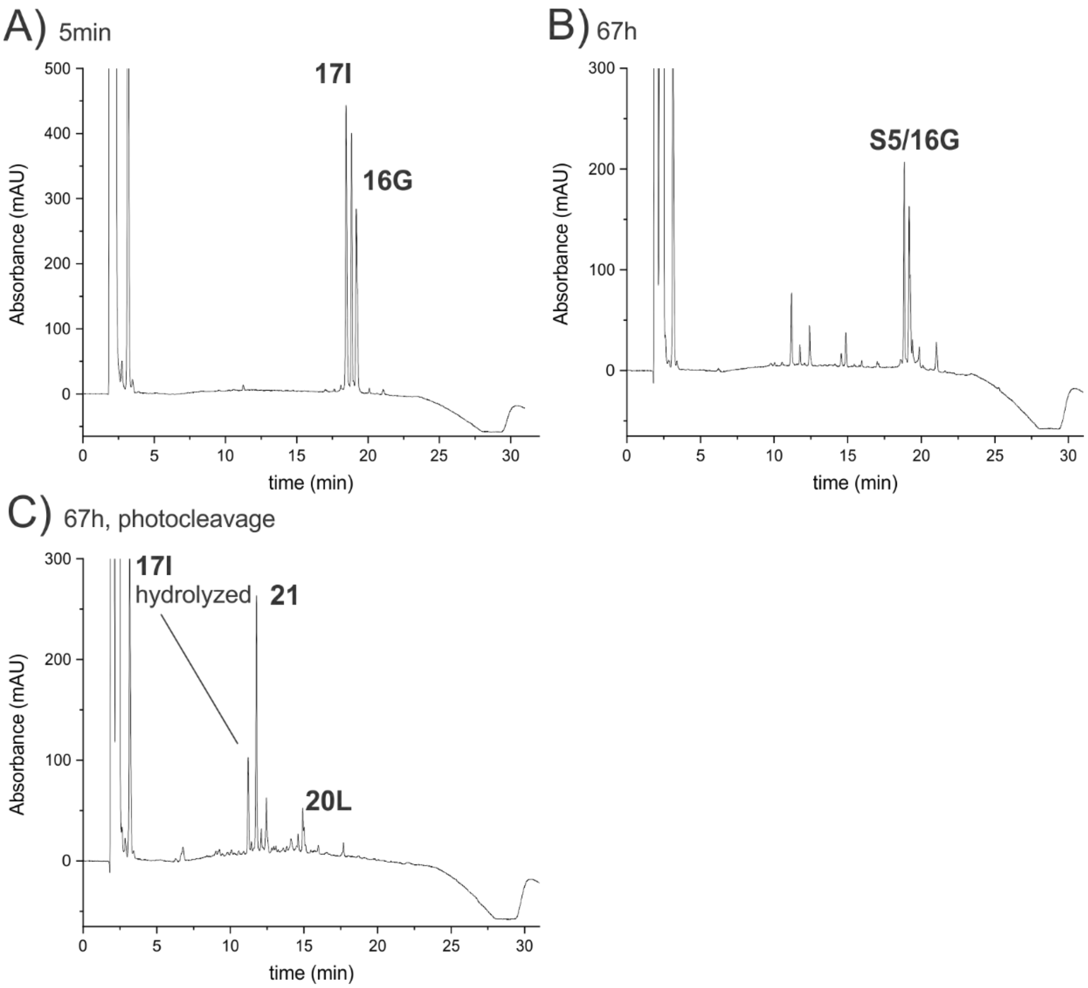

Figure S 51 HPLC chromatogram (5-45% B in 17 min) ligation Gly(SeAUX)-peptide **16G** and selenoester **17I** without addition of TCEP/DPDS; withdrawn after A) 5 min, sample diluted in ligation buffer and analyzed, and B) 67 h, sample diluted in ligation buffer, 2.5% hydrazine added and analyzed; C) 67 h, sample diluted in ligation buffer, photocleaved, 2.5% hydrazine added and analyzed

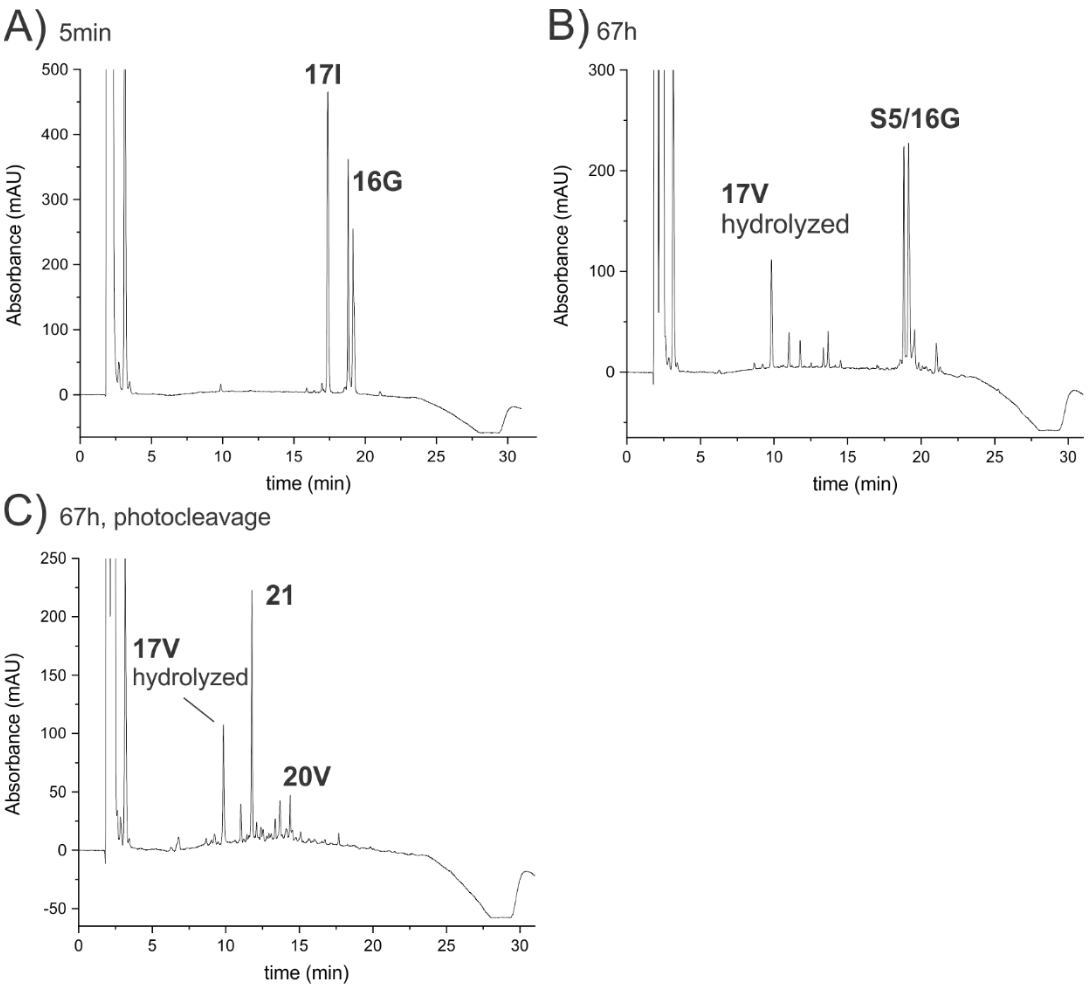

Figure S 52 HPLC chromatogram (5-45% B in 17 min) ligation Gly(SeAUX)-peptide **16G** and selenoester **17V** without addition of TCEP/DPDS; withdrawn after A) 5 min, sample diluted in ligation buffer and analyzed, and B) 67 h, sample diluted in ligation buffer, 2.5% hydrazine added and analyzed; C) 67 h, sample diluted in ligation buffer, photocleaved, 2.5% hydrazine added and analyzed

Table S 1 Comparison of product yields with and without the addition of TCEP and DPDS to the ligation reaction

Product	Product yield without TCEP/DPDS [a]	Product yield with TCEP/DPDS[a, b]
**20G** [c]	94%	92%
**20L** [d]	61%	73%
**20T** [d]	49%	74%
**20A** [d]	38%	71%
**20V** [d]	13%	38%
**20I** [d]	9%	10 %

[a] sample irradiated with UV light for 3 min, then 2.5% hydrazine added; [b] 0.5 equiv. of TCEP/DPDS added; [c] ligation time with TCEP/DPDS 21 h, without 67 h; [d] Ligation time with TCEP/DPDS 22 h, without 67 h

8.5. Expressed protein selenoester ligation, G-CSF 1-124 selenoester **28** and Gly(SeAUX)-G-CSF 126-174 **29**

Ligation buffer (6 M Gdn-HCl, 200 mM sodium acetate, pH 4.5; 47 µL degassed with argon) was added to a mixture of selenoester G-CSF 1-124 **28** (0.68 mg, 0.05 µmol) and Gly(SeAUX)-G-CSF 126-174 **29** peptide (0.7 mg, 0.13 µmol, 2.5 equiv.) at a concentration of 1.1 mM. A solution of TCEP/DPDS was added (4.2 µL, 15 mM TCEP, 50 mM DPDS, 0.5 equiv. based on Gly(SeAUX)-G-CSF 126-174 peptide monomer) and the mixture sonicated for 5 min, after which the reaction was shaken at 37 °C (Figure S 53 A). After 51 h the reaction solution was diluted with 40 µL of 60 mM TCEP in ligation buffer pH 4.5, incubated for 10 min, followed by irradiation with UV light (365 nm, 166 mW/cm^2) for 5 min (Figure S 53 B). Then, 80 µL of a 2.5% hydrazine solution (in MQ-H_2O) were added, and the solution purified via HPLC (Waters XBridge BEH C4 column, 3.5 µm, 4.6 mm x 150 mm) with a gradient of 5-95% buffer B (ACN +0.08% TFA) in buffer A (MQ-H_2O +0.1% TFA) at 60 °C over 40 min, giving the final product **30** (0.2 mg, 0.011 µmol) in 20% yield (see Figure S 53).

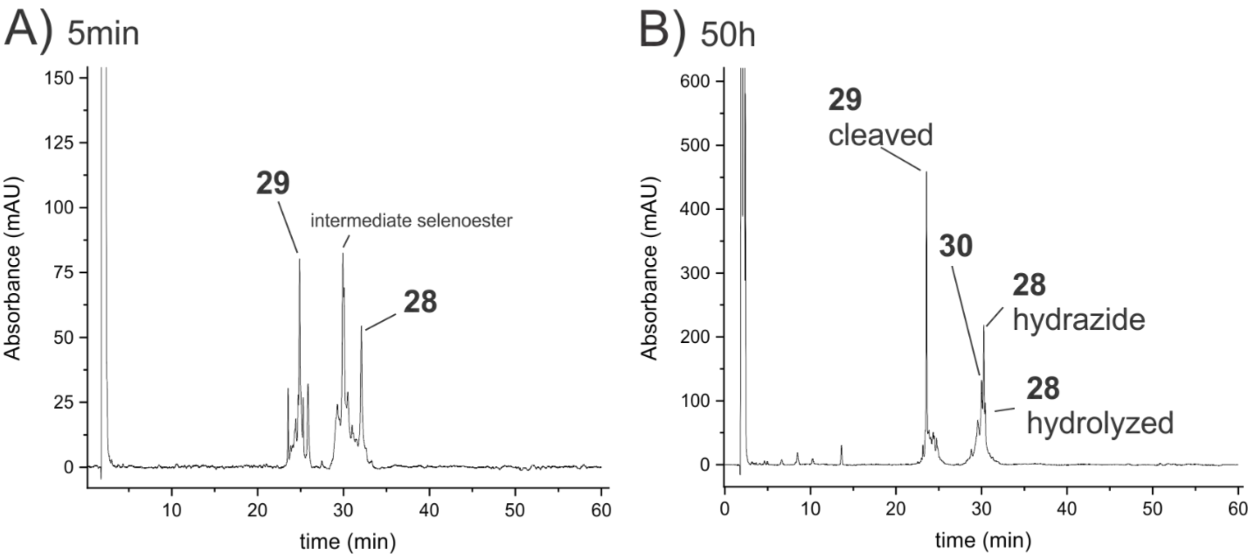

Figure S 53 HPLC chromatogram (5-95% B in 40 min, 60 °C) of expressed selenoester ligation G-CSF 1-124 selenoester **28** and Gly(SeAUX)-G-CSF 126-174 **29** A) after 5 min, direct analysis, B) after 50 h, TCEP added, photocleavage and quenched with hydrazine

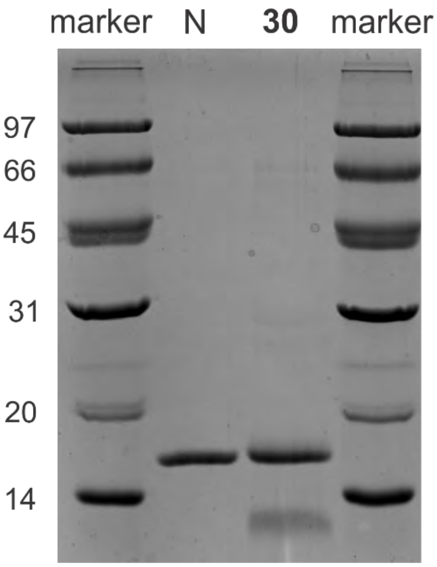

Figure S 54 SDS-PAGE of final product G-CSF 1-174 **30**; marker: low molecular weight (LMW) marker, N: Neupogen®; The lower band in lane **30** at approx. 13.5 kDa is an inseparable contamination of G-CSF 1-124-$NHNH_2$ formed during hydrazine quenching of the ligation reaction.

9. Racemization experiments Ala(SeAUX)-peptide **16A**

Peptides **Da-25** and **25** were synthesized as described above (see section 6.7). For the photocleavage, Ala(SeAUX)-peptide **16A** was dissolved in 6 M Gdn-HCl, 200 mM sodium acetate buffer, pH 4.5 and irradiated with 365 nm UV light for 5 min (see Figure S 55).

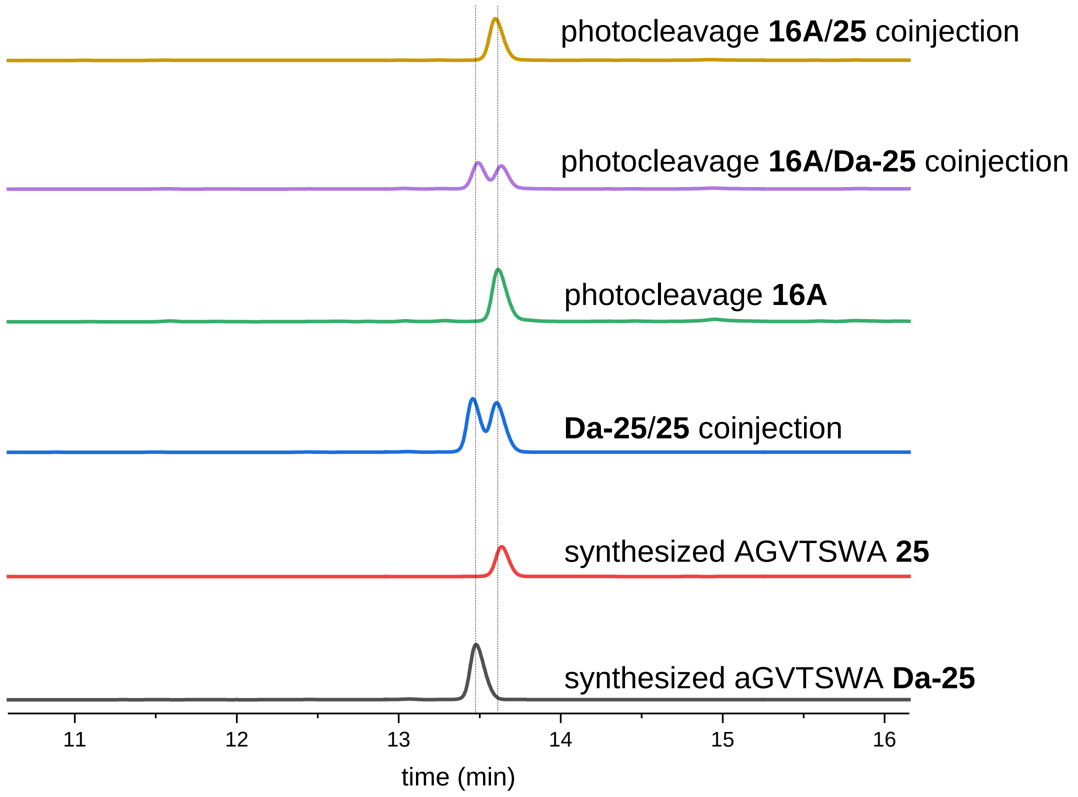

Figure S 55 Co-injection experiments of manually synthesized peptides **25**, **Da-25** and photocleaved Ala(SeAUX)-peptide **16A**

10. Abbreviations

MQ-H_2O – Milli-Q water
DPDS – Diphenyl diselenide
TIPS – Triisopropyl silane
DMDS – Dimethyl disulfide
DIPEA – diisopropyl ethylamine
TCEP - tris(2-carboxyethyl)phosphine
DTT – Dithiothreitol
NMR – Nuclear magnetic resonance
HSQC – Heteronuclear single quantum coherence

11. References

[11] L. Kerul, M. Schrems, A. Schmid, R. Meli, C. F. W. Becker, C. Bello, *Angew. Chem. Int. Ed.* **2022**, *61*, e202206116.

[21] Y. Li, J. Liu, Q. Zhou, J. Zhao, P. Wang, *Chin. J. Chem .* **2021**, *39*, 1861-1866.

[26] a) E. Schué, A. Kopyshev, J.-F. Lutz, H. G. Börner, *Journal of Polymer Science* **2020**, *58*, 154-162; b) Handoko, S. Satishkumar, N. R. Panigrahi, P. S. Arora, *J. Am. Chem. Soc.* **2019**, *141*, 15977-15985; c) D. L. J. Clive, S. Hisaindee, D. M. Coltart, *J. Org. Chem.* **2003**, *68*, 9247-9254; d) M. J. Bird, P. E. Dawson, *Pept. Sci.* **2022**, *114*, e24268.
